# Identification of novel genomic regions associated with nine mineral elements in Chinese winter wheat grain

**DOI:** 10.1186/s12870-021-03105-3

**Published:** 2021-07-01

**Authors:** Wei Wang, Hong Guo, Chongning Wu, Hui Yu, Xiaokang Li, Guangfeng Chen, Jichun Tian, Zhiying Deng

**Affiliations:** 1 State Key Laboratory of Crop Biology, Key Laboratory of Crop Biology of Shandong Province, Group of Wheat Quality and Molecular Breeding, College of Agronomy, Shandong Agricultural University, Tai’an, Shandong 271000 P.R. China; 2 Handan Academy of Agricultural Sciences, Handan, Hebei 056000 P.R. China; 3 College of Ecology and Garden Architecture, Dezhou University, Dezhou, Shandong 253023 P.R. China

**Keywords:** Wheat grain, Nine mineral elements, Genome wide association study, Candidate gene

## Abstract

**Background:**

Mineral elements are important for maintaining good human health besides heavy metals. Mining genes that control mineral elements are paramount for improving their accumulation in the wheat grain. Although previous studies have reported some loci for beneficial trace elements, they have mainly focused on Zn and Fe content. However, little information is available regarding the genetic loci differences in dissecting synchronous accumulation of multiple mineral elements in wheat grains, including beneficial and heavy elements. Therefore, a genome-wide association study (GWAS) was conducted on 205 wheat accessions with 24,355 single nucleotide polymorphisms (SNPs) to identify important loci and candidate genes for controlling Ca, Fe, Zn, Se, Cu, Mn, Cd, As, and Pb accumulation in wheat grains.

**Results:**

A total of 101 marker-trait associations (MTAs) (*P* < 10^–5^) loci affecting the content of nine mineral elements was identified on chromosomes 1B, 1D, 2A, 2B, 3A, 3B, 3D, 4A, 4B, 5A, 5B, 5D, 6B, 7A, 7B, and 7D. Among these, 17 major MTAs loci for the nine mineral elements were located, and four MTAs loci (*P* < 10^–5^) were found on chromosomes 1B, 6B, 7B, and 7D. Eight multi-effect MTAs loci were detected that are responsible for the control of more than one trait, mainly distributed on chromosomes 3B, 7B, and 5A. Furthermore, sixteen candidate genes controlling Ca, Fe, Zn, Se, Cd, and Pb were predicted, whose functions were primarily related to ion binding, including metals, Fe, Ca, Cu, Mg, and Zn, ATP binding, ATPase activity, DNA binding, RNA binding, and protein kinase activity.

**Conclusions:**

Our study indicated the existence of gene interactions among mineral elements based on multi-effect MTAs loci and candidate genes. Meanwhile this study provided new insights into the genetic control of mineral element concentrations, and the important loci and genes identified may contribute to the rapid development of beneficial mineral elements and a reduced content of harmful heavy metals in wheat grain.

**Supplementary Information:**

The online version contains supplementary material available at 10.1186/s12870-021-03105-3.

## Background

Wheat is one of the most important crops grown globally, and as a staple food, it provides approximately 20% of the calories and 40% of the protein consumed worldwide. With the improvement in living standards, the nutritional quality of wheat grain has become increasingly important. There are many mineral elements in the wheat grain, that are important sources of trace elements in the human body. Although some important mineral elements can be found in wheat grains, such as iron (Fe), zinc (Zn), and selenium (Se), their content is relatively low and their bioavailability is poor, which leads to the potential threat of mineral-element nutritional deficiencies in developing countries around the world [1].

More than one-third of the women and children in economically underdeveloped countries do not receive key trace elements, such as Fe, Zn, and iodine (I), in sufficient amounts to sustain a healthy condition. Specifically, more than two-thirds of the population in China suffers from Se deficiency [2]. Therefore, effectively improving the content of beneficial mineral elements in the wheat grain has become an issue of the highest priority in plant breeding programs.

In addition to some beneficial mineral elements, wheat also contains some heavy metals because of environmental pollution, such as Cd, Pb, and As, whose excessive intake may cause damage to human health, including cancer of the prostate, lungs, and testes, as well as kidney tubule damage [3, 4]. Therefore, in recent years, the heavy metal content in food has become a focus of attention for the society as a whole. The essential and toxic nature of several dietary trace elements, including Cr, Co, Cu, Fe, Mg, Mn, Se, and Zn, have been thoroughly investigated [5–10].

Micronutrient deficiencies (especially Zn and Fe), are responsible for the effects of malnutrition on a very large proportion of the world population. In developing countries, most people rely on cereal grains as their staple food, and malnutrition has been detected among children because of micronutrient deficiencies, a phenomenon that has been described as the ‘hidden hunger’ [1, 11, 12]. To solve malnutrition caused by the lack of beneficial mineral elements in food, biofortification of wheat grain has become a routine in the food industry, especially for Zn, Fe, and Se. Since 2003, scientists from various international agricultural research institutes have begun to implement the Harvest Plus project to solve this problem by cultivating mineral-rich food crops. Generally, there are two methods used to improve micronutrient content of wheat grain, which involves agronomic practices and genetic improvement. However, agronomic practices are reputedly neither economical nor environmentally friendly [13].

Alternatively, the genetic improvement for micronutrient content such as Zn, Fe, and Se in wheat grain is an important and effective approach. In previous studies, some important genes/loci for micronutrients including, Zn, Fe, and Se, were found using quantitative trait loci (QTL) mapping and genome-wide association study (GWAS) methods. Thus, using three different sets of recombinant inbred lines (RILs) [14, 15] and QTL mapping, two major QTLs for Zn content on chromosome 7B (QGZn.cimmyt-7B_1P2 and QGZn.cimmyt-7B_1P1) and one major QTL for Fe content on chromosome 4A (QGFe.cimmyt-4A_P2) were identified, and pleiotropic QTLs were also found on chromosome 3B. Additionally, through the meta-QTL (MQTL) method, some important MQTLs for Zn and Fe content were found on chromosomes 2D, 5A, 5B, 6A, and 7A [13]. However, there were only three studies reported for the QTLs of Se content on 21 chromosomes [16–18].

Using association panels, including common wheat (*Triticum aestivum* L.), synthetic hexaploid wheat, harvest plus association panel, European wheat varieties, and spring wheat, a total of 442 marker-trait associations (MTAs) for Zn content and 287 MATs for Fe content were identified [19–27]. Of these, two highly significant MATs for Zn content were found on chromosomes 5A and 3B, and six candidate genes on 3BS were predicted to belong to the mitogen-activated protein kinase family, which is involved in protein kinase activity, protein phosphorylation, and protein transport [13]. This is related to Zn uptake and transport. On chromosome 5AL, four candidate genes were found in the bZip family and FAR1 protein, which are related to Zn biofortification [13]. Furthermore, the *Gpc-b1* gene cloned from wild wheat has been shown to increase protein, Zn, and Fe content in wheat grain [28].

Although previous studies have reported some loci for beneficial trace elements, they have mainly focused on Zn and Fe content. However, little information is available regarding the genetic loci differences in dissecting synchronous accumulation of multiple mineral elements in wheat grains, including beneficial and heavy elements. Therefore, this study used GWAS to dissect the accumulation of six beneficial elements and three harmful heavy elements using 24,355 single nucleotide polymorphisms (SNPs) genotyped from the 90 K Illumina iSelect array in a population of diverse winter wheat varieties. The objective of this study was to identify SNPs markers and candidate genes for loci associated with these traits, and improve the micronutrient content, and reduce the threat of heavy metals in the wheat grain through molecular breeding. Our results will provide the theoretical basis for improving grain micronutrient content without increasing harmful mineral elements through molecular marker-assisted selection.

## Materials and methods

### Plant material

The association mapping panel of 205 wheat genotypes for GWAS comprised 77 released cultivars, 55 landraces, including two lines from Mexico and France, and 73 breeding lines from 10 provinces representing the major winter wheat-production regions in China [29]. About thirty seeds per each of these materials were originally acquired from National Germplasm Bank, Shandong Germplasm Bank, Academy of Agricultural Sciences of different province, and wheat breeders. And then they were multiply reproduced in our research field by our Research Group of Wheat Quality breeding from Shandong Agricultural University, Shandong Province, China. The details were seen in previous published paper [29].

### Growth conditions

The seeds used for the association mapping panel were planted in the 2014, 2015, 2016, and 2017 growing seasons in experimental fields at two locations: Shandong Agricultural University, Tai’an (TA, 36°57 N 116°36E) and the Dezhou Institute of Agricultural Sciences, Dezhou (DZ, 37°45 N 116°29E). E1, E2, E3, and E4 represented the Dezhou location in 2014 (2014 DZ), Tai’an location in 2015 (2015 TA), Tai’an location in 2016 (2016 TA), and Tai’an location in 2017 (2017 TA), respectively. All experiments were laid in a completely randomised block design with two replicates in each environment. All lines were grown in 1.3 m plots with three rows spaced 25 cm apart, and 40 seeds evenly broadcast in each row. All recommended local crop management practices were followed during all the growing seasons, and no damage attributed to lodging, disease, or pests was observed.

The soil conditions at different locations are shown in Table S1. There were no significant differences in different mineral elements between the two locations. This indicated that the soil conditions at the two locations appeared to be the same.

### Phenotypic trait evaluation

#### Whole flour milling

Grain samples were washed three times with distilled water to remove any attached particles and then oven-dried at 80 °C. Dried grain samples (50 g) were milled using a whole flour experimental mill (Perten 3100 type mill, Perten Co.,Stockholm, Sweden).

### Determination of metal element content in whole flour

Whole flour (0.2 g) samples were introduced into digestion tubes for digestion with 6 mL of nitric acid (HNO_3_) in a microwave digester. The digested solutions were filtered through a 0.45 μm water-based microporous membranes after dilution to a constant volume of 50 mL with deionised water. Subsequently, the concentrations of different metal elements including, Ca, Mn, Fe, Cu, Zn, Se, As, Cd, and Pb, were determined using inductively coupled plasma-atomic emission spectrometry (ICP-MS, Thermo Fisher, iCAP Qc). The standard curves for the different metal elements are shown in Fig. S1, and the correlation linear valuer [2]. was between 0.9977 and 0.9999. The recovery rates of all the elements ranged between 80 and 120%.

### Statistical analysis

An analysis of variance (ANOVA) and correlations among phenotypic traits were conducted using the PROC GLM procedure of SAS 8.0 (SAS Institute Inc., Cary, NC, USA) and the statistical software SPSS version 17.0 (SPSS Inc., Chicago, IL, USA), respectively.

### Genome-wide association analysis

SNP markers, genotyping, and the population structure of the samples have been previously reported [30]. Based on this information, significant MTAs were identified using a mixed linear model (MLM) in TASSEL3.0. The P-value was used to determine whether a QTL was associated with a marker. The *R* [2] value was used to evaluate the magnitude of the MTA effects. The genome-wide significance threshold (P ≤ 10^–4^) was determined. SNPs with a P-value ≤ 10^–4^ were considered to be significantly associated with phenotypic traits. When an MTA locus was detected in two or more environments, it was considered a site-stable association [29].

### Candidate genes prediction for important MTAs loci associated with mineral elements

To identify the position of important MTAs loci and possible candidate genes on a physical map, significant markers detected in this study were used to identify putative candidate genes. A BLAST (Basic Local Alignment Search Tool) search was performed using the International Wheat Genome Sequencing Consortium database (IWGSC; http://www.wheatgenome.org/, 20^th^ January 2021) with the sequence of the significant SNP markers identified by GWAS. When an SNP marker sequence from the IWGSC was 100% identical to any wheat contig, the sequence was extended by 2 Mb for each marker using the IWGSC BLAST results. The extended sequence was used to run a BLAST search at the National Center for Biotechnology Information (NCBI) database (http://www.ncbi.nlm.nih.gov, 20^th^ January 2021) and Ensembl Plants (http://plants.ensembl.org/Triticum_aestivum/Tools/Blast, 20^th^ January 2021) to confirm possible candidate genes and functions.

## Results

### Phenotypic variation and correlation analysis for nine mineral elements

Extensive phenotypic variations in Ca, Mn, Fe, Cu, Zn, Se, As, Cd, and Pb were observed among the 205 winter wheat accessions across the four environments (i.e. 3 years and two locations, Table 1). Continuous distributions in the population were observed for nine mineral elements, which showed typical quantitative traits, indicating that they were genetically controlled by multiple genes.

**Table 1:** Phenotypic variation of beneficial and harmful mineral elements in wheat grain

Trait	Environment	Minimum (μg/mL)	Maximum (μg/mL)	Mean (μg/mL)	Standard deviation	Kurtosis	Skewness
Ca	E1	1 305.341 2	3 494.334 9	2 057.703 3 aA	131.193 6	0.342 9	0.487 9
	E2	943.268 1	3 851.026 7	1 671.181 2 cC	135.305 9	4.233 2	1.429 6
	E3	1 041.368 2	3 571.288 4	1 904.142 4bB	142.117 6	0.911 6	0.832 5
	E4	745.555 5	4 337.089 3	1 635.239 5cC	155.169 3	8.360 6	2.174 4
Mn	E1	72.392 9	177.832 1	114.308 9abAB	7.170 7	0.411 3	0.780 9
	E2	64.679 8	233.943 3	110.938 1bB	7.112 4	7.099 9	1.644 4
	E3	71.567 9	170.755 6	110.751 4bB	6.072 6	0.320 1	0.443 5
	E4	78.885 2	177.475 3	117.476 9aA	6.159 6	0.151 3	0.603 5
Fe	E1	49.433 0	275.962 4	122.362 9cC	14.895 5	-0.377 8	0.528 8
	E2	44.191 4	1 267.636 9	160.493 0bB	55.525 0	15.723 5	3.650 2
	E3	60.090 6	1 226.409 9	195.083 0aA	72.516 1	9.590 7	3.047 8
	E4	41.778 4	810.932 2	137.228 4bcBC	34.887 9	19.821 1	4.036 5
Cu	E1	8.789 5	20.809 0	14.541 1aA	0.854 3	-0.537 2	0.230 7
	E2	6.594 2	38.289 7	11.147 0dD	0.905 9	49.930 1	5.185 5
	E3	6.452 0	19.370 1	12.105 5cC	0.718 3	0.905 7	0.495 6
	E4	8.134 2	31.327 0	13.098 8bB	0.879 0	10.945 2	1.887 1
Zn	E1	58.830 3	391.240 3	104.609 1aA	9.345 3	53.810 6	5.514 4
	E2	40.104 5	166.760 3	81.535 0dC	7.031 0	3.050 4	1.448 9
	E3	52.568 7	156.971 2	91.933 2cB	6.348 7	1.122 6	0.796 9
	E4	61.455 9	275.946 8	99.299 3bA	8.670 5	19.452 2	3.620 9
Se	E1	0	0.004 5	0.001 6aA	0.000 9	0.501 0	0.069 0
	E2	0	0.004 5	0.000 7cB	0.000 9	1.426 0	1.729 0
	E3	0	0.005 1	0.000 9bB	0.000 9	1.299 0	1.968 0
	E4	0	0.003 6	0.000 7cB	0.000 8	1.165 0	0.767 0
As	E1	0	0.002 2	0.000 1cC	0.000 3	3.959 0	16.506 0
	E2	0	0.010 2	0.000 8bB	0.001 5	3.397 0	15.058 0
	E3	0	0.004 5	0.000 2cC	0.000 5	5.503 0	37.033 0
	E4	0	0.008 2	0.001 9aA	0.001 8	1.156 0	1.248 0
Cd	E1	0	0.017 9	0.001 3aA	0.001 4	102.407 0	8.848 0
	E2	0	0.020 3	0.000 4cC	0.001 6	135.468 0	10.852 0
	E3	0	0.014 4	0.000 8bB	0.001 2	73.888 0	7.348 0
	E4	0	0.016 1	0.0005cBC	0.001 4	86.977 0	8.520 0
Pb	E1	0	0.213 3	-	-	-	-
	E2	0	0.098 8	-	-	-	-
	E3	0	0.214 8	-	-	-	-
	E4	0	0.692 6	-	-	-	-

E1: 2014DZ; E2: 2015TA; E3: 2016TA; E4: 2017TA

The ANOVA showed significant differences for the content of Ca, Mn, Fe, Cu, Zn, Se, and Cd (*P* < 0.0001) among genotypes and environments, as well as G × E interactions (Table S2), which indicated that mineral element content was significantly affected by genotype, environment, and their interaction. Further, correlation analysis indicated that significant positive correlations were observed among Ca, Mn, Fe, Cu, and Zn, but not Se (Table 2).

**Table 2:** Correlation analysis of beneficial mineral elements

	Ca	Mn	Fe	Cu	Zn	Se
Ca	1					
Mn	0.344**	1				
Fe	0.237**	0.277**	1			
Cu	0.359**	0.575**	0.436**	1		
Zn	0.484**	0.485**	0.202**	0.537**	1	
Se	-0.085	-0.020	-0.024	0.022	-0.099	1

^**^ The correlation coefficient was very significant at *P* < 0.01 level

^**^ Correlation is significant at *P* < 0.01

### Marker–trait associations (MTAs) of beneficial mineral elements

A total of 64 MTAs (*P* < 10^–4^) for Ca content in wheat grains were detected, mainly distributed on chromosomes 1A, 2A, 2B, 3A, 3B, 5A, 5B, 5D, 6B, 7A, and 7B in the four experimental environments (Table S3). Some MTA clusters were found on chromosomes 1A, 2B, 3B, 5A, and 6B. Four MTAs were identified (*P* < 10^–5^) on chromosomes 3B and 5A (Table 3 and Fig. S2), and the Kukri_c41797_393 locus on chromosome 5A contributed to the phenotypic variation with 10.97%, as did the RFL_Contig2187_1025 locus.

**Table 3:** Genome-wide Association Mapping results of beneficial mineral elements (*P* < 10^–5^)

Trait	Env	SNP marker	Chr.	Position	*P*	*R*^2^ (%)
Ca	E4	Excalibur_c41752_392	3B	67	4.70E-05	8.92
	E4	BS00057451_51	3B	67	4.70E-05	8.92
	E4	Kukri_c41797_393	5A	53	7.13E-06	10.97
	E4	RFL_Contig2187_1025	5A	53	7.13E-06	10.97
Mn	E2	wsnp_Ex_c4561_8184576	1B	75	6.26E-05	8.33
	E2	wsnp_BF478690B_Ta_2_1	1B	75	6.72E-05	8.28
	E2	Excalibur_c5218_75	1B	75	7.28E-05	8.2
	E2	IAAV2125	1B	75	6.72E-05	8.28
	E2	IAAV6731	1B	75	6.72E-05	8.28
	E2	JD_c3116_778	1B	75	6.26E-05	8.33
	E2	Ra_c37969_549	1B	75	8.25E-05	8.05
	E2	RAC875_c19014_725	1B	75	6.26E-05	8.33
	E2	RAC875_rep_c112555_200	1B	75	6.72E-05	8.28
	E2	RAC875_rep_c119728_146	1B	75	8.92E-05	7.97
	E2	TA003725-0553	1B	75	8.92E-05	7.97
	E2	BS00022619_51	1B	75	7.72E-05	8.14
	E2	BS00022920_51	1B	75	6.72E-05	8.28
	E2	Tdurum_contig81102_102	1B	75	8.92E-05	7.97
Fe	E2	wsnp_Ex_c11913_19105189	5A	16	5.47E-05	8.75
	E2	RAC875_rep_c112368_118	5A	16	7.57E-05	8.77
	E2	Excalibur_c6326_77	6B	20	1.46E-05	10.19
	E2	RAC875_s114363_172	6B	22	5.14E-05	8.87
	E3	wsnp_Ex_c65899_64135487	7D	26	8.79E-05	8.12
	E3	wsnp_Ra_c8297_14095831	7D	26	9.09E-05	8.07
	E3	D_contig11494_202	7D	26	7.70E-05	8.25
	E3	D_F5XZDLF01ASSE2_190	7D	26	7.70E-05	8.25
	E3	Ex_c25027_535	7D	26	7.70E-05	8.25
	E3	Excalibur_c833_1405	7D	26	8.85E-05	8.1
	E3	Kukri_rep_c103404_314	7D	26	7.70E-05	8.25
	E3	BS00022449_51	7D	26	7.82E-05	8.25
	E3	BS00110124_51	7D	27	7.70E-05	8.25
	E3	BS00110642_51	7D	27	7.70E-05	8.25
	E3	D_GB5Y7FA02IDDA9_183	7D	30	7.70E-05	8.25
	E3	TA005377-1076	7D	32	6.50E-05	8.43
	E4	Excalibur_c19455_3496	7B	163	8.12E-08	16.23
	E4	Excalibur_c11062_582	7B	171	1.44E-05	10.26
	E4	Excalibur_c25090_830	7B	171	2.81E-06	12.07
	E4	RAC875_rep_c110526_229	7B	171	1.44E-05	10.26
Cu	E2	Excalibur_c29255_366	4B	104	8.12E-05	8.91
Zn	E1	BS00012036_51	2B	108	8.49E-05	8
	E1	BS00062691_51	4B	62	7.05E-06	12.12
	E1	CAP8_rep_c6942_227	7A	148	8.98E-05	7.94
	E1	BS00072941_51	7B	71	2.42E-05	9.29
	E1	BobWhite_c7907_657	7B	71	2.42E-05	9.29
	E1	Kukri_c78330_327	7B	71	3.23E-05	8.99
	E1	RFL_Contig2540_306	7B	71	2.42E-05	9.29
	E1	TA003961-0636	7B	71	2.42E-05	9.29
	E1	BS00095819_51	7B	72	6.62E-05	8.25
	E1	Tdurum_contig75931_1967	7B	72	6.62E-05	8.25
	E1	BobWhite_c40042_842	7B	101	8.98E-05	7.94
	E3	Ra_c19225_591	2B	130	4.19E-05	8.19
	E3	wsnp_Ex_c9428_15641609	7A	159	3.63E-05	8.42
	E3	wsnp_Ex_c9428_15641639	7A	159	1.91E-05	8.98
	E4	Excalibur_c41752_392	3B	67	7.87E-07	13.25
	E4	BS00057451_51	3B	67	7.87E-07	13.25
Se	E1	Excalibur_rep_c93332_58	3D	107	1.07E-05	10.06
	E1	BobWhite_c9622_723	3D	113	3.93E-05	8.99
	E1	wsnp_Ku_c21275_31007309	5A	83	3.46E-05	8.77
	E2	Tdurum_contig4974_355	4B	61	8.65E-05	8.7
	E2	wsnp_Ex_c14654_22713386	7A	42	2.66E-05	9.68
	E2	D_contig06359_118	7D	56	2.80E-05	9.52

E1, E2, E3 and E4 were same as the Table 1

For Mn content of the wheat grain, a total of 66 MTAs loci (*P* < 10^–4^) were identified on chromosomes 1B, 2A, 2B, 3A, 4A, 4B, 5A, 5B, and 7B in three environments (Table S3). Three MTAs clusters were found on chromosomes 1B, 5A, and 7B. Of these, 14 MTAs loci at a genetic position of 75 cM of chromosome 1B were found at *P* < 10^–5^ levels in E2 (Table 3).

In turn, 77 MTAs loci (*P* < 10^–4^) were identified to be associated with Fe content on 13 chromosomes (1A, 1D, 3A, 4A, 4B, 4D, 5A, 5B, 6B, 7A, 7B and 7D) in the four experimental environments (Table S3). MTAs clusters were detected on chromosomes 5A, 6B, 7B, and 7D. Among them, 20 MTAs loci were found at the *P* < 10^–5^ level in E2, E3, and E4, explaining 8.07% to 16.23% of the phenotypic variation (Table 3). Ten MTAs loci were concentrated on the genetic position 26 cM–27 cM on chromosome 7D. Five MTAs loci explained more than 10% of the phenotypic variation on chromosomes 6B and 7B. The Excalibur_c19455_3496 locus explained the maximum phenotypic variation observed (16.23%) on chromosome 7B.

With respect to Cu, 96 MTAs loci (*P* < 10^–4^) controlling Cu content in wheat grain were detected on 15 chromosomes in the four experimental environments (Table S3). Some MTAs clusters were found on chromosomes 1A, 1 B, and 5A. However, at *P* < 10^–5^, there was only one locus, Excalibur_c29255_366, on chromosome 4 B (Table 3).

As for Zn content in wheat grain, there were 95 loci (*P* < 10^–4^) identified on 17 chromosomes, except for chromosomes 2A, 2D, 6A, and 6D in the four experimental environments (Table S3). MTAs clusters were found mainly on 2B, 3D, 5B, 6B, and 7B; 16 MTAs loci were found at the *P* < 10^–5^ level in E1, E3, and E4 (Table 3). These three explained more than 10% of the phenotypic variation on chromosomes 3B and 4B. The BS00057451_51 locus exhibited the maximum (13.25%) phenotypic variation observed.

With respect to Se, 57 MTAs loci (*P* < 10^–4^) controlling Se content in wheat grain were identified on chromosomes 1A, 2 B, 3A, 3D, 4A, 4 B, 5A, 6 B, 7A, 7 B, and 7D in the four experimental environments (Table S4). Some MTAs clusters were found on chromosomes 5A, 6 B, and 7 B, and six MATs loci were detected at the *P* < 10^–5^ level (Table 4), but only one major MAT with 10.06% phenotypic variation was detected on chromosome 3D.

**Table 4:** Genome-wide Association Mapping results of heavy metal elements (*P* < 10^–5^)

Trait	Env	SNP marker	Chr.	Position	*P*	*R*^2^ (%)
Cd	E3	Tdurum_contig44851_927	1B	162	3.23E-06	11.77
	E2	BS00083531_51	1D	72	6.82E-05	8.51
	E3	Tdurum_contig44851_593	1D	164	3.23E-06	11.77
	E4	RAC875_c9594_1289	1D	65	9.63E-05	8.20
	E4	wsnp_Ex_rep_c108004_91402649	2A	168	4.77E-05	8.87
	E4	GENE-0762_808	2A	168	4.64E-05	8.90
	E2	CAP12_rep_c6956_169	2B	115	7.91E-05	8.32
	E2	wsnp_Ex_c17538_26261053	2B	100	8.63E-05	8.25
	E2	RAC875_c59545_122	2B	104	7.26E-05	8.41
	E2	Excalibur_rep_c66577_159	2B	107	5.00E-05	8.82
	E3	Tdurum_contig13489_292	4A	75	3.65E-06	11.65
	E3	Kukri_c59197_207	4B	6	7.70E-06	11.05
	E3	RAC875_c9572_588	4B	63	8.79E-05	8.22
	E4	wsnp_Ex_c28908_37989067	5A	27	4.85E-06	11.34
	E4	wsnp_Ku_c1254_2498515	5A	27	1.43E-05	10.16
	E4	BS00033185_51	5B	174	9.69E-05	8.14
	E3	RAC875_rep_c69613_547	5D	56	6.85E-05	9.10
	E3	wsnp_Ex_c7713_13153321	6B	92	4.09E-05	9.03
	E3	Excalibur_c7713_272	7A	92	8.57E-05	8.24
	E4	Excalibur_c11062_582	7B	171	5.52E-05	8.72
	E4	Excalibur_c25090_830	7B	171	2.68E-05	9.49
	E4	RAC875_rep_c110526_229	7B	171	5.52E-05	8.72
Pb	E3	Ex_c4206_502	1B	108	6.88E-05	8.48
	E2	BS00083626_51	2B	173	9.90E-05	7.94
	E4	BS00022424_51	3A	141	5.65E-05	8.76
	E3	Tdurum_contig48760_112	5A	69	7.77E-05	8.37
	E4	wsnp_Ex_c28908_37989067	5A	27	1.10E-05	10.53
	E4	wsnp_Ku_c1254_2498515	5A	27	3.89E-05	9.16
	E1	Excalibur_c16961_85	6B	64	2.15E-05	9.44
	E1	BobWhite_c27318_380	6B	67	6.06E-05	8.35
	E1	Excalibur_c6416_1712	6B	67	6.07E-05	8.35
	E1	BobWhite_c36415_378	6B	67	9.21E-05	7.90
	E1	IACX203	6B	67	3.00E-05	9.12
	E3	Excalibur_c1215_334	7A	127	5.29E-05	8.88
	E4	RAC875_c57326_85	7B	134	8.71E-05	8.30
	E4	wsnp_Ex_c8400_14157060	7B	134	8.71E-05	8.30
	E4	Excalibur_c19455_3496	7B	163	9.11E-06	10.81
	E4	Excalibur_c11062_582	7B	171	1.90E-06	12.47
	E4	Excalibur_c25090_830	7B	171	8.11E-07	13.43
	E4	RAC875_rep_c110526_229	7B	171	1.90E-06	12.47

E1, E2, E3 and E4 were same as the Table 1

### MTAs of heavy metal elements

For the As content in wheat grain, 67 MTAs loci (*P* < 10^–4^) were identified on 14 chromosomes (1A, 1B, 2A, 2B, 3B, 3D, 4A, 5A, 5B, 6A, 6B, 7A, 7B and 7D) in the four experimental environments (Table S4). Some MATs clusters were found on chromosomes 1 1B, 2B, 6B, 7A, and 7B. However, at the *P* < 10^–5^ level, no loci were found.

In turn, there were 159 MTAs loci (*P* < 10^–4^) associated with Cd content in wheat grain on 19 chromosomes, except for chromosomes 2D and 7D in the four experimental environments (Table S4). There were MTAs clusters on chromosomes 1B, 1D, 2B, 3B, and 5B; further, 22 loci were found at the *P* < 10^–5^ level in E2, E3, and E4 (Table 4). Six loci explained more than 10% of phenotypic variation on chromosomes 1B, 1D, 4A, 4B, and 5A.

Finally, 99 MTAs (*P* < 10^–4^) associated with Pb content were found on 16 chromosomes (1A, 1B, 1D, 2A, 2B, 3A, 3B, 4A, 4B, 5A, 5B, 6A, 6B, 7A, 7B, and 7D in the four experimental environments (Table S4). MTAs clusters were detected mainly on chromosomes 1B, 3B, 5B, 6B, and 7B. Of these, 18 loci were identified at the *P* < 10^–5^ level, which involved chromosomes 1B, 2B, 3A, 5A, 6B, 7A, and 7B (Table 4). Five loci explained more than 10% of the phenotypic variation on chromosomes 5A and 7B in E4. The maximum contribution rate of Excalibur_c25090_830 on chromosome 7B was 13.43%, and its P-value was as low as 8.11 × 10^–7^.

### Stable MTAs loci at the Tai’an locations over 3 years

A total of 66 stable MTAs loci (*P* < 10^–4^) were found for Ca, Mn, Cu, Zn, Se, Cd, and Pb content in two or more of the tested environments (Table 5). Of these, five loci were detected for Ca content on chromosome 5A, and two major MATs explained more than 10% of the phenotypic variation. Eighteen stable MTAs for Mn content were identified on chromosomes 7A and 7B. Most were concentrated at the genetic position 75 cM on chromosome 7B. A stable locus was found for Cu and Zn content on chromosomes 1A and 3B, and the BS00057451_51 locus on chromosome 3B explained 13.25% of the phenotypic variation. Two stable loci on chromosomes 4 B and 7A were detected for Se content. The residue of stable MTAs loci was identified for Cd and Pb content. Five major loci were identified for Pb content.

**Table 5:** Stable marker loci at Taian location of different years (*P* < 10^–4^)

Trait	SNP marker	Chr.	Position	*P*	*R*^2^ (%)
Ca	Excalibur_c15014_1170	5A	50	3.71E-04	6.76
	GENE-3167_70	5A	50	3.71E-04	6.76
	Kukri_c2781_719	5A	50	3.71E-04	6.76
	Kukri_c41797_393	5A	53	7.13E-06	10.97
	RFL_Contig2187_1025	5A	53	7.13E-06	10.97
Mn	BobWhite_rep_c66032_270	1B	71	2.38E-04	6.99
	wsnp_BE443332B_Ta_2_2	1B	71	2.21E-04	7.05
	wsnp_BE443930B_Ta_2_2	1B	71	2.82E-04	6.81
	BS00022619_51	1B	75	7.72E-05	8.14
	BS00022920_51	1B	75	6.72E-05	8.28
	Excalibur_c5218_75	1B	75	7.28E-05	8.20
	IAAV2125	1B	75	6.72E-05	8.28
	IAAV6731	1B	75	6.72E-05	8.28
	IAAV9005	1B	75	1.12E-04	7.74
	JD_c3116_778	1B	75	6.26E-05	8.33
	Ra_c37969_549	1B	75	8.25E-05	8.05
	RAC875_c19014_725	1B	75	6.26E-05	8.33
	RAC875_rep_c112555_200	1B	75	6.72E-05	8.28
	RAC875_rep_c119728_146	1B	75	8.92E-05	7.97
	TA003725-0553	1B	75	8.92E-05	7.97
	Tdurum_contig81102_102	1B	75	8.92E-05	7.97
	wsnp_BF478690B_Ta_2_1	1B	75	6.72E-05	8.28
	wsnp_Ex_c4561_8184576	1B	75	6.26E-05	8.33
Cu	RAC875_c23158_301	1A	77	6.31E-04	6.28
Zn	BS00057451_51	3B	67	7.87E-07	13.25
Se	Tdurum_contig4974_355	4B	61	8.65E-05	8.70
	wsnp_Ex_c14654_22713386	7A	42	2.66E-05	9.68
Cd	Kukri_c105601_74	1B	51	2.15E-04	7.29
	RAC875_c25101_644	1B	51	2.15E-04	7.29
	BS00022255_51	1B	57	1.50E-04	7.67
	Kukri_c18006_1568	1B	57	2.15E-04	7.29
	RAC875_c8271_1352	1B	57	2.15E-04	7.29
	RAC875_c8271_1469	1B	57	2.15E-04	7.29
	RAC875_c8271_887	1B	57	2.15E-04	7.29
	RAC875_rep_c96733_369	1B	57	2.15E-04	7.29
	wsnp_Ku_c11987_19472688	1B	57	2.15E-04	7.29
	wsnp_Ku_c11987_19473636	1B	58	2.06E-04	7.35
	D_contig25392_201	1B	61	1.50E-04	7.67
	Kukri_c23300_267	1B	61	2.15E-04	7.29
	RAC875_c64253_435	5A	83	8.35E-04	5.92
Pb	BS00022255_51	1B	57	1.28E-04	7.90
	D_contig25392_201	1B	61	1.28E-04	7.90
	RAC875_c9594_1289	1D	65	5.83E-04	6.37
	BS00091763_51	2A	167	5.30E-04	6.41
	GENE-0762_808	2A	168	1.51E-04	7.72
	wsnp_Ex_rep_c108004_91402649	2A	168	2.24E-04	7.31
	wsnp_Ex_c14162_22093694	2B	85	3.44E-04	7.49
	BS00022424_51	3A	141	5.65E-05	8.76
	RAC875_c17479_359	3A	93	8.02E-04	5.99
	Tdurum_contig61465_781	4B	61	2.53E-04	7.18
	wsnp_Ex_c28908_37989067	5A	27	1.10E-05	10.53
	wsnp_Ku_c1254_2498515	5A	27	3.89E-05	9.16
	BobWhite_c46416_247	5B	183	3.24E-04	6.92
	BS00067074_51	5B	183	1.29E-04	7.88
	CAP7_c8713_356	5B	183	1.29E-04	7.88
	Kukri_c4594_825	5B	183	3.24E-04	6.92
	RAC875_c1035_65	5B	183	1.29E-04	7.88
	Tdurum_contig60189_310	5B	183	1.30E-04	7.89
	BS00068775_51	5B	184	1.65E-04	7.72
	CAP7_c3697_87	6B	86	1.31E-04	7.87
	Tdurum_contig68217_361	6B	86	1.31E-04	7.87
	BobWhite_c17095_237	7A	136	3.65E-04	6.80
	Excalibur_c19455_3496	7B	163	9.11E-06	10.81
	Excalibur_c11062_582	7B	171	1.90E-06	12.47
	Excalibur_c25090_830	7B	171	8.11E-07	13.43
	RAC875_rep_c110526_229	7B	171	1.90E-06	12.47

### Multi-effect MTAs loci of mineral elements

There were eight multi-effect MTAs loci for controlling more than one trait, mainly distributed on chromosomes 3B, 7B, and 5A (Table 6). There were two loci, BS00057451_51 and Excalibur_c41752_392, concurrently associated with Ca and Zn content on chromosome 3B. These two loci contributed to 13.25% of the variation in Zn content. One locus, Excalibur_c11062_582, simultaneously controlled Fe and Cd content on chromosome 7B, but the contribution to Fe content of this locus exhibited more than 10%. Two loci, RAC875_rep_c110526_229 and Excalibur_c25090_830, were simultaneously associated with Fe, Cd, and Pb content on chromosome 7B. These two loci explained more than 10% of the variation in Fe and Pb content. The three loci were concentrated on the genetic position 171 cM of chromosome 7B. There was one locus, Excalibur_c19455_3496, concurrently controlling Fe and Pb content, which accounted for more than 10% of the phenotypic variation on chromosome 7B. Two loci were identified for Cd and Pb content simultaneously on chromosome 5A, accounting for more than 10% phenotypic variation.

**Table 6:** Multi-effect loci associated with mineral elements (*P* < 10^–5^)

Trait	SNP marker	Chr.	Position	*P*	*R*^2^ (%)
Ca	BS00057451_51	3B	67	4.70E-05	8.92
Zn	BS00057451_51	3B	67	7.87E-07	13.25
Ca	Excalibur_c41752_392	3B	67	4.70E-05	8.92
Zn	Excalibur_c41752_392	3B	67	7.87E-07	13.25
Fe	Excalibur_c11062_582	7B	171	1.44E-05	10.26
Cd	Excalibur_c11062_582	7B	171	5.52E-05	8.72
Fe	RAC875_rep_c110526_229	7B	171	1.44E-05	10.26
Cd	RAC875_rep_c110526_229	7B	171	5.52E-05	8.72
Pb	RAC875_rep_c110526_229	7B	171	1.90E-06	12.47
Fe	Excalibur_c25090_830	7B	171	2.81E-06	12.07
Cd	Excalibur_c25090_830	7B	171	2.68E-05	9.49
Pb	Excalibur_c25090_830	7B	171	8.11E-07	13.43
Fe	Excalibur_c19455_3496	7B	163	8.12E-08	16.23
Pb	Excalibur_c19455_3496	7B	163	9.11E-06	10.81
Cd	wsnp_Ex_c28908_37989067	5A	27	4.85E-06	11.34
Pb	wsnp_Ex_c28908_37989067	5A	27	1.10E-05	10.53
Cd	wsnp_Ku_c1254_2498515	5A	27	1.43E-05	10.16
Pb	wsnp_Ku_c1254_2498515	5A	27	3.89E-05	9.16

#### Identification of stable MTAs and its alleles analysis in wheat accessions

**Fig. 1:**
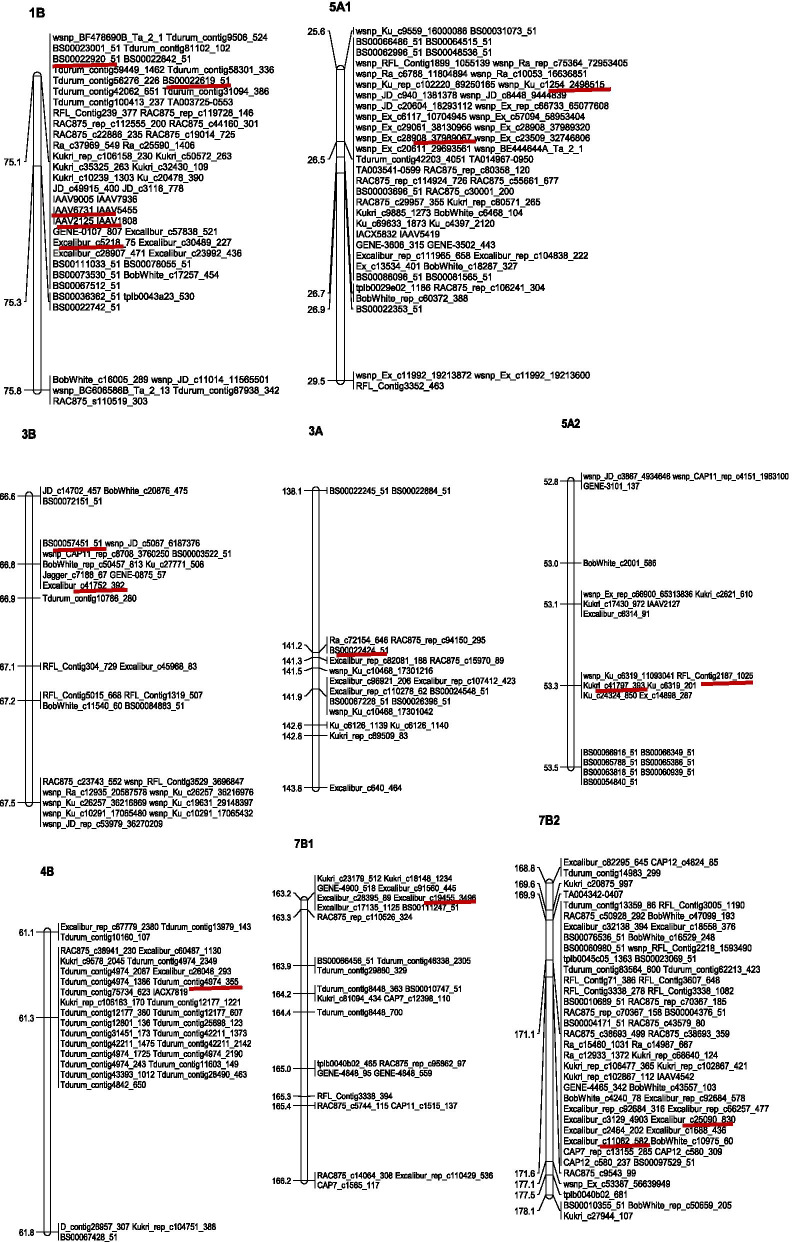
The position of major stable MTAs in the chromosome maps

By screening the results (Tables S3 and S4), there were thirteen SNP markers identified for stable MTAs associated with Ca, Mn, Zn, Se and Pb content (Table 7 and Fig. 1). Of which, the phenotypic value of Ca content associated with Kukri_c41797_393- TT on chromosome 5A was significantly higher than that associated with Kukri_c41797_393- CC across all four environments, which indicated that the contribution of Kukri_c41797_393-TT locus to Ca content was better than that of Kukri_c41797_393- CC locus, so did the contribution of RFL_Contig2187_1025 locus to Ca content.

**Table 7:** The allele analysis of stable MTAs in different mineral elements

Trait	SNP marker	Chr.	Position	Allele	Mean (μg/mL)	
Ca	Kukri_c41797_393	5A	53	T/C	CC	1807.8624 b
					TT	2084.2116 a
Ca	RFL_Contig2187_1025	5A	53	T/C	CC	1807.8624 b
					TT	2084.2116 a
Mn	Excalibur_c5218_75	1B	75	A/G	AA	110.2797 b
					GG	118.0122 a
Mn	IAAV2125	1B	75	A/C	AA	110.2636 b
					CC	117.9426 a
Mn	IAAV6731	1B	75	A/C	AA	110.2636 b
					CC	117.9426 a
Mn	BS00022619_51	1B	75	A/G	AA	118.0122 a
					GG	110.1032 b
Mn	BS00022920_51	1B	75	A/G	AA	117.9426 a
					GG	110.2636 b
Zn	BS00057451_51	3B	67	A/G	AA	93.5820 b
					GG	107.1229 a
Se	Tdurum_contig4974_355	4B	61	T/C	CC	0.0003 b
					TT	0.0007 a
Se	wsnp_Ex_c14654_22713386	7A	42	T/C	CC	0.0007 a
					TT	0.0003 b
Pb	Excalibur_c19455_3496	7B	163	A/G	AA	0.0105 a
					GG	0.0003 b
Pb	Excalibur_c25090_830	7B	171	T/C	TT	0.0113 a
					CC	0.0003 b
Pb	Excalibur_c11062_582	7B	171	A/C	AA	0.1469 a
					CC	0.0557 b

For Mn content, five SNP makers were detected on chromosome 1B (Table 7). The contribution of AA allele to Mn content was significantly higher than that of GG allele for BS00022619_51 and BS00022920_51 markers, but for Excalibur_c5218_75 marker, the GG allele was better than the AA allele for improving the Mn content. The contribution of CC allele of IAAV2125 and IAAV6731 to Mn content showed better than that of AA allele.

Only one stable marker BS00057451_51 was found on chromosome 3B for Zn content, and the contribution of GG allele was significantly better than that of AA allele (Table 7 and Fig. 1).

For Se content, two markers, Tdurum_contig4974_355 and wsnp_Ex_c14654_22713386, were found on chromosome 4B and 7A, respectively (Table 7 and Fig. 1). The contribution of Tdurum_contig4974_355-TT allele showed significantly better than that of Tdurum_contig4974_355-CC for Se content, but for wsnp_Ex_c14654_22713386 marker, the CC allele was better than the TT allele.

Three stable SNP markers were identified on chromosome 7B for heavy metal Pb content (Table 7 and Fig. 1). The contributions of the alleles GG, CC and CC showed better than that of the alleles AA, TT and AA for Excalibur_c19455_3496, Excalibur_c25090_830 and Excalibur_c11062_582 to reduce the Pb content, respectively. Most interestingly, these three SNP markers were also associated with Fe content.

So a pyramid analysis of the alleles of these different stable SNP markers were further studied in wheat accessions, seven accessions were found with high beneficial-mineral-element contents and low heavy-metal-element contents (Table 8). Of which, five accessions (B111, B117, B148, B46 and B67) contained more than one beneficial mineral elements with high content without Pb content. These results provided good wheat resources and elite alleles with beneficial mineral elements in wheat breeding.

**Table 8:** A pyramid analysis of different QTL alleles in certain wheat accessions

Wheat accessions	Mineral elements	SNP marker	Allele	Content (ug/mL)
B111	Ca	Kukri_c41797_393	TT	2903.4414
	Ca	RFL_Contig2187_1025	TT	
	Zn	BS00057451_51	GG	139.8417
	Pb	Excalibur_c19455_3496	GG	0
	Pb	Excalibur_c11062_582	CC	
B117	Ca	Kukri_c41797_393	TT	2356.8505
	Ca	RFL_Contig2187_1025	TT	
	Fe	Excalibur_c19455_3496	AA	185.3263
	Pb	Excalibur_c11062_582	CC	0
B148	Mn	Excalibur_c5218_75	GG	148.6161
	Mn	IAAV2125	CC	
	Mn	IAAV6731	CC	
	Mn	BS00022619_51	AA	
	Mn	BS00022920_51	AA	
	Zn	BS00057451_51	GG	100.9967
	Pb	Excalibur_c19455_3496	GG	
	Pb	Excalibur_c11062_582	CC	0
B46	Mn	Excalibur_c5218_75	GG	140.9914
	Mn	IAAV2125	CC	
	Mn	IAAV6731	CC	
	Mn	BS00022619_51	AA	
	Mn	BS00022920_51	AA	
	Zn	BS00057451_51	GG	107.564
	Se	Tdurum_contig4974_355	TT	0.0016
	Se	wsnp_Ex_c14654_22713386	CC	
	Pb	Excalibur_c19455_3496	GG	0
	Pb	Excalibur_c11062_582	CC	
B67	Fe	Excalibur_c19455_3496	AA	364.4478
	Fe	Excalibur_c25090_830	CC	
	Fe	Excalibur_c11062_582	AA	
	Se	Tdurum_contig4974_355	TT	0.0016
	Se	wsnp_Ex_c14654_22713386	CC	
	Pb	Excalibur_c25090_830	CC	0
B70	Fe	Excalibur_c25090_830	TT	358.5477
	Se	Tdurum_contig4974_355	TT	0.0019
	Se	wsnp_Ex_c14654_22713386	CC	0
	Pb	Excalibur_c19455_3496	GG	
	Pb	Excalibur_c11062_582	CC	
B114	Mn	Excalibur_c5218_75	GG	156.3802
	Mn	IAAV2125	CC	
	Mn	IAAV6731	CC	
	Mn	BS00022619_51	AA	
	Mn	BS00022920_51	AA	
	Pb	Excalibur_c19455_3496	GG	0
	Pb	Excalibur_c11062_582	CC	

### Prediction of candidate genes for important loci for mineral elements

In all, 16 new candidate genes were predicted for 17 important loci for mineral elements (Table S5). On chromosome 3B, two candidate genes, *TraesCS3B02G307600.1* and *TraesCS3B02G307400*, were found for BS00057451_51 and Excalibur_c41752_392, respectively, which involved Zn and Ca content. Their functions were primarily involved in metal ion binding, calcium ion binding, ATP binding, ATPase activity, DNA binding, RNA binding, and protein kinase activity.

There was one candidate gene for Se content predicted on chromosome 3D, *TraesCS3D02G201900*, whose function is modification-dependent protein binding in wheat. Furthermore, metal ion binding and calcium ion binding of this gene were found in Arabidopsis and rice, respectively.

Three candidate genes, *TraesCS5A02G256700.1*, *TraesCS5A01G256800.1*, and *TraesCS5A02G257000.1*, were identified on chromosome 5A for Ca content. The gene *TraesCS5A02G256700.1* for Kukri_c41797_393 had ribosomal small subunit biogenesis and exportation from the nucleus in wheat, but metal ion binding, Ca ion binding, and Ca transmembrane activity in Arabidopsis. The *TraesCS5A02G257000.1* gene functions in Zn-ion binding, Zn-ion transmembrane transporter activity, and metal ion binding in Arabidopsis and rice.

For Zn content, there was one candidate gene *TraesCS7B02G142200* for BobWhite_c7907_657 was identified on chromosome 7B, whose function was related to DNA binding and metal ion binding in wheat.

For Fe content, two candidate genes, *TraesCS6B02G029300* and *TraesCS6B02G029200*, for Excalibur_c6326_77 were identified on chromosome 6B. The functions of these two genes are mainly related to Ca-dependent Ca-ion binding, Fe-ion binding, ATP binding, and DNA binding in *Arabidopsis*.

On chromosome 7B, two candidate genes were identified for Fe content which were also involved in Cd and Pb content. The gene *TraesCS7B02G480300* for Excalibur_c19455_3496 was mainly related to Fe-ion binding, Fe- and Zn-ion transmembrane transporter activity, Ca-ion binding, metal ion binding, and metal-ion transmembrane transporter activity in *Hordeum vulgare*, *Oryza sativa*, and *Arabidopsis*. This gene mainly participates in the biological processes of Fe, Zn, and metal ion transport. The other gene, *TraesCS7B02G478200*, was also mainly related to Fe-ion binding, metal ion binding, Zn-ion binding, and Ca-ion binding in *Oryza*, *Arabidopsis*, and *Triticum urartu*. In *Arabidopsis*, this gene shows a biological response to Cd ions as an aspartic-type endopeptidase activity.

Five candidate genes were found for Cd content on chromosomes 1B, 1D, 4A, 4B, and 5A. The gene *TraesCS1B02G474800* for Tdurum_contig44851_927 was mainly related to Cu-ion binding and metal ion binding in *Oryza* and *Arabidopsis*. The gene mainly participates in lignin breakdown. The function of *TraesCS1D02G448700* mainly involves Fe-ion binding, Ca-ion binding, metal ion binding, and Mg-ion binding in *Arabidopsis* and *Oryza*. One of the biological processes it influences is the response to Cd ions. The gene *TraesCS4B02G004800* functions in Cu-ion binding, metal ion binding, four Fe and four S cluster binding, and the management of ion transmembrane transporter activity in *Oryza* and *Arabidopsis*. However, the gene *TraesCS5A02G014600* affected Cd and Pb content involved in Zn-ion binding, and cation- and Mn-transmembrane transporter activity, Mg-ion binding, and metal ion binding.

## Discussion

Metal elements include beneficial and harmful elements; of which, beneficial elements are important for maintaining good human health. However, to ensure food safety, heavy metals should be avoided. In addition to the agronomic practices for biofortification of Zn, Fe, and Se, genetic improvement of these elements has become important. Previous studies have identified some QTL/gene loci for Zn, Se, and Fe content involving all 21 wheat chromosomes [13, 15–18, 27, 31–38]; however, in our study, MTAs loci for Ca, Zn, Fe, Se, Mn, and Cu content were found on 20 chromosomes, i.e. all except for chromosome 6D. Previously, the important QTLs detected for Zn, Fe, and Se were located on chromosomes 7B, 4A, 3B, 2D, 5A, 5B, 6A, and 7A [13]. In this study, chromosomes 1B, 3B, 3D, 4B, 5A, 6B, 7B, and 7D were important for MTAs loci associated with Ca, Zn, Fe, Se, Mn, and Cu content. Comparing these, common chromosomes 3B, 5A, and 7B were found to play important roles in regulating Ca, Fe, and Zn concentrations in the wheat grain, and to contain some important genes.

Using bioinformatics, the candidate genes (mRNA_2.1, mRNA_3.1, mRNA_10.1, mRNA_23.1, mRNA_24.1, and mRNA_32.1; mRNA_11.1, mRNA_34.1, mRNA_42.1, and mRNA_44.1) associated with Zn content were identified in the physical regions of 3BS (723,504,241 to 723,611,488) (first six genes) and 5AL (462,763,758 to 468,582,184) (last four genes) [21]. This study identified two candidate genes, *TraesCS3B02G307600.1*, with the physical region from 493,655,348 to 493,657,938 and *TraesCS3B02G307400* with the physical region from 493,648,449 to 493,653,177, on chromosome 3B, which is associated with grain Ca and Zn concentrations. By comparing their physical positions, we found that these two genes are different from the above genes located upstream of previously published genes. These published 3BS genes were found to belong to the mitogen-activated protein kinase (MAPK) family of genes involved in kinase activity, leading to protein phosphorylation, which in turn assists in the desired molecular function in various biological processes [13]. MAPKs are involved in Zn uptake and transport through signalling pathways. The mRNA_32.1 gene encodes a suppressor of the white apricot protein associated with Zn concentration in chickpea seeds [39], an RNA-binding protein involved in RNA processing [13]. In our study, according to the putative functions of these two new candidate genes, they are seemingly also involved in protein kinase activity and RNA binding; although primarily they are involved in metal ion binding, Ca-ion binding, ATP binding, and ATPase activity, indicating that they are involved in Ca and Zn uptake and transport. On chromosome 5AL, the last four genes encode TaMTP proteins, which are directly or indirectly involved in Zn biofortification, and their functions are mainly involved in DNA binding, Zn/Fe binding, and protein dimerisation [13, 21], However, this study identified three candidate genes, *TraesCS5A02G256700.1*, *TraesCS5A01G256800.1*, and *TraesCS5A02G257000.1* on chromosome 5A for Ca content in wheat grain; further, their physical region is from 472,274,579 to 472,347,557, which is different from the physical position of the published genes. Their functions are primarily involved in Ca-ion binding, Zn-ion binding, metal ion binding, Zn- and iron-transmembrane transporter activity, ATP binding, and protein kinase activity. Additionally, some researchers have found that chromosome 5A plays an important role in regulating grain Cu concentration [27, 34]. Therefore, these new candidate genes identified on chromosomes 3B and 5A are suitable for further molecular genetic research.

On chromosome 7B, two major QTLs for controlling Zn content were identified using DArT-seq [15]. These genes encode the kinase-like superfamily, which catalyses phosphorylation processes in which some protein structures are Zn related [15]. In this study, a new candidate gene, *TraesCS7B02G142200*, was predicted to be involved in DNA binding and metal ion binding in wheat. These results indicate that the genes are controlling Zn concentration on chromosome 7B.

Regarding ferritin, previous studies found genes involved in vacuolar iron transporters and transporter-like protein, such as TaFer1 and TaFer2 on homologous groups 5 and 4 in wheat, respectively [40]. Another wheat gene relevant to biofortification is the major grain protein gene Gpc1 on chromosome 6B, which also affects Zn and Fe concentrations in the grain [13, 41]. as it can regulate the expression of several genes involved in the export and transport of Zn and Fe into the grain through the phloem [42]. This study predicted three candidate genes, *TraesCS6B02G029300*, *TraesCS7B02G480300*, and *TraesCS7B02G478200*, with physical regions from 17,703,175 to 17,704,083, 734,259,844 to 734,274,042, and 733,527,458 to 733,530,221, respectively, which are primarily involved in Fe- and Ca-ion binding, metal ion binding, Zn-ion binding, ATP binding, ATPase activity, and DNA binding. Thus, these genes are important for improving Fe concentration, a finding that warrants further research.

The accumulation of heavy metals (e.g. Cd, As, and Pb) is a complex quantitative trait controlled by multiple genes. Most previous studies on the mechanisms of Cd accumulation have focused on rice, maize, and A. thaliana. A major QTL was mapped to translate Cd from roots to shoots at the seedling stage in rice [43]. By means of GWAS, a single strong peak of SNPs associated with leaf Cd accumulation was identified in A. thaliana [44]. In maize, the genetic control of Cd accumulation in leaves was studied using genome-wide association analysis and QTL mapping, whereby candidate genes and favourable alleles were identified [4]. However, studies on the genetic control of Cd, As, and Pb in wheat are scarce at best. Here, we found some important MTAs loci for Cd and Pb content involving chromosomes 1B, 1D, 4A, 4B, 5A, 6B, and 7B. This indicates that some important genes need to be studied.

Previous studies have shown that heavy metal ATPases, metal tolerance proteins (MTPs), and natural resistance-associated macrophage proteins are involved in the deposition of metals in the grain [44]. Plant MTPs are transition metal transporters that catalyse the efflux of Zn, Fe, Mn, Cd, Co, or Ni ions from the cytoplasm to the outside of the cell or into subcellular compartments [45, 46]. Therefore, there seems to be a synergy between some heavy metals and some of the beneficial mineral elements or simply, between mineral elements. Once metal ions are absorbed in rice, translocation of Cd from the roots to the shoots requires loading of Cd into the xylem from the symplast in the stele, which in turn requires heavy metal ATPase [47]. The Cd-related gene *GRMZM2G175576* encoding a heavy metal-transporting ATPase was identified in maize, which is homologous to the rice gene *OsHMA3* [4]. However, in our study, six candidate genes associated with Cd content were predicted in wheat, two of which had multiple effects, that is, Cd and Pb content and Cd, Fe, and Pb content. Their functions are primarily involved in ion binding, including metal-, Fe-, Ca-, Cu-, Mg-, and Zn-ion binding. Therefore, there are gene interactions among mineral elements, including some that are harmful and some that are beneficial for humans.

## Conclusions

In brief, herein, 17 major MAT loci for nine mineral elements were identified, and 16 candidate genes were predicted. There were some MTA loci clusters found on 12 chromosomes (1A, 1B, 1D, 2B, 3B, 3D, 5A, 5B, 6B, 7A and 7D). Eight multi-effect MAT loci for controlling more than one trait were detected, mainly distributed on chromosomes 3B, 7B, and 5A. The functions of these candidate genes are primarily involved in ion binding, including metal-, Fe-, Ca-, Cu-, Mg-, and Zn-ion binding, ATP binding, ATPase activity, DNA binding, RNA binding, and protein kinase activity. There were gene interactions among some of the mineral elements under study. Therefore, this study provides important loci and gene information for improving mineral element content in the wheat grain. In the future, the candidate genes identified herein should be further studied to elucidate the molecular mechanisms for controlling the content of these mineral elements in the wheat grain.

## Supplementary Information

**Additional file 1: Fig. S1.** Standard curves of nine mineral elements in wheat grain. **Fig. S2.** Manhattan plot of some mineral elements. **Table S1.** Soil conditions in different planting environments. **Table S2.** ANOVA analysis of mineral elements. **Table S3.** All SNP loci significantly associated with beneficial mineral elements (*P* < 10-4). **Table S4.** All SNP loci significantly associated with heavy metal elements (*P* < 10-4). **Table S5.** Candidate genes predication and mainly functions of important MATs loci associated with mineral elements.

## Data Availability

All data used during the current study are included in this published article or are available from the corresponding author on reasonable request from https://pan.baidu.com/s/1X8ISFlrBP0X-u6kMcISGHg. Statement: the database (s) is closed, so before access to this database(s), please request the password from the corresponding author.

## Abbreviations

QTL: Quantitative trait loci
GWAS: Genome-wide association study
RILs: Recombinant inbred lines
MQTL: Meta-QTL
MTAs: Marker-trait associations
SNPs: Single nucleotide polymorphisms
DZ: Dezhou
TA: Tai’an
ANOVA: Analysis of variance
MLM: Mixed linear model
NCBI: The National Center for Biotechnology Information
MAPK: Mitogen-activated protein kinase
MTPs: Metal tolerance proteins

## References

[1] Stein AJ, Qaim M (2007). The human and economic cost of hidden hunger. Food Nutr Bull.

[2] Welch RM, Graham RD (2004). Breeding for micronutrients in staple food crops from a human nutrition perspective. J Exp Bot.

[3] Nawrot T, Plusquin M, Hogervorst J, Roels HA, Celis H, Thijs L, Vangronsveld J, Hecke EV, Staessen JA (2006). Environmental exposure to cadmium and risk of cancer: a prospective population-based study. Lancet Oncol.

[4] Zhao XW, Luo LX, Cao YH, Liu YJ, Li YH, Wu WM, Lan YZ, Jiang YW, Gao SB, Zhang ZM, Shen YO, Pan GT, Lin HJ (2018). Genome-wide association analysis and QTL mapping reveal the genetic control of cadmium accumulation in maize leaf. BMC Genomics.

[5] Navarro-Alarcon M, Lopez-Martınez MC (2000). Essentiality of selenium in the human body: relationship with different diseases. Sci Total Environ.

[6] Goldhaber SB (2003). Trace element risk assessment: essentiality vs. toxicity. Regul Toxicol Pharmacol.

[7] Fraga CG (2005). Relevance, essentiality and toxicity of trace elements in human health. Mol Aspects Med.

[8] Nielsen FH (2007). The clinical and nutritional importance of chromium—still debated after 50 years of research The nutritional biochemistry of chromium (III).

[9] Blust R (2011). Fish physiology.

[10] Broadley M, Brown P, Cakmak I, Ma JF, Rengel Z, Zhao F (2012). Beneficial elements. Marschner’s mineral nutrition of higher plants.

[11] Harding KL, Aguayo VM, Webb P (2018). Hidden hunger in South Asia: a review of recent trends and persistent challenges. Publ Health Nutr.

[12] Godecke T, Steiin AJ, Qaim M (2018). The global burden of chronic and hidden hunger: trends and determinants. Glob Food Sec.

[13] Gupta PK, Balyan HS, Sharma S, Kumar R (2021). Biofortifcation and bioavailability of Zn, Fe and Se in wheat: present status and future prospects. Theor Appl Genet.

[14] Crespo-Herrera LA, Velu G, Singh RP (2016). Quantitative trait loci mapping reveals pleiotropic efect for grain iron and zinc concentrations in wheat. Ann Appl Biol.

[15] Crespo-Herrera LA, Govindan V, Stangoulis J (2017). QTL mapping of grain Zn and Fe concentrations in two hexaploid wheat RIL populations with ample transgressive segregation. Front Plant Sci.

[16] Yang R, Wang R, Xue W (2013). QTL location and analysis of selenium content in tetraploid wheat grain. Guizhou Agric Sci.

[17] Pu ZE, Ma YU, He QY (2014). Quantitative trait loci associated with micronutrient concentrations in two recombinant inbred wheat lines. J Integr Agric.

[18] Wang P, Wang H, Liu Q (2017). QTL mapping of selenium content using a RIL population in wheat. PLoS One.

[19] Velu G, Singh RP, Crespo-Herrera L (2018). Genetic dissection of grain zinc concentration in spring wheat for mainstreaming biofortifcation in CIMMYT wheat breeding. Sci Rep.

[20] Cu ST, Guild G, Nicolson A (2020). Genetic dissection of zinc, iron, copper, manganese and phosphorus in wheat (Triticum aestivum L.) grain and rachis at two developmental stages. Plant Sci.

[21] Alomari DZ, Eggert K, von Wirén N (2018). Identifying candidate genes for enhancing grain Zn concentration in wheat. Front Plant Sci.

[22] Alomari DZ, Eggert K, von Wirén N (2019). Whole-genome association mapping and genomic prediction for iron concentration in wheat grains. Int J Mol Sci.

[23] Kumar J, Gautam S, Gahlaut V (2018). Genetics of Fe, Zn, β-carotene, GPC and yield traits in bread wheat (Triticum aestivum L.) using multi-locus and multitraits GWAS. Euphytica.

[24] Goraf YSA, Ishii T, Kim JS (2016). Genetic variation and association mapping of grain iron and zinc contents in synthetic hexaploid wheat germplasm. Plant Genet Resour.

[25] Bhatta M, Baenziger PS, Waters BM (2018). Genome-wide association study reveals novel genomic regions associated with 10 grain minerals in synthetic hexaploid wheat. Int J Mol Sci.

[26] Arora S, Cheema J, Poland J (2019). Genome-wide association mapping of grain micronutrients concentration in Aegilops tauschii. Front Plant Sci.

[27] Liu Y, Chen YR, Yang Y, Zhang QF, Fu BS, Cai J, Guo W, Shi L, Wu JZ and Chen YH. A thorough screening based on QTLs controlling zinc and copper accumulation in the grain of different wheat genotypes. Environ Sci Pollut. 2020; Research. 10.1007/s11356-020-11690-3. 10.1007/s11356-020-11690-3 33230790

[28] Uauy C, Distelfeld A, Fahima T (2006). A NAC gene regulating senescence improves grain protein, zinc, and iron content in wheat. Science.

[29] Chen GF, Zhang H, Deng ZY, Wu RG, Li DM, Wang MY, Tian JC (2016). Genome-wide association study for kernel weight-related traits using SNPs in a Chinese winter wheat population. Euphytica.

[30] Chen GF, Wu RG, Li DM, Yu HX, Deng ZY, Tian JC (2017). Genome wide association study for seedling emergence and tiller number using SNP markers in an elite winter wheat population. J Genet.

[31] Tiwari VK, Rawat N, Chhuneja P (2009). Mapping of quantitative trait loci for grain iron and zinc concentration in diploid A genome wheat. J Hered.

[32] Tiwari C, Wallwork H, Arun B (2016). Molecular mapping of quantitative trait loci for zinc, iron and protein content in the grains of hexaploid wheat. Euphytica.

[33] Peleg Z, Cakmak I, Ozturk L (2009). Quantitative trait loci conferring grain mineral nutrient concentrations in durum wheat x wild emmer wheat RIL population. Theor Appl Genet.

[34] Balint AF, Röder MS, Hell R (2007). Mapping of QTLs afecting copper tolerance and the Cu, Fe, Mn and Zn contents in the shoots of wheat seedlings. Biol Plantarum.

[35] Yasmin Z, Paltridge N, Graham R (2014). Measuring genotypic variation in wheat seed iron first requires stringent protocols to minimize soil iron contamination. Crop Sci.

[36] Hao Y, Velu G, Peña RJ (2014). Genetic loci associated with high grain zinc concentration and pleiotropic efect on kernel weight in wheat (Triticum aestivum L.). Mol Breed.

[37] Velu G, Tutus Y, Gomez-Becerra HF (2017). QTL mapping for grain zinc and iron concentrations and zinc efficiency in a tetraploid and hexaploid wheat mapping populations. Plant Soil.

[38] Liu J, Wu B, Singh RP, Velu G (2019). QTL mapping for micronutrients concentration and yield component traits in a hexaploid wheat mapping population. J Cereal Sci.

[39] Upadhyaya HD, Bajaj D, Das S (2016). Genetic dissection of seed iron and zinc concentrations in chickpea. Sci Rep.

[40] Borg S, Brinch-Pedersen H, Tauris B (2012). Wheat ferritins: improving the iron content of the wheat grain. J Cereal Sci.

[41] Tabbita F, Pearce S, Barneix AJ (2017). Breeding for increased grain protein and micronutrient content in wheat: 10 years of the GPC-B1 gene. J Cereal Sci.

[42] Pearce S, Tabbita F, Cantu D (2014). Regulation of Zn and Fe transporters by the GPC1 gene during early wheat monocarpic senescence. BMC Plant Biol.

[43] Ueno D, Kono I, Yokosho K, Ando T, Yano M, Ma JA (2009). major quantitative trait locus controlling cadmium translocation in rice (Oryza Sativa). New Phytol.

[44] Chao DY, Silva A, Baxter I, Huang YS, Nordborg M, Danku J, Lahner B, Yakubova E, Salt DE (2012). Genome-wide association studies identify heavy metal ATPase3 as the primary determinant of natural variation in leaf cadmium in Arabidopsis Thaliana. PLoS Genet.

[45] Montanini B, Blaudez D, Jeandroz S, Sanders D (2007). Chalot M Phylogenetic and functional analysis of the cation diffusion facilitator (CDF) family: improved signature and prediction of substrate specificity. BMC Genomics.

[46] Tauris B, Borg S, Gregersen PL, Holm PB (2009). A roadmap for zinc trafficking in the developing barley grain based on laser capture microdissection and gene expression profiling. J Exp Bot.

[47] Uraguchi S, Fujiwara T (2012). Cadmium transport and tolerance in rice: perspectives for reducing grain cadmium accumulation. Rice.

