# Supplementary material for: Identification of novel genomic regions associated with nine mineral elements in Chinese winter wheat grain
Source: BMC Plant Biol. 2021 Jul 1;21:311. doi: 10.1186/s12870-021-03105-3 (PMC8252321; doi:10.1186/s12870-021-03105-3)
Supplement: Supplementary file 1 — Additional file 1: Fig. S1. Standard curves of nine mineral elements in wheat grain. Fig. S2. Manhattan plot of some mineral elements. Table S1. Soil conditions in different planting environments. Table S2. ANOVA analysis of mineral elements. Table S3. All SNP loci significantly associated with beneficial mineral elements (P < 10-4). Table S4. All SNP loci significantly associated with heavy metal elements (P < 10-4). Table S5. Candidate genes predication and mainly functions of important MATs loci associated with mineral elements. [file 12870_2021_3105_MOESM1_ESM.docx]

Supplementary Figures and Tables


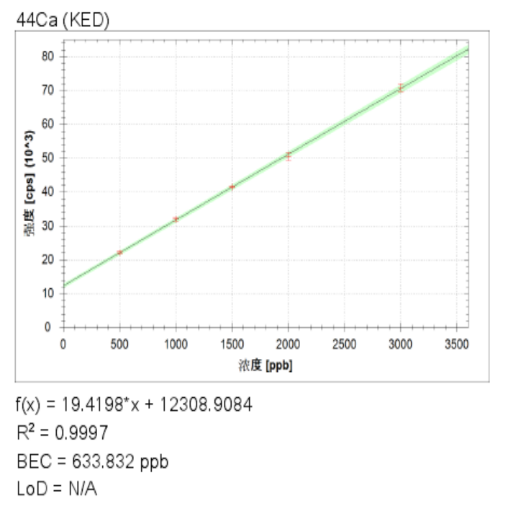

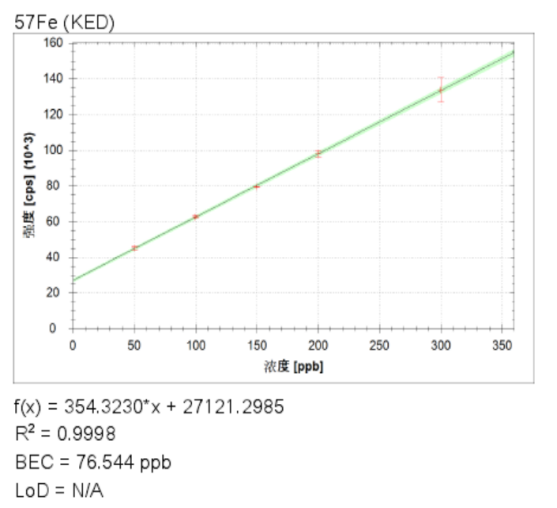

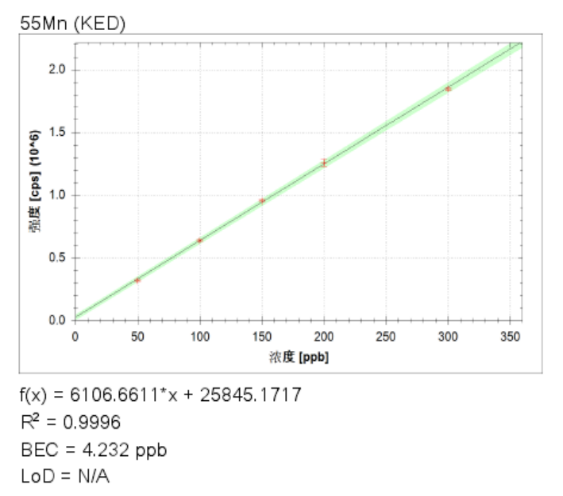

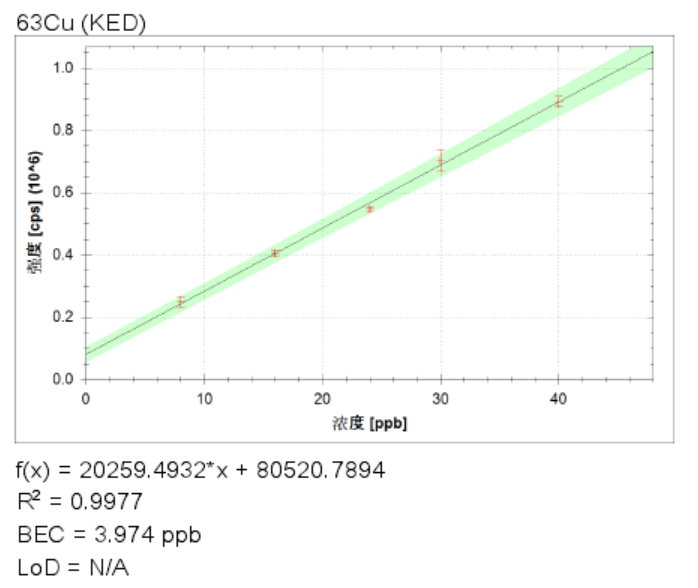


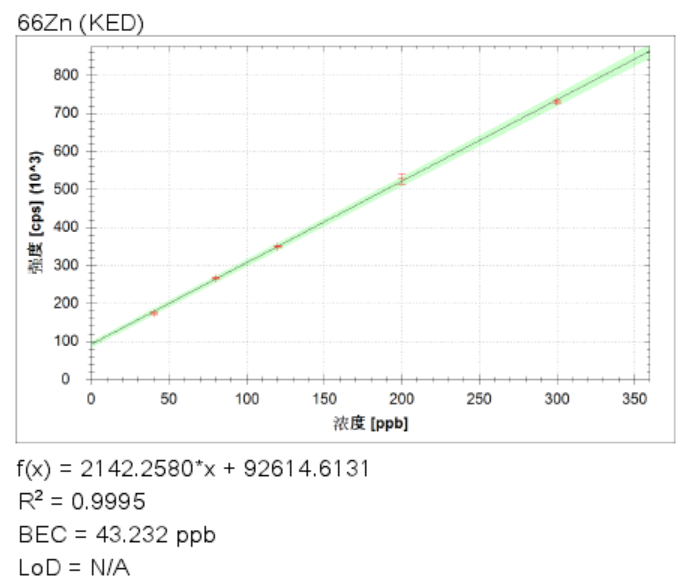

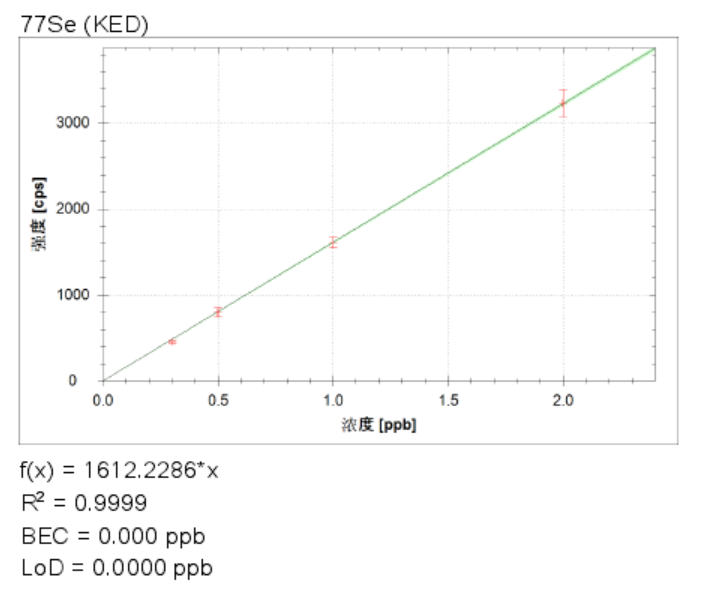


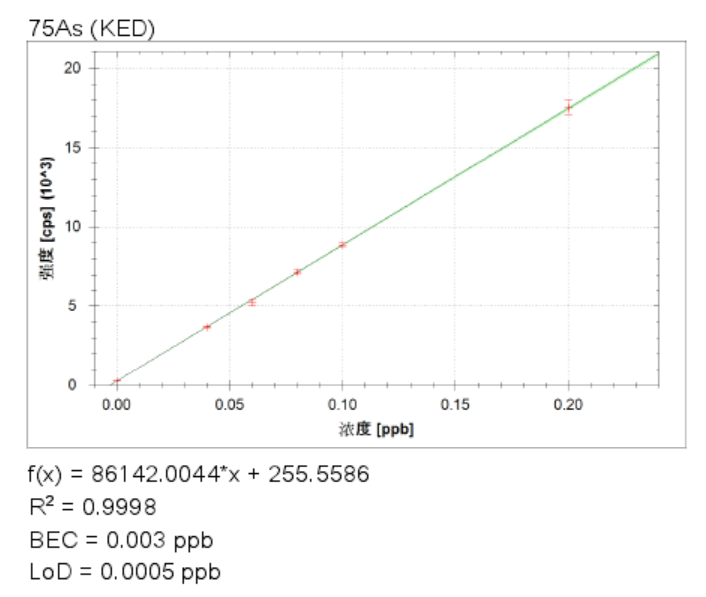

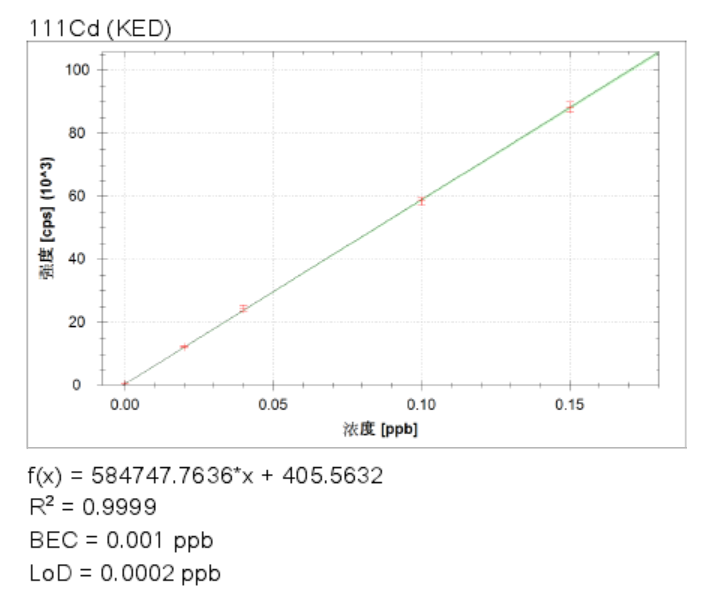

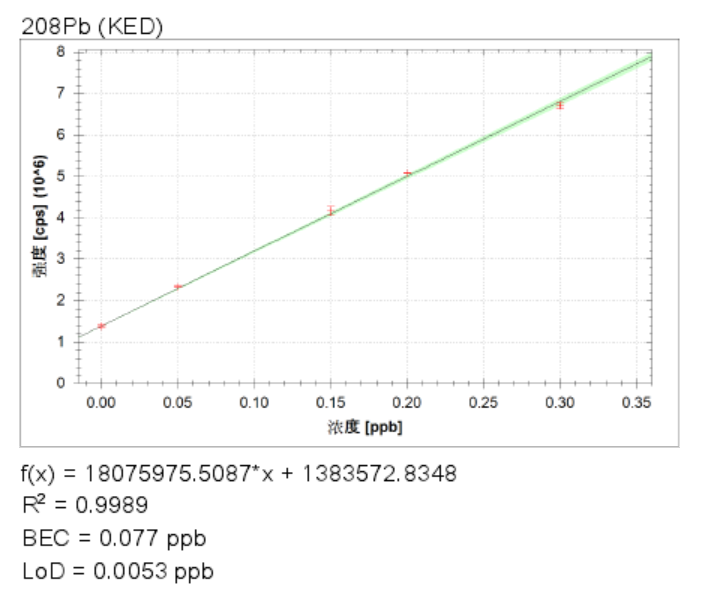


Fig. S1 Standard curves of nine mineral elements in wheat grain

a: calcium; b: manganese; c: iron; d: copper; e: zinc; f: selenium; g: arsenic; h: cadmium; i: Plumbum

Table S1 Soil conditions in different planting environments

| Location | Element | Mean (mg/kg) | SE | Location | Element | Mean (mg/kg) | SE |
| --- | --- | --- | --- | --- | --- | --- | --- |
| DeZhou | Mn | 9.39 | 0.01856 | DeZhou | As | 11.91 | 0.0393 |
| Tai'an | Mn | 10.68 | 0.02646 | Tai'an | As | 10.04 | 0.04359 |
| DeZhou | Fe | 10.86 | 0.06083 | DeZhou | Cd | 0.30 | 0.00577 |
| Tai'an | Fe | 13.56 | 0.04667 | Tai'an | Cd | 0.11 | 0.00333 |
| DeZhou | Cu | 0.94 | 0.02082 | DeZhou | Pb | 26.43 | 0.01202 |
| Tai'an | Cu | 0.83 | 0.02309 | Tai'an | Pb | 15.33 | 0.01202 |
| DeZhou | Zn | 70.28 | 0.0811 |  |  |  |  |
| Tai'an | Zn | 65.55 | 0.10214 |  |  |  |  |

Table S2 ANOVA analysis of mineral elements

| Trait |  | ANOVA analysis | |  |  |  |
| --- | --- | --- | --- | --- | --- | --- |
|  | Source | DF | Type IIISS | Mean Square | F Value | P |
| Ca | Geno | 204 | 95609928.1 | 468676.1 | 136.33 | <0.0001 |
|  | Env | 3 | 46944169.3 | 15648056.4 | 4551.81 | <0.0001 |
|  | Geno*Env | 589 | 188835425.5 | 320603.4 | 93.26 | <0.0001 |
|  | rep(Env) | 4 | 6849.8 | 1712.4 | 0.5 | 0.7371 |
|  |  |  |  |  |  |  |
| Cu | Geno | 204 | 3641.042568 | 17.848248 | 185.32 | <0.0001 |
|  | Env | 3 | 2441.358095 | 813.786032 | 8449.81 | <0.0001 |
|  | Geno*Env | 588 | 6478.852085 | 11.018456 | 114.41 | <0.0001 |
|  | rep(Env) | 4 | 0.236535 | 0.059134 | 0.61 | 0.6527 |
|  |  |  |  |  |  |  |
| Mn | Geno | 204 | 95609928.1 | 468676.1 | 136.33 | <0.0001 |
|  | Env | 3 | 46944169.3 | 15648056.4 | 4551.81 | <0.0001 |
|  | Geno*Env | 588 | 367661.3239 | 625.2744 | 94.52 | <0.0001 |
|  | rep(Env) | 4 | 15.746 | 3.9365 | 0.6 | 0.6663 |
|  |  |  |  |  |  |  |
| Fe | Geno | 204 | 8682839.34 | 42562.94 | 644.3 | <0.0001 |
|  | Env | 3 | 1225304.06 | 408434.69 | 6182.67 | <0.0001 |
|  | Geno*Env | 589 | 188835425.5 | 320603.4 | 93.26 | <0.0001 |
|  | rep(Env) | 4 | 6849.8 | 1712.4 | 0.50 | 0.7371 |
|  |  |  |  |  |  |  |
| Se | Geno | 204 | 0.00052003 | 0.00000255 | 2.66 | <0.0001 |
|  | Env | 3 | 0.00016469 | 0.00005490 | 57.25 | <0.0001 |
|  | Geno*Env | 591 | 0.00079288 | 0.00000134 | 1.40 | <0.0001 |
|  | rep(Env) | 4 | 0.00000386 | 0.00000097 | 1.01 | 0.4027 |
|  |  |  |  |  |  |  |
| Zn | Geno | 204 | 253653.7457 | 1243.4007 | 124.79 | <0.0001 |
|  | Env | 3 | 114846.0724 | 38282.0241 | 3841.94 | <0.0001 |
|  | Geno*Env | 588 | 646436.1153 | 1099.3811 | 110.33 | <0.0001 |
|  | rep(Env) | 4 | 20.6569 | 5.1642 | 0.52 | 0.7223 |
|  |  |  |  |  |  |  |
| Cd | Geno | 204 | 0.00105129 | 0.00000515 | 270.07 | <0.0001 |
|  | Env | 3 | 0.00016724 | 0.00005575 | 2921.47 | <0.0001 |
|  | Geno*Env | 591 | 0.00190033 | 0.00000322 | 168.51 | <0.0001 |
|  | rep(Env) | 4 | 0.00000010 | 0.00000003 | 1.33 | 0.2588 |


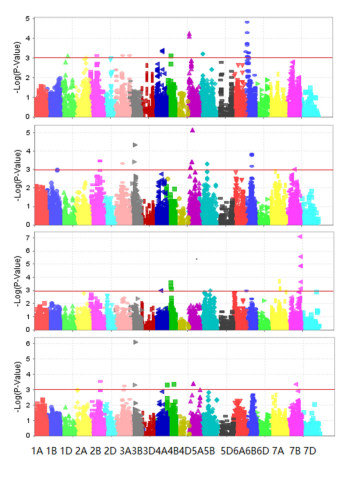

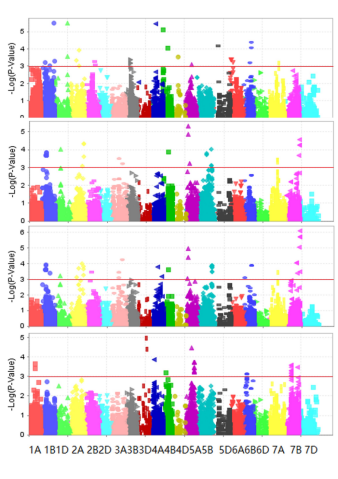


Se (E1)

Pb (E4)

Cd (E4)

Cd (E3)

Zn (E4)

Fe (E4)

Fe (E2)

Ca (E4)

Fig.S2 Manhattan plot of some mineral elements

Table S3 All SNP loci significantly associated with beneficial mineral elements (P<10^-4^)

| Trait | Env. | SNP Marker | Chr. | Site | P | R^2^(%) |
| --- | --- | --- | --- | --- | --- | --- |
| Ca | E1 | JD_c20537_742 | 6B | 114 | 8.49E-04 | 5.68 |
| Ca | E2 | TA003955-1138 | 1A | 83 | 2.68E-04 | 7.02 |
| Ca | E2 | wsnp_Ku_c5210_9289260 | 1A | 94 | 1.43E-04 | 7.72 |
| Ca | E2 | BS00039378_51 | 1A | 94 | 1.43E-04 | 7.72 |
| Ca | E2 | BS00065430_51 | 1A | 94 | 1.25E-04 | 7.81 |
| Ca | E2 | wsnp_Ex_c3258_6004611 | 1A | 95 | 6.61E-04 | 6.10 |
| Ca | E2 | wsnp_Ex_rep_c68085_66839109 | 1A | 95 | 7.08E-04 | 6.03 |
| Ca | E2 | CAP7_c1891_230 | 1A | 95 | 2.07E-04 | 7.34 |
| Ca | E2 | Kukri_c67383_102 | 1A | 95 | 7.08E-04 | 6.03 |
| Ca | E2 | RAC875_c8482_160 | 1A | 95 | 3.22E-04 | 6.88 |
| Ca | E2 | BS00062876_51 | 1A | 95 | 2.07E-04 | 7.34 |
| Ca | E2 | BS00009808_51 | 1A | 95 | 2.99E-04 | 6.96 |
| Ca | E2 | RFL_Contig4781_1792 | 1A | 95 | 7.34E-04 | 6.01 |
| Ca | E2 | CAP11_c1021_200 | 1A | 96 | 1.25E-04 | 7.81 |
| Ca | E2 | GENE-0392_97 | 1A | 96 | 1.25E-04 | 7.81 |
| Ca | E2 | BS00034278_51 | 1A | 96 | 2.85E-04 | 6.96 |
| Ca | E2 | wsnp_Ex_c6057_10611952 | 6B | 55 | 1.98E-04 | 7.33 |
| Ca | E2 | wsnp_RFL_Contig2738_2459768 | 6B | 57 | 8.08E-04 | 5.90 |
| Ca | E2 | IAAV8886 | 6B | 57 | 1.98E-04 | 7.33 |
| Ca | E2 | Ku_c30637_1294 | 6B | 57 | 1.61E-04 | 7.56 |
| Ca | E2 | Kukri_c4606_170 | 6B | 57 | 1.61E-04 | 7.56 |
| Ca | E2 | Kukri_c48571_361 | 6B | 57 | 2.65E-04 | 7.03 |
| Ca | E2 | Kukri_c75566_265 | 6B | 57 | 4.16E-04 | 6.57 |
| Ca | E2 | RAC875_c34994_183 | 6B | 57 | 2.66E-04 | 7.03 |
| Ca | E2 | Tdurum_contig43119_297 | 6B | 57 | 2.66E-04 | 7.03 |
| Ca | E2 | Excalibur_c37505_88 | 7A | 60 | 2.00E-04 | 7.92 |
| Ca | E2 | Tdurum_contig32378_439 | 7B | 81 | 4.93E-04 | 6.40 |
| Ca | E2 | RFL_Contig5480_408 | 7B | 159 | 8.18E-04 | 5.89 |
| Ca | E3 | IACX5800 | 2A | 142 | 1.96E-04 | 6.48 |
| Ca | E3 | BS00003663_51 | 2A | 142 | 1.96E-04 | 6.48 |
| Ca | E3 | Excalibur_c14201_111 | 2B | 119 | 3.15E-04 | 6.05 |
| Ca | E3 | BS00004224_51 | 2B | 119 | 3.15E-04 | 6.05 |
| Ca | E3 | Tdurum_contig82393_581 | 2B | 119 | 3.15E-04 | 6.05 |
| Ca | E3 | BS00064607_51 | 2B | 119 | 7.51E-04 | 5.29 |
| Ca | E3 | BobWhite_c13424_430 | 5D | 67 | 9.14E-04 | 5.10 |
| Ca | E3 | Tdurum_contig13048_450 | 7A | 51 | 7.58E-04 | 5.33 |
| Ca | E4 | BobWhite_c23950_145 | 2B | 138 | 3.29E-04 | 6.88 |
| Ca | E4 | Excalibur_c7051_1027 | 2B | 138 | 3.29E-04 | 6.88 |
| Ca | E4 | Kukri_c27631_1329 | 2B | 138 | 3.29E-04 | 6.88 |
| Ca | E4 | RAC875_c16064_217 | 2B | 138 | 3.29E-04 | 6.88 |
| Ca | E4 | RFL_Contig3713_280 | 2B | 138 | 3.51E-04 | 7.22 |
| Ca | E4 | RFL_Contig3713_316 | 2B | 138 | 3.26E-04 | 6.93 |
| Ca | E4 | BS00051965_51 | 2B | 139 | 3.29E-04 | 6.88 |
| Ca | E4 | RAC875_c17479_359 | 3A | 93 | 4.58E-04 | 6.54 |
| Ca | E4 | Tdurum_contig21329_326 | 3B | 60 | 3.76E-04 | 6.74 |
| Ca | E4 | Excalibur_c41752_392 | 3B | 67 | 4.70E-05 | 8.92 |
| Ca | E4 | BS00057451_51 | 3B | 67 | 4.70E-05 | 8.92 |
| Ca | E4 | Kukri_c25201_305 | 5A | 25 | 7.28E-04 | 6.07 |
| Ca | E4 | BS00023008_51 | 5A | 25 | 7.28E-04 | 6.07 |
| Ca | E4 | Excalibur_c15014_1170 | 5A | 50 | 3.71E-04 | 6.76 |
| Ca | E4 | GENE-3167_70 | 5A | 50 | 3.71E-04 | 6.76 |
| Ca | E4 | Kukri_c2781_719 | 5A | 50 | 3.71E-04 | 6.76 |
| Ca | E4 | Kukri_c41797_393 | 5A | 53 | 7.13E-06 | 10.97 |
| Ca | E4 | RFL_Contig2187_1025 | 5A | 53 | 7.13E-06 | 10.97 |
| Ca | E4 | Tdurum_contig12995_722 | 5B | 98 | 4.96E-04 | 6.46 |
| Ca | E4 | Tdurum_contig12995_792 | 5B | 98 | 4.96E-04 | 6.46 |
| Ca | E4 | Kukri_c78348_266 | 6B | 76 | 6.51E-04 | 6.18 |
| Ca | E4 | Excalibur_c32739_698 | 6B | 79 | 1.49E-04 | 7.71 |
| Ca | E4 | BobWhite_c3392_749 | 6B | 79 | 1.49E-04 | 7.71 |
| Ca | E4 | BS00010993_51 | 6B | 79 | 1.49E-04 | 7.71 |
| Ca | E4 | BS00011479_51 | 6B | 79 | 1.49E-04 | 7.71 |
| Ca | E4 | RAC875_c5129_280 | 6B | 79 | 1.66E-04 | 7.61 |
| Ca | E4 | RAC875_c60007_199 | 6B | 79 | 1.66E-04 | 7.61 |
| Ca | E4 | Kukri_rep_c103094_177 | 7B | 102 | 9.36E-04 | 6.01 |
| Mn | E1 | RAC875_c66649_186 | 1B | 101 | 7.55E-04 | 5.83 |
| Mn | E1 | BS00096498_51 | 1B | 107 | 5.98E-04 | 5.99 |
| Mn | E1 | Jagger_c7235_96 | 1B | 107 | 9.10E-04 | 5.62 |
| Mn | E1 | wsnp_Ex_c17127_25756019 | 2B | 106 | 7.80E-04 | 5.73 |
| Mn | E1 | RAC875_c6445_275 | 3A | 78 | 7.10E-04 | 5.82 |
| Mn | E1 | BS00013584_51 | 3A | 78 | 9.63E-04 | 5.56 |
| Mn | E1 | wsnp_Ex_c16295_24772702 | 5A | 111 | 9.13E-04 | 5.76 |
| Mn | E1 | wsnp_Ex_c23968_33209733 | 5A | 111 | 6.18E-04 | 5.96 |
| Mn | E1 | wsnp_Ex_c23968_33210208 | 5A | 111 | 6.18E-04 | 5.96 |
| Mn | E1 | wsnp_Ex_c23968_33210344 | 5A | 111 | 6.18E-04 | 5.96 |
| Mn | E1 | wsnp_Ex_c54655_57455110 | 5A | 111 | 6.18E-04 | 5.96 |
| Mn | E1 | wsnp_Ku_c3953_7233359 | 5A | 111 | 6.18E-04 | 5.96 |
| Mn | E1 | BobWhite_c2830_327 | 5A | 111 | 6.18E-04 | 5.96 |
| Mn | E1 | IAAV3527 | 5A | 111 | 6.18E-04 | 5.96 |
| Mn | E1 | RAC875_c55872_149 | 5A | 111 | 6.18E-04 | 5.96 |
| Mn | E1 | TA003720-0955 | 5A | 111 | 6.18E-04 | 5.96 |
| Mn | E1 | Tdurum_contig17697_675 | 7A | 136 | 6.04E-04 | 5.98 |
| Mn | E1 | Excalibur_c5700_670 | 7B | 85 | 8.57E-04 | 5.64 |
| Mn | E1 | wsnp_Ex_c7934_13467460 | 7B | 134 | 4.39E-04 | 6.29 |
| Mn | E1 | Excalibur_c1055_565 | 7B | 134 | 7.02E-04 | 5.83 |
| Mn | E1 | IACX11047 | 7B | 134 | 7.02E-04 | 5.83 |
| Mn | E1 | IACX11443 | 7B | 134 | 7.02E-04 | 5.83 |
| Mn | E1 | Ku_c203_584 | 7B | 134 | 1.65E-04 | 7.26 |
| Mn | E1 | RAC875_c4693_554 | 7B | 134 | 7.02E-04 | 5.83 |
| Mn | E1 | BS00012264_51 | 7B | 134 | 7.02E-04 | 5.83 |
| Mn | E1 | Ra_c26852_560 | 7B | 135 | 2.58E-04 | 6.88 |
| Mn | E1 | Ra_c26852_957 | 7B | 135 | 6.38E-04 | 5.94 |
| Mn | E1 | RAC875_rep_c78007_425 | 7B | 135 | 9.73E-04 | 5.52 |
| Mn | E1 | IAAV6137 | 7B | 136 | 8.45E-04 | 5.66 |
| Mn | E1 | wsnp_Ra_c20167_29417876 | 7B | 136 | 1.65E-04 | 7.26 |
| Mn | E1 | BS00108264_51 | 7B | 136 | 2.84E-04 | 6.80 |
| Mn | E1 | IAAV3313 | 7B | 136 | 1.65E-04 | 7.26 |
| Mn | E1 | Kukri_rep_c72901_271 | 7B | 136 | 1.65E-04 | 7.26 |
| Mn | E1 | Tdurum_contig43954_1287 | 7B | 136 | 1.65E-04 | 7.26 |
| Mn | E1 | Tdurum_contig43954_2291 | 7B | 136 | 6.38E-04 | 5.94 |
| Mn | E2 | BobWhite_rep_c66032_270 | 1B | 71 | 2.38E-04 | 6.99 |
| Mn | E2 | wsnp_BE443332B_Ta_2_2 | 1B | 71 | 2.21E-04 | 7.05 |
| Mn | E2 | wsnp_BE443930B_Ta_2_2 | 1B | 71 | 2.82E-04 | 6.81 |
| Mn | E2 | wsnp_Ex_c4561_8184576 | 1B | 75 | 6.26E-05 | 8.33 |
| Mn | E2 | wsnp_BF478690B_Ta_2_1 | 1B | 75 | 6.72E-05 | 8.28 |
| Mn | E2 | Excalibur_c5218_75 | 1B | 75 | 7.28E-05 | 8.20 |
| Mn | E2 | IAAV2125 | 1B | 75 | 6.72E-05 | 8.28 |
| Mn | E2 | IAAV6731 | 1B | 75 | 6.72E-05 | 8.28 |
| Mn | E2 | IAAV9005 | 1B | 75 | 1.12E-04 | 7.74 |
| Mn | E2 | JD_c3116_778 | 1B | 75 | 6.26E-05 | 8.33 |
| Mn | E2 | Ra_c37969_549 | 1B | 75 | 8.25E-05 | 8.05 |
| Mn | E2 | RAC875_c19014_725 | 1B | 75 | 6.26E-05 | 8.33 |
| Mn | E2 | RAC875_rep_c112555_200 | 1B | 75 | 6.72E-05 | 8.28 |
| Mn | E2 | RAC875_rep_c119728_146 | 1B | 75 | 8.92E-05 | 7.97 |
| Mn | E2 | TA003725-0553 | 1B | 75 | 8.92E-05 | 7.97 |
| Mn | E2 | BS00022619_51 | 1B | 75 | 7.72E-05 | 8.14 |
| Mn | E2 | BS00022920_51 | 1B | 75 | 6.72E-05 | 8.28 |
| Mn | E2 | Tdurum_contig81102_102 | 1B | 75 | 8.92E-05 | 7.97 |
| Mn | E2 | Tdurum_contig7144_602 | 2B | 108 | 8.10E-04 | 7.09 |
| Mn | E2 | BS00084130_51 | 2B | 112 | 3.25E-04 | 6.72 |
| Mn | E2 | Ku_c26872_269 | 3A | 174 | 5.91E-04 | 6.14 |
| Mn | E2 | D_contig76625_350 | 4A | 123 | 9.56E-04 | 5.60 |
| Mn | E2 | Kukri_c11415_1074 | 4B | 68 | 9.53E-04 | 5.62 |
| Mn | E2 | Ku_c10415_662 | 5B | 72 | 3.34E-04 | 6.85 |
| Mn | E4 | wsnp_Ex_c4436_7981037 | 1B | 142 | 5.76E-04 | 6.35 |
| Mn | E4 | IAAV80 | 2A | 104 | 8.66E-04 | 5.92 |
| Mn | E4 | Tdurum_contig30451_88 | 2A | 104 | 8.66E-04 | 5.92 |
| Mn | E4 | wsnp_Ex_c9909_16316813 | 7B | 53 | 9.06E-04 | 5.87 |
| Mn | E4 | Excalibur_c62837_164 | 7B | 77 | 8.45E-04 | 5.95 |
| Mn | E4 | Kukri_c2796_1436 | 7B | 77 | 8.45E-04 | 5.95 |
| Mn | E4 | RAC875_c60161_1223 | 7B | 77 | 9.10E-04 | 5.93 |
| Fe | E1 | Tdurum_contig32437_257 | 1A | 82 | 7.25E-04 | 5.83 |
| Fe | E1 | RAC875_rep_c120644_74 | 1A | 94 | 4.76E-04 | 6.17 |
| Fe | E1 | wsnp_Ex_c23968_33209733 | 5A | 111 | 8.43E-04 | 5.62 |
| Fe | E1 | wsnp_Ex_c23968_33210208 | 5A | 111 | 8.43E-04 | 5.62 |
| Fe | E1 | wsnp_Ex_c23968_33210344 | 5A | 111 | 8.43E-04 | 5.62 |
| Fe | E1 | wsnp_Ex_c54655_57455110 | 5A | 111 | 8.43E-04 | 5.62 |
| Fe | E1 | wsnp_Ku_c3953_7233359 | 5A | 111 | 8.43E-04 | 5.62 |
| Fe | E1 | BobWhite_c2830_327 | 5A | 111 | 8.43E-04 | 5.62 |
| Fe | E1 | IAAV3527 | 5A | 111 | 8.43E-04 | 5.62 |
| Fe | E1 | RAC875_c55872_149 | 5A | 111 | 8.43E-04 | 5.62 |
| Fe | E1 | TA003720-0955 | 5A | 111 | 8.43E-04 | 5.62 |
| Fe | E1 | Tdurum_contig47317_100 | 7B | 113 | 9.36E-04 | 5.59 |
| Fe | E1 | Kukri_c29396_58 | 7B | 114 | 8.02E-04 | 5.67 |
| Fe | E1 | BS00066647_51 | 7B | 114 | 6.82E-04 | 5.83 |
| Fe | E1 | BS00023166_51 | 7B | 134 | 2.88E-04 | 6.99 |
| Fe | E1 | Excalibur_c3738_1316 | 7B | 143 | 5.68E-04 | 6.01 |
| Fe | E1 | Tdurum_contig81587_90 | 7B | 143 | 5.13E-04 | 6.10 |
| Fe | E1 | Kukri_c28160_2017 | 7B | 147 | 6.14E-04 | 5.93 |
| Fe | E2 | TA001371-0399 | 1D | 105 | 7.89E-04 | 5.97 |
| Fe | E2 | wsnp_RFL_Contig2506_2098552 | 2B | 96 | 7.28E-04 | 6.06 |
| Fe | E2 | Tdurum_contig50389_317 | 3A | 95 | 7.44E-04 | 6.03 |
| Fe | E2 | Tdurum_contig75336_402 | 3A | 191 | 7.37E-04 | 6.05 |
| Fe | E2 | Excalibur_c14217_1260 | 4A | 114 | 4.24E-04 | 6.61 |
| Fe | E2 | Ra_c16330_1197 | 4A | 114 | 4.74E-04 | 7.08 |
| Fe | E2 | TA004912-0408 | 4A | 114 | 4.24E-04 | 6.61 |
| Fe | E2 | wsnp_Ex_c23363_32607637 | 4B | 70 | 7.93E-04 | 7.77 |
| Fe | E2 | wsnp_Ex_c11913_19105189 | 5A | 16 | 5.47E-05 | 8.75 |
| Fe | E2 | RAC875_rep_c112368_118 | 5A | 16 | 7.57E-05 | 8.77 |
| Fe | E2 | BobWhite_c44797_294 | 5B | 44 | 6.29E-04 | 6.21 |
| Fe | E2 | wsnp_RFL_Contig2731_2449789 | 5B | 46 | 6.29E-04 | 6.21 |
| Fe | E2 | Kukri_c19299_270 | 6B | 0 | 4.75E-04 | 6.51 |
| Fe | E2 | Excalibur_c6326_77 | 6B | 20 | 1.46E-05 | 10.19 |
| Fe | E2 | Excalibur_c50559_622 | 6B | 21 | 8.32E-04 | 5.92 |
| Fe | E2 | RAC875_s114363_172 | 6B | 22 | 5.14E-05 | 8.87 |
| Fe | E2 | Excalibur_c50559_81 | 6B | 22 | 2.19E-04 | 7.29 |
| Fe | E2 | Excalibur_c55093_143 | 6B | 22 | 1.84E-04 | 7.47 |
| Fe | E2 | RAC875_c62256_904 | 6B | 22 | 1.84E-04 | 7.47 |
| Fe | E2 | BS00022499_51 | 6B | 24 | 8.24E-04 | 6.76 |
| Fe | E2 | IACX9024 | 6B | 39 | 5.30E-04 | 6.40 |
| Fe | E2 | Kukri_c61725_362 | 6B | 39 | 9.16E-04 | 5.83 |
| Fe | E2 | RFL_Contig5693_807 | 6B | 39 | 5.40E-04 | 6.38 |
| Fe | E2 | Tdurum_contig9612_971 | 6B | 39 | 5.36E-04 | 6.39 |
| Fe | E3 | Kukri_c18677_237 | 4A | 144 | 1.10E-04 | 8.18 |
| Fe | E3 | wsnp_Ku_c16354_25219645 | 4D | 154 | 1.18E-04 | 7.80 |
| Fe | E3 | Tdurum_contig46954_406 | 7A | 44 | 1.84E-04 | 7.34 |
| Fe | E3 | Excalibur_c41298_459 | 7B | 49 | 4.52E-04 | 6.44 |
| Fe | E3 | TA002655-1964 | 7B | 49 | 5.13E-04 | 6.31 |
| Fe | E3 | Kukri_s109646_139 | 7B | 157 | 4.47E-04 | 6.47 |
| Fe | E3 | BS00010819_51 | 7B | 157 | 5.47E-04 | 6.24 |
| Fe | E3 | wsnp_Ex_c16577_25095267 | 7B | 157 | 5.47E-04 | 6.24 |
| Fe | E3 | BS00047623_51 | 7B | 159 | 7.76E-04 | 6.26 |
| Fe | E3 | RAC875_rep_c118995_572 | 7D | 26 | 6.40E-04 | 6.10 |
| Fe | E3 | wsnp_Ex_c65899_64135487 | 7D | 26 | 8.79E-05 | 8.12 |
| Fe | E3 | wsnp_Ra_c8297_14095831 | 7D | 26 | 9.09E-05 | 8.07 |
| Fe | E3 | D_contig11494_202 | 7D | 26 | 7.70E-05 | 8.25 |
| Fe | E3 | D_F5XZDLF01ASSE2_190 | 7D | 26 | 7.70E-05 | 8.25 |
| Fe | E3 | Ex_c25027_535 | 7D | 26 | 7.70E-05 | 8.25 |
| Fe | E3 | Excalibur_c833_1405 | 7D | 26 | 8.85E-05 | 8.10 |
| Fe | E3 | Kukri_rep_c103404_314 | 7D | 26 | 7.70E-05 | 8.25 |
| Fe | E3 | BS00022449_51 | 7D | 26 | 7.82E-05 | 8.25 |
| Fe | E3 | BS00110124_51 | 7D | 27 | 7.70E-05 | 8.25 |
| Fe | E3 | BS00110642_51 | 7D | 27 | 7.70E-05 | 8.25 |
| Fe | E3 | D_GB5Y7FA02IDDA9_183 | 7D | 30 | 7.70E-05 | 8.25 |
| Fe | E3 | TA005377-1076 | 7D | 32 | 6.50E-05 | 8.43 |
| Fe | E4 | Excalibur_rep_c74900_73 | 4A | 89 | 9.94E-04 | 5.79 |
| Fe | E4 | GENE-3134_472 | 4B | 60 | 5.39E-04 | 6.48 |
| Fe | E4 | Tdurum_contig33826_290 | 4B | 60 | 2.58E-04 | 7.20 |
| Fe | E4 | Tdurum_contig61465_781 | 4B | 61 | 8.45E-04 | 5.95 |
| Fe | E4 | IAAV6287 | 7A | 139 | 2.10E-04 | 7.47 |
| Fe | E4 | Excalibur_c95707_285 | 7A | 152 | 7.70E-04 | 6.05 |
| Fe | E4 | BS00021657_51 | 7A | 152 | 7.70E-04 | 6.05 |
| Fe | E4 | Excalibur_c19455_3496 | 7B | 163 | 8.12E-08 | 16.23 |
| Fe | E4 | Excalibur_c11062_582 | 7B | 171 | 1.44E-05 | 10.26 |
| Fe | E4 | Excalibur_c25090_830 | 7B | 171 | 2.81E-06 | 12.07 |
| Fe | E4 | RAC875_c10585_181 | 7B | 171 | 2.24E-04 | 9.32 |
| Fe | E4 | RAC875_c34939_963 | 7B | 171 | 6.96E-04 | 6.15 |
| Fe | E4 | RAC875_rep_c110526_229 | 7B | 171 | 1.44E-05 | 10.26 |
| Cu | E1 | BS00096498_51 | 1B | 107 | 9.48E-04 | 5.60 |
| Cu | E1 | wsnp_Ex_c53983_57032473 | 5A | 84 | 2.13E-04 | 7.07 |
| Cu | E1 | wsnp_Ex_c59520_60358626 | 5A | 84 | 2.13E-04 | 7.09 |
| Cu | E1 | BS00109396_51 | 5A | 84 | 2.68E-04 | 6.84 |
| Cu | E1 | IAAV4104 | 5A | 84 | 2.13E-04 | 7.09 |
| Cu | E1 | Jagger_c8122_139 | 5A | 84 | 2.00E-04 | 7.16 |
| Cu | E1 | Ku_c13370_656 | 5A | 84 | 9.30E-04 | 6.14 |
| Cu | E1 | Kukri_c5967_586 | 5A | 84 | 4.74E-04 | 6.27 |
| Cu | E1 | RAC875_c23775_406 | 5A | 84 | 6.85E-04 | 5.95 |
| Cu | E1 | RAC875_c25339_200 | 5A | 84 | 8.64E-04 | 5.70 |
| Cu | E1 | RFL_Contig379_355 | 5A | 84 | 2.51E-04 | 6.95 |
| Cu | E1 | Tdurum_contig36142_150 | 5A | 84 | 8.64E-04 | 5.70 |
| Cu | E1 | Tdurum_contig82190_124 | 5A | 84 | 6.20E-04 | 6.05 |
| Cu | E1 | wsnp_Ex_rep_c69647_68598463 | 5A | 87 | 5.84E-04 | 6.09 |
| Cu | E1 | wsnp_Ex_rep_c69647_68598487 | 5A | 87 | 7.89E-04 | 5.81 |
| Cu | E1 | Excalibur_c45297_316 | 5A | 87 | 5.84E-04 | 6.09 |
| Cu | E1 | Ra_c50261_717 | 5A | 87 | 5.84E-04 | 6.09 |
| Cu | E1 | wsnp_Ex_c15342_23592789 | 5A | 88 | 5.84E-04 | 6.09 |
| Cu | E1 | BS00029871_51 | 5A | 88 | 8.46E-04 | 5.76 |
| Cu | E1 | Kukri_c96249_58 | 5D | 130 | 2.13E-04 | 7.09 |
| Cu | E1 | Excalibur_c5700_670 | 7B | 85 | 5.32E-04 | 6.16 |
| Cu | E1 | Excalibur_c3738_1316 | 7B | 143 | 8.02E-04 | 5.76 |
| Cu | E1 | Tdurum_contig81587_90 | 7B | 143 | 7.70E-04 | 5.80 |
| Cu | E1 | Kukri_c28160_2017 | 7B | 147 | 2.32E-04 | 6.98 |
| Cu | E1 | RAC875_rep_c84729_461 | 7B | 167 | 9.58E-04 | 5.58 |
| Cu | E1 | D_contig55386_313 | 7D | 146 | 3.65E-04 | 6.53 |
| Cu | E1 | BS00051607_51 | 7D | 146 | 3.65E-04 | 6.53 |
| Cu | E1 | BS00066128_51 | 7D | 146 | 3.65E-04 | 6.53 |
| Cu | E2 | Tdurum_contig56873_1237 | 1B | 71 | 2.67E-04 | 7.08 |
| Cu | E2 | wsnp_BE446240B_Ta_2_1 | 1B | 71 | 1.36E-04 | 7.69 |
| Cu | E2 | CAP7_c3456_113 | 1B | 71 | 2.67E-04 | 7.08 |
| Cu | E2 | BobWhite_c30111_117 | 1B | 71 | 1.33E-04 | 7.73 |
| Cu | E2 | GENE-0142_178 | 1B | 71 | 2.67E-04 | 7.08 |
| Cu | E2 | Kukri_c667_1073 | 1B | 71 | 1.36E-04 | 7.69 |
| Cu | E2 | Kukri_c67707_114 | 1B | 71 | 2.67E-04 | 7.08 |
| Cu | E2 | TA001197-0204 | 1B | 71 | 2.57E-04 | 7.08 |
| Cu | E2 | Tdurum_contig15593_407 | 1B | 71 | 1.36E-04 | 7.69 |
| Cu | E2 | Tdurum_contig28316_243 | 1B | 71 | 1.36E-04 | 7.69 |
| Cu | E2 | Tdurum_contig56874_562 | 1B | 71 | 1.36E-04 | 7.69 |
| Cu | E2 | BS00023142_51 | 1B | 71 | 2.67E-04 | 7.08 |
| Cu | E2 | RAC875_c8878_232 | 1B | 71 | 2.80E-04 | 7.04 |
| Cu | E2 | Tdurum_contig44255_814 | 1B | 72 | 3.96E-04 | 6.60 |
| Cu | E2 | GENE-0165_389 | 1B | 72 | 6.23E-04 | 6.14 |
| Cu | E2 | RFL_Contig3343_2115 | 1B | 72 | 6.23E-04 | 6.14 |
| Cu | E2 | Excalibur_c25566_1000 | 3B | 6 | 5.85E-04 | 6.21 |
| Cu | E2 | Excalibur_c25678_337 | 3B | 72 | 8.47E-04 | 5.85 |
| Cu | E2 | Kukri_c7087_896 | 3B | 72 | 2.20E-04 | 7.20 |
| Cu | E2 | RAC875_rep_c104791_486 | 4A | 140 | 5.64E-04 | 6.37 |
| Cu | E2 | TA004646-0293 | 4A | 140 | 5.17E-04 | 6.36 |
| Cu | E2 | BS00065030_51 | 4A | 142 | 6.46E-04 | 6.42 |
| Cu | E2 | Kukri_c30693_573 | 4A | 151 | 8.16E-04 | 5.88 |
| Cu | E2 | Excalibur_c29255_366 | 4B | 104 | 8.12E-05 | 8.91 |
| Cu | E2 | D_GBB4FNX02G3VH5_90 | 4D | 36 | 7.78E-04 | 5.96 |
| Cu | E2 | BS00029413_51 | 5A | 76 | 8.15E-04 | 6.07 |
| Cu | E2 | wsnp_RFL_Contig4207_4836784 | 5B | 44 | 5.28E-04 | 6.31 |
| Cu | E2 | BS00074894_51 | 6A | 141 | 6.08E-04 | 7.48 |
| Cu | E2 | IAAV1940 | 7A | 89 | 1.20E-04 | 8.05 |
| Cu | E2 | Tdurum_contig14075_328 | 7A | 89 | 2.96E-04 | 6.89 |
| Cu | E2 | Tdurum_contig20214_279 | 7A | 89 | 2.96E-04 | 6.89 |
| Cu | E2 | RAC875_c57326_85 | 7B | 134 | 6.17E-04 | 6.15 |
| Cu | E2 | wsnp_Ex_c8400_14157060 | 7B | 134 | 6.17E-04 | 6.15 |
| Cu | E3 | Excalibur_c96_619 | 2A | 120 | 3.52E-04 | 7.05 |
| Cu | E3 | RAC875_c58006_352 | 2A | 120 | 3.52E-04 | 7.05 |
| Cu | E3 | Excalibur_c21117_300 | 2A | 151 | 8.78E-04 | 6.01 |
| Cu | E3 | Ra_c105904_1191 | 2B | 160 | 8.76E-04 | 5.59 |
| Cu | E3 | IAAV8795 | 2B | 173 | 4.64E-04 | 6.20 |
| Cu | E3 | Ku_c12345_328 | 3B | 80 | 9.59E-04 | 5.50 |
| Cu | E3 | IAAV7104 | 4A | 75 | 6.08E-04 | 5.94 |
| Cu | E3 | RFL_Contig3679_315 | 4A | 75 | 1.50E-04 | 7.41 |
| Cu | E3 | BobWhite_c2506_332 | 5A | 43 | 7.15E-04 | 5.78 |
| Cu | E3 | Kukri_s109646_139 | 7B | 157 | 3.10E-04 | 6.74 |
| Cu | E3 | BS00010819_51 | 7B | 157 | 3.42E-04 | 6.50 |
| Cu | E3 | wsnp_Ex_c16577_25095267 | 7B | 157 | 3.42E-04 | 6.50 |
| Cu | E4 | Kukri_c41943_535 | 1A | 38 | 9.60E-04 | 5.92 |
| Cu | E4 | BobWhite_rep_c49207_243 | 1A | 75 | 7.71E-04 | 6.08 |
| Cu | E4 | BS00063068_51 | 1A | 75 | 7.71E-04 | 6.08 |
| Cu | E4 | Ex_c8514_1342 | 1A | 76 | 8.27E-04 | 6.01 |
| Cu | E4 | IACX3496 | 1A | 76 | 8.27E-04 | 6.01 |
| Cu | E4 | Ra_c6038_588 | 1A | 77 | 7.71E-04 | 6.08 |
| Cu | E4 | RAC875_c23158_301 | 1A | 77 | 6.31E-04 | 6.28 |
| Cu | E4 | RAC875_rep_c106589_784 | 5B | 212 | 6.27E-04 | 6.68 |
| Cu | E4 | Tdurum_contig28552_211 | 5B | 212 | 6.27E-04 | 6.68 |
| Cu | E4 | Tdurum_contig92922_58 | 5B | 212 | 7.45E-04 | 6.53 |
| Cu | E4 | BS00066224_51 | 5D | 172 | 3.58E-04 | 6.87 |
| Zn | E1 | wsnp_JD_c7522_8606553 | 1A | 38 | 3.08E-04 | 7.52 |
| Zn | E1 | BS00022615_51 | 1B | 72 | 9.91E-04 | 5.64 |
| Zn | E1 | IAAV4844 | 1B | 142 | 4.73E-04 | 6.28 |
| Zn | E1 | Tdurum_contig46389_1540 | 1D | 178 | 5.42E-04 | 6.14 |
| Zn | E1 | wsnp_Ex_c52405_56014689 | 2B | 99 | 5.47E-04 | 6.13 |
| Zn | E1 | wsnp_be498599B_Ta_1_1 | 2B | 100 | 5.47E-04 | 6.13 |
| Zn | E1 | wsnp_Ex_c45851_51530126 | 2B | 100 | 5.29E-04 | 6.17 |
| Zn | E1 | wsnp_Ex_c5429_9593668 | 2B | 100 | 5.29E-04 | 6.17 |
| Zn | E1 | wsnp_Ex_c9133_15198714 | 2B | 100 | 7.90E-04 | 5.77 |
| Zn | E1 | wsnp_Ex_rep_c66482_64735785 | 2B | 100 | 5.29E-04 | 6.17 |
| Zn | E1 | wsnp_Ex_rep_c71064_69904031 | 2B | 100 | 5.29E-04 | 6.17 |
| Zn | E1 | BobWhite_c18672_757 | 2B | 100 | 5.29E-04 | 6.17 |
| Zn | E1 | Excalibur_rep_c69016_57 | 2B | 100 | 5.47E-04 | 6.13 |
| Zn | E1 | Kukri_rep_c72413_423 | 2B | 100 | 5.47E-04 | 6.13 |
| Zn | E1 | wsnp_Ex_rep_c103248_88252209 | 2B | 100 | 5.29E-04 | 6.17 |
| Zn | E1 | wsnp_Ku_rep_c71678_71421327 | 2B | 100 | 5.29E-04 | 6.17 |
| Zn | E1 | Excalibur_rep_c111672_114 | 2B | 100 | 5.29E-04 | 6.17 |
| Zn | E1 | wsnp_Ex_c10279_16851747 | 2B | 100 | 5.29E-04 | 6.17 |
| Zn | E1 | RAC875_c20366_376 | 2B | 100 | 5.47E-04 | 6.13 |
| Zn | E1 | BobWhite_rep_c49468_101 | 2B | 100 | 5.46E-04 | 6.15 |
| Zn | E1 | wsnp_Ex_c17538_26261053 | 2B | 100 | 8.36E-04 | 5.77 |
| Zn | E1 | BS00012036_51 | 2B | 108 | 8.49E-05 | 8.00 |
| Zn | E1 | BS00081231_51 | 2B | 109 | 5.39E-04 | 6.15 |
| Zn | E1 | Excalibur_c10634_156 | 2B | 112 | 9.67E-04 | 5.86 |
| Zn | E1 | Kukri_c29052_75 | 2B | 129 | 7.59E-04 | 5.82 |
| Zn | E1 | RAC875_c29367_314 | 3B | 6 | 3.86E-04 | 6.48 |
| Zn | E1 | Tdurum_contig42513_886 | 3B | 12 | 3.86E-04 | 6.48 |
| Zn | E1 | Kukri_c1760_62 | 3B | 14 | 3.86E-04 | 6.48 |
| Zn | E1 | Excalibur_c20559_887 | 3D | 0 | 3.86E-04 | 6.48 |
| Zn | E1 | Excalibur_c24634_68 | 3D | 0 | 2.10E-04 | 7.11 |
| Zn | E1 | Kukri_c46740_226 | 3D | 0 | 1.17E-04 | 7.87 |
| Zn | E1 | RAC875_c29367_349 | 3D | 0 | 3.86E-04 | 6.48 |
| Zn | E1 | Excalibur_c9485_686 | 3D | 4 | 3.81E-04 | 6.49 |
| Zn | E1 | Kukri_rep_c71523_81 | 3D | 67 | 1.71E-04 | 7.29 |
| Zn | E1 | Kukri_c43208_335 | 3D | 67 | 8.80E-04 | 5.68 |
| Zn | E1 | BobWhite_c32217_147 | 4A | 48 | 9.77E-04 | 5.78 |
| Zn | E1 | BS00062691_51 | 4B | 62 | 7.05E-06 | 12.12 |
| Zn | E1 | Kukri_c26092_287 | 4B | 99 | 6.29E-04 | 6.00 |
| Zn | E1 | wsnp_CAP11_c951_572693 | 5A | 39 | 5.11E-04 | 6.21 |
| Zn | E1 | BobWhite_c5917_529 | 5A | 42 | 8.27E-04 | 5.73 |
| Zn | E1 | wsnp_Ex_rep_c107017_90850230 | 5A | 111 | 8.20E-04 | 5.74 |
| Zn | E1 | Excalibur_c11605_156 | 5B | 20 | 3.54E-04 | 6.57 |
| Zn | E1 | Excalibur_c30273_138 | 5B | 20 | 6.62E-04 | 5.95 |
| Zn | E1 | GENE-3207_134 | 5B | 20 | 6.62E-04 | 5.95 |
| Zn | E1 | GENE-3207_610 | 5B | 20 | 6.62E-04 | 5.95 |
| Zn | E1 | Kukri_c23694_370 | 5B | 20 | 3.54E-04 | 6.57 |
| Zn | E1 | BS00065543_51 | 5B | 20 | 3.54E-04 | 6.57 |
| Zn | E1 | BobWhite_c35035_317 | 6A | 136 | 8.48E-04 | 5.70 |
| Zn | E1 | CAP7_c524_297 | 6B | 119 | 3.27E-04 | 6.70 |
| Zn | E1 | Tdurum_contig60988_365 | 6B | 119 | 6.54E-04 | 5.96 |
| Zn | E1 | Kukri_c14511_1046 | 6B | 120 | 9.14E-04 | 5.63 |
| Zn | E1 | RAC875_c2254_1169 | 6B | 121 | 9.14E-04 | 5.63 |
| Zn | E1 | RAC875_c1823_1681 | 6B | 122 | 9.18E-04 | 5.62 |
| Zn | E1 | RAC875_c22501_271 | 6B | 122 | 7.71E-04 | 5.84 |
| Zn | E1 | Kukri_c25930_1245 | 6B | 122 | 9.14E-04 | 5.63 |
| Zn | E1 | CAP8_rep_c6942_227 | 7A | 148 | 8.98E-05 | 7.94 |
| Zn | E1 | BS00072941_51 | 7B | 71 | 2.42E-05 | 9.29 |
| Zn | E1 | BobWhite_c7907_657 | 7B | 71 | 2.42E-05 | 9.29 |
| Zn | E1 | Kukri_c78330_327 | 7B | 71 | 3.23E-05 | 8.99 |
| Zn | E1 | RFL_Contig2540_306 | 7B | 71 | 2.42E-05 | 9.29 |
| Zn | E1 | TA003961-0636 | 7B | 71 | 2.42E-05 | 9.29 |
| Zn | E1 | BS00095819_51 | 7B | 72 | 6.62E-05 | 8.25 |
| Zn | E1 | Tdurum_contig75931_1967 | 7B | 72 | 6.62E-05 | 8.25 |
| Zn | E1 | Excalibur_c5700_670 | 7B | 85 | 9.42E-04 | 5.60 |
| Zn | E1 | BobWhite_c40042_842 | 7B | 101 | 8.98E-05 | 7.94 |
| Zn | E1 | wsnp_Ex_c2123_3988735 | 7D | 128 | 9.46E-04 | 5.60 |
| Zn | E1 | D_contig55386_313 | 7D | 146 | 5.14E-04 | 6.19 |
| Zn | E1 | BS00051607_51 | 7D | 146 | 5.14E-04 | 6.19 |
| Zn | E1 | BS00066128_51 | 7D | 146 | 5.14E-04 | 6.19 |
| Zn | E2 | Excalibur_c97157_65 | 1A | 106 | 3.18E-04 | 7.11 |
| Zn | E2 | BS00028476_51 | 1B | 118 | 7.55E-04 | 5.99 |
| Zn | E2 | Excalibur_c36457_115 | 4B | 102 | 9.62E-04 | 5.73 |
| Zn | E3 | RFL_Contig1493_657 | 1A | 113 | 6.43E-04 | 5.61 |
| Zn | E3 | BS00021851_51 | 1D | 68 | 5.17E-04 | 8.82 |
| Zn | E3 | GENE-1375_20 | 2B | 108 | 5.68E-04 | 5.72 |
| Zn | E3 | Ra_c19225_591 | 2B | 130 | 4.19E-05 | 8.19 |
| Zn | E3 | BS00082967_51 | 2B | 131 | 3.90E-04 | 6.08 |
| Zn | E3 | Ku_c2885_1277 | 4D | 170 | 4.90E-04 | 5.90 |
| Zn | E3 | Ku_c2885_1286 | 4D | 170 | 3.78E-04 | 6.10 |
| Zn | E3 | BS00078603_51 | 5D | 205 | 3.38E-04 | 6.21 |
| Zn | E3 | BS00094333_51 | 5D | 205 | 3.38E-04 | 6.21 |
| Zn | E3 | RAC875_c17224_601 | 6B | 119 | 4.80E-04 | 6.57 |
| Zn | E3 | tplb0045p11_893 | 7A | 148 | 8.30E-04 | 5.37 |
| Zn | E3 | wsnp_Ex_c9428_15641609 | 7A | 159 | 3.63E-05 | 8.42 |
| Zn | E3 | wsnp_Ex_c9428_15641639 | 7A | 159 | 1.91E-05 | 8.98 |
| Zn | E4 | RFL_Contig3713_280 | 2B | 138 | 2.95E-04 | 7.15 |
| Zn | E4 | Tdurum_contig29045_84 | 3A | 109 | 5.59E-04 | 6.26 |
| Zn | E4 | Tdurum_contig21329_326 | 3B | 60 | 4.79E-04 | 6.42 |
| Zn | E4 | Excalibur_c41752_392 | 3B | 67 | 7.87E-07 | 13.25 |
| Zn | E4 | BS00057451_51 | 3B | 67 | 7.87E-07 | 13.25 |
| Zn | E4 | BS00095028_51 | 4B | 6 | 4.85E-04 | 6.48 |
| Zn | E4 | Kukri_c18722_425 | 4B | 91 | 4.39E-04 | 6.50 |
| Zn | E4 | Kukri_c41797_393 | 5A | 53 | 3.90E-04 | 6.62 |
| Zn | E4 | RFL_Contig2187_1025 | 5A | 53 | 3.90E-04 | 6.62 |
| Zn | E4 | Kukri_rep_c103094_177 | 7B | 102 | 4.26E-04 | 6.77 |
| Se | E1 | BS00087600_51 | 1A | 78 | 3.80E-04 | 6.39 |
| Se | E1 | BS00021889_51 | 1A | 78 | 2.27E-04 | 6.96 |
| Se | E1 | Excalibur_rep_c93332_58 | 3D | 107 | 1.07E-05 | 10.06 |
| Se | E1 | BobWhite_c9622_723 | 3D | 113 | 3.93E-05 | 8.99 |
| Se | E1 | Excalibur_c29496_799 | 4A | 49 | 1.34E-04 | 7.42 |
| Se | E1 | Kukri_rep_c74376_188 | 4B | 42 | 6.49E-04 | 5.87 |
| Se | E1 | wsnp_Ku_c21275_31007309 | 5A | 83 | 3.46E-05 | 8.77 |
| Se | E1 | wsnp_Ex_c16295_24772663 | 5A | 111 | 5.72E-04 | 6.07 |
| Se | E1 | wsnp_Ex_c16295_24772702 | 5A | 111 | 3.66E-04 | 6.51 |
| Se | E1 | wsnp_Ex_c23968_33209660 | 5A | 111 | 5.72E-04 | 6.07 |
| Se | E1 | wsnp_Ex_c23968_33209733 | 5A | 111 | 1.93E-04 | 7.05 |
| Se | E1 | wsnp_Ex_c23968_33210208 | 5A | 111 | 1.93E-04 | 7.05 |
| Se | E1 | wsnp_Ex_c23968_33210344 | 5A | 111 | 1.93E-04 | 7.05 |
| Se | E1 | wsnp_Ex_c54655_57455110 | 5A | 111 | 1.93E-04 | 7.05 |
| Se | E1 | wsnp_Ku_c3953_7233359 | 5A | 111 | 1.93E-04 | 7.05 |
| Se | E1 | wsnp_CAP11_c923_558715 | 5A | 111 | 5.72E-04 | 6.07 |
| Se | E1 | BS00085711_51 | 5A | 111 | 5.72E-04 | 6.07 |
| Se | E1 | CAP8_rep_c4852_130 | 5A | 111 | 5.52E-04 | 6.11 |
| Se | E1 | BobWhite_c2830_327 | 5A | 111 | 1.93E-04 | 7.05 |
| Se | E1 | IAAV3527 | 5A | 111 | 1.93E-04 | 7.05 |
| Se | E1 | RAC875_c55872_149 | 5A | 111 | 1.93E-04 | 7.05 |
| Se | E1 | TA003720-0955 | 5A | 111 | 1.93E-04 | 7.05 |
| Se | E1 | RAC875_c59520_130 | 5A | 111 | 3.41E-04 | 6.49 |
| Se | E1 | wsnp_Ra_c11532_18688426 | 5A | 113 | 4.91E-04 | 6.19 |
| Se | E1 | wsnp_BE404947B_Ta_2_12 | 6B | 35 | 7.29E-04 | 5.80 |
| Se | E1 | IAAV1711 | 6B | 35 | 7.29E-04 | 5.80 |
| Se | E1 | Excalibur_c38547_118 | 7B | 77 | 3.31E-04 | 6.58 |
| Se | E1 | Excalibur_c62837_164 | 7B | 77 | 2.60E-04 | 6.76 |
| Se | E1 | Kukri_c2796_1436 | 7B | 77 | 2.60E-04 | 6.76 |
| Se | E1 | RAC875_c60161_448 | 7B | 77 | 5.05E-04 | 6.11 |
| Se | E1 | wsnp_Ex_c6924_11936998 | 7B | 77 | 6.46E-04 | 5.90 |
| Se | E1 | wsnp_Ra_c60161_61164295 | 7B | 77 | 5.05E-04 | 6.11 |
| Se | E1 | wsnp_Ra_c60161_61164325 | 7B | 77 | 8.64E-04 | 5.64 |
| Se | E1 | Jagger_c1882_85 | 7B | 77 | 8.64E-04 | 5.64 |
| Se | E1 | Kukri_rep_c98884_506 | 7B | 77 | 5.05E-04 | 6.11 |
| Se | E1 | RAC875_c60161_281 | 7B | 77 | 6.78E-04 | 5.85 |
| Se | E1 | RAC875_c60770_82 | 7B | 77 | 8.64E-04 | 5.64 |
| Se | E1 | GENE-4900_136 | 7B | 159 | 3.38E-04 | 6.50 |
| Se | E2 | BS00083626_51 | 2B | 173 | 6.58E-04 | 6.09 |
| Se | E2 | BS00054751_51 | 2B | 182 | 9.52E-04 | 6.72 |
| Se | E2 | IAAV5729 | 3A | 61 | 6.01E-04 | 6.18 |
| Se | E2 | RAC875_c47976_291 | 3A | 61 | 6.01E-04 | 6.18 |
| Se | E2 | BS00072153_51 | 3A | 88 | 6.71E-04 | 6.12 |
| Se | E2 | tplb0050j22_569 | 4A | 51 | 8.02E-04 | 5.90 |
| Se | E2 | Tdurum_contig4974_355 | 4B | 61 | 8.65E-05 | 8.70 |
| Se | E2 | Ku_c10647_585 | 6B | 72 | 9.95E-04 | 5.67 |
| Se | E2 | wsnp_Ex_c9750_16105678 | 6B | 72 | 9.95E-04 | 5.67 |
| Se | E2 | RFL_Contig4251_851 | 6B | 72 | 9.95E-04 | 5.67 |
| Se | E2 | wsnp_BE490200B_Ta_2_1 | 6B | 72 | 9.95E-04 | 5.67 |
| Se | E2 | GENE-4204_311 | 6B | 72 | 9.95E-04 | 5.67 |
| Se | E2 | Tdurum_contig66984_143 | 6B | 72 | 9.95E-04 | 5.67 |
| Se | E2 | wsnp_Ex_c14654_22713386 | 7A | 42 | 2.66E-05 | 9.68 |
| Se | E2 | D_contig06359_118 | 7D | 56 | 2.80E-05 | 9.52 |
| Se | E3 | BS00080546_51 | 3A | 123 | 5.80E-04 | 6.22 |
| Se | E3 | BS00022424_51 | 3A | 141 | 1.88E-04 | 7.46 |
| Se | E4 | BS00110078_51 | 4B | 6 | 4.38E-04 | 6.74 |
| Se | E4 | wsnp_JD_c13673_13606066 | 7B | 136 | 3.53E-04 | 6.83 |

E1: 2014DZ; E2: 2015TA; E3: 2016TA; E4: 2017TA.

Table S4 All SNP loci significantly associated with heavy metal elements (P<10^-4^)

| Trait | Env. | SNP Marker | Chr. | Site | P | R^2^(%) |
| --- | --- | --- | --- | --- | --- | --- |
| As | E1 | RAC875_c34888_65 | 1A | 35 | 9.05E-04 | 5.55 |
| As | E1 | wsnp_JD_c7522_8606553 | 1A | 38 | 2.07E-04 | 9.46 |
| As | E1 | RAC875_c14926_589 | 1A | 53 | 6.61E-04 | 5.90 |
| As | E1 | wsnp_BM140362A_Ta_2_2 | 1A | 84 | 1.08E-04 | 7.70 |
| As | E1 | RAC875_c60514_90 | 1A | 85 | 5.76E-04 | 6.01 |
| As | E1 | wsnp_BG606986A_Ta_2_4 | 1A | 103 | 3.82E-04 | 6.39 |
| As | E1 | BS00088350_51 | 1A | 103 | 3.82E-04 | 6.39 |
| As | E1 | IAAV2929 | 3B | 6 | 4.51E-04 | 6.25 |
| As | E1 | BobWhite_rep_c50457_813 | 3B | 67 | 6.41E-04 | 5.88 |
| As | E1 | BS00021849_51 | 3D | 0 | 8.95E-04 | 5.57 |
| As | E1 | CAP8_rep_c4852_130 | 5A | 111 | 4.75E-04 | 6.39 |
| As | E1 | RAC875_c59520_130 | 5A | 111 | 1.26E-04 | 7.48 |
| As | E1 | tplb0032i10_420 | 6A | 77 | 9.79E-04 | 5.48 |
| As | E1 | wsnp_Ku_rep_c112734_95776957 | 6A | 80 | 3.93E-04 | 6.76 |
| As | E1 | BS00100351_51 | 7A | 130 | 4.02E-04 | 6.34 |
| As | E1 | IACX11096 | 7A | 130 | 4.02E-04 | 6.34 |
| As | E1 | Jagger_c319_99 | 7A | 130 | 4.02E-04 | 6.34 |
| As | E1 | BS00040590_51 | 7A | 130 | 4.02E-04 | 6.34 |
| As | E1 | wsnp_JD_c15333_14824351 | 7A | 136 | 4.02E-04 | 6.34 |
| As | E1 | IACX3013 | 7A | 136 | 4.02E-04 | 6.34 |
| As | E1 | BobWhite_c7082_577 | 7B | 78 | 1.85E-04 | 7.38 |
| As | E1 | Excalibur_c5700_670 | 7B | 85 | 8.87E-04 | 5.57 |
| As | E1 | Kukri_c21628_1215 | 7B | 85 | 4.52E-04 | 6.45 |
| As | E1 | Tdurum_contig9966_724 | 7B | 85 | 5.89E-04 | 6.20 |
| As | E1 | RAC875_c4453_2678 | 7D | 112 | 6.53E-04 | 5.87 |
| As | E2 | Excalibur_c37649_125 | 2A | 98 | 1.29E-04 | 7.85 |
| As | E2 | BobWhite_c22277_596 | 2A | 99 | 8.55E-04 | 5.89 |
| As | E2 | IACX1216 | 2A | 99 | 8.56E-04 | 6.00 |
| As | E2 | Excalibur_c4102_1621 | 2B | 82 | 3.83E-04 | 7.27 |
| As | E2 | Ra_c26319_331 | 6B | 72 | 4.42E-04 | 6.63 |
| As | E2 | RAC875_c19357_122 | 6B | 72 | 8.14E-04 | 6.06 |
| As | E2 | wsnp_Ex_c9813_16193536 | 7B | 56 | 7.19E-04 | 6.20 |
| As | E3 | BS00011478_51 | 2A | 139 | 5.95E-04 | 6.29 |
| As | E3 | RAC875_c15396_90 | 2B | 102 | 9.90E-04 | 5.73 |
| As | E3 | RAC875_c59545_122 | 2B | 104 | 1.93E-04 | 7.35 |
| As | E3 | Excalibur_rep_c66577_159 | 2B | 107 | 1.83E-04 | 7.41 |
| As | E3 | BS00100563_51 | 2B | 107 | 6.46E-04 | 6.12 |
| As | E3 | IAAV5674 | 2B | 108 | 5.52E-04 | 6.28 |
| As | E3 | Kukri_c1460_1032 | 2B | 108 | 5.52E-04 | 6.28 |
| As | E3 | GENE-1787_339 | 3B | 70 | 5.81E-04 | 6.23 |
| As | E3 | BS00012080_51 | 3B | 70 | 9.98E-04 | 5.79 |
| As | E3 | RFL_Contig2512_774 | 4A | 144 | 2.58E-04 | 7.20 |
| As | E3 | Excalibur_c58747_289 | 5B | 83 | 5.00E-04 | 6.55 |
| As | E3 | wsnp_CAP12_c475_258416 | 6B | 79 | 2.22E-04 | 7.21 |
| As | E3 | CAP11_rep_c4052_315 | 6B | 79 | 2.07E-04 | 7.43 |
| As | E3 | CAP12_c475_166 | 6B | 79 | 2.22E-04 | 7.21 |
| As | E3 | CAP12_c475_289 | 6B | 79 | 2.22E-04 | 7.21 |
| As | E3 | GENE-4221_519 | 6B | 79 | 2.22E-04 | 7.21 |
| As | E3 | GENE-4221_94 | 6B | 79 | 2.22E-04 | 7.21 |
| As | E3 | IACX1609 | 6B | 79 | 2.22E-04 | 7.21 |
| As | E3 | IACX4538 | 6B | 79 | 2.22E-04 | 7.21 |
| As | E3 | RFL_Contig2387_1410 | 6B | 79 | 2.22E-04 | 7.21 |
| As | E3 | RAC875_c48202_231 | 7A | 42 | 8.31E-04 | 5.87 |
| As | E3 | Kukri_rep_c101179_404 | 7A | 42 | 2.47E-04 | 7.10 |
| As | E3 | BobWhite_rep_c58252_112 | 7A | 84 | 4.88E-04 | 6.40 |
| As | E3 | wsnp_Ex_rep_c105131_89643770 | 7B | 122 | 6.23E-04 | 6.20 |
| As | E3 | BS00068305_51 | 7B | 122 | 7.43E-04 | 6.62 |
| As | E3 | BS00081841_51 | 7B | 122 | 6.23E-04 | 6.20 |
| As | E3 | RAC875_c25019_1132 | 7B | 122 | 6.50E-04 | 6.76 |
| As | E3 | GENE-3129_890 | 7D | 91 | 7.49E-04 | 6.65 |
| As | E4 | wsnp_Ex_c4774_8519623 | 1B | 132 | 5.07E-04 | 6.48 |
| As | E4 | GENE-0063_68 | 1B | 133 | 1.66E-04 | 7.62 |
| As | E4 | Tdurum_contig57153_1356 | 1B | 133 | 1.66E-04 | 7.62 |
| As | E4 | Tdurum_contig10475_87 | 1B | 134 | 7.15E-04 | 6.11 |
| As | E4 | Tdurum_contig25612_195 | 1B | 134 | 7.15E-04 | 6.11 |
| As | E4 | Tdurum_contig54785_216 | 5A | 82 | 9.90E-04 | 5.78 |
| As | E4 | Tdurum_contig54785_62 | 5A | 82 | 9.85E-04 | 5.79 |
| Cd | E1 | Excalibur_c30368_56 | 3D | 0 | 9.19E-04 | 6.17 |
| Cd | E2 | Kukri_rep_c72587_123 | 1D | 68 | 1.69E-04 | 7.66 |
| Cd | E2 | RAC875_c17951_108 | 1D | 68 | 2.42E-04 | 7.18 |
| Cd | E2 | BS00051826_51 | 1D | 68 | 4.49E-04 | 6.52 |
| Cd | E2 | Tdurum_contig36962_250 | 1D | 71 | 1.17E-04 | 7.92 |
| Cd | E2 | wsnp_CAP8_c458_368125 | 1D | 72 | 1.17E-04 | 7.92 |
| Cd | E2 | wsnp_CAP8_c458_368155 | 1D | 72 | 1.17E-04 | 7.92 |
| Cd | E2 | BS00083531_51 | 1D | 72 | 6.82E-05 | 8.51 |
| Cd | E2 | Excalibur_c34167_128 | 1D | 72 | 1.17E-04 | 7.92 |
| Cd | E2 | Excalibur_c4030_1770 | 1D | 72 | 1.17E-04 | 7.92 |
| Cd | E2 | Kukri_c49577_630 | 1D | 72 | 1.28E-04 | 8.08 |
| Cd | E2 | Kukri_c6167_1271 | 1D | 72 | 1.17E-04 | 7.92 |
| Cd | E2 | RAC875_c48997_1160 | 1D | 72 | 1.17E-04 | 7.92 |
| Cd | E2 | BS00024262_51 | 1D | 72 | 1.17E-04 | 7.92 |
| Cd | E2 | BS00033405_51 | 1D | 72 | 1.17E-04 | 7.92 |
| Cd | E2 | CAP8_c879_120 | 1D | 73 | 4.49E-04 | 6.52 |
| Cd | E2 | Excalibur_c5958_1398 | 1D | 73 | 2.42E-04 | 7.18 |
| Cd | E2 | IACX6432 | 1D | 73 | 2.78E-04 | 7.08 |
| Cd | E2 | BS00031658_51 | 1D | 73 | 4.49E-04 | 6.52 |
| Cd | E2 | CAP12_rep_c6956_169 | 2A | 115 | 7.91E-05 | 8.32 |
| Cd | E2 | IAAV1634 | 2B | 70 | 8.77E-04 | 5.84 |
| Cd | E2 | wsnp_RFL_Contig2506_2098552 | 2B | 96 | 2.19E-04 | 7.26 |
| Cd | E2 | IACX3325 | 2B | 96 | 7.68E-04 | 5.98 |
| Cd | E2 | BS00001140_51 | 2B | 96 | 7.68E-04 | 5.98 |
| Cd | E2 | BS00092542_51 | 2B | 97 | 7.68E-04 | 5.98 |
| Cd | E2 | wsnp_Ex_c6248_10896799 | 2B | 99 | 2.39E-04 | 7.18 |
| Cd | E2 | wsnp_Ku_c11850_19271281 | 2B | 99 | 2.28E-04 | 7.22 |
| Cd | E2 | wsnp_Ex_c52405_56014689 | 2B | 99 | 6.09E-04 | 6.23 |
| Cd | E2 | wsnp_Ra_c4746_8540731 | 2B | 99 | 2.28E-04 | 7.22 |
| Cd | E2 | wsnp_Ex_c1568_2993540 | 2B | 100 | 2.28E-04 | 7.22 |
| Cd | E2 | wsnp_be498599B_Ta_1_1 | 2B | 100 | 6.09E-04 | 6.23 |
| Cd | E2 | wsnp_Ex_c39862_47046812 | 2B | 100 | 4.51E-04 | 6.52 |
| Cd | E2 | wsnp_Ex_c45851_51530126 | 2B | 100 | 5.82E-04 | 6.26 |
| Cd | E2 | wsnp_Ex_c5429_9593668 | 2B | 100 | 5.82E-04 | 6.26 |
| Cd | E2 | wsnp_Ex_c59991_60628628 | 2B | 100 | 2.28E-04 | 7.22 |
| Cd | E2 | wsnp_Ex_c7516_12850225 | 2B | 100 | 2.39E-04 | 7.18 |
| Cd | E2 | wsnp_Ex_c9133_15198714 | 2B | 100 | 5.82E-04 | 6.26 |
| Cd | E2 | wsnp_Ex_rep_c66482_64735785 | 2B | 100 | 5.82E-04 | 6.26 |
| Cd | E2 | wsnp_Ex_rep_c66482_64736708 | 2B | 100 | 2.28E-04 | 7.22 |
| Cd | E2 | wsnp_Ex_rep_c69016_67915892 | 2B | 100 | 2.39E-04 | 7.18 |
| Cd | E2 | wsnp_Ex_rep_c71064_69904031 | 2B | 100 | 5.82E-04 | 6.26 |
| Cd | E2 | BobWhite_c18672_757 | 2B | 100 | 5.82E-04 | 6.26 |
| Cd | E2 | Ex_c66545_551 | 2B | 100 | 2.39E-04 | 7.18 |
| Cd | E2 | Excalibur_rep_c69016_57 | 2B | 100 | 6.09E-04 | 6.23 |
| Cd | E2 | Kukri_rep_c72413_423 | 2B | 100 | 6.09E-04 | 6.23 |
| Cd | E2 | BobWhite_rep_c54390_73 | 2B | 100 | 2.28E-04 | 7.22 |
| Cd | E2 | RFL_Contig3301_510 | 2B | 100 | 2.28E-04 | 7.22 |
| Cd | E2 | RFL_Contig4748_1076 | 2B | 100 | 2.28E-04 | 7.22 |
| Cd | E2 | wsnp_Ex_c20529_29609310 | 2B | 100 | 2.32E-04 | 7.21 |
| Cd | E2 | wsnp_Ex_c21532_30680512 | 2B | 100 | 2.28E-04 | 7.22 |
| Cd | E2 | wsnp_Ex_c36002_44045355 | 2B | 100 | 2.39E-04 | 7.18 |
| Cd | E2 | wsnp_Ex_rep_c70228_69172301 | 2B | 100 | 2.28E-04 | 7.22 |
| Cd | E2 | wsnp_JD_c9251_10121369 | 2B | 100 | 2.39E-04 | 7.18 |
| Cd | E2 | wsnp_CAP11_rep_c4105_1940985 | 2B | 100 | 2.28E-04 | 7.22 |
| Cd | E2 | Excalibur_c17155_636 | 2B | 100 | 2.39E-04 | 7.18 |
| Cd | E2 | BobWhite_c16735_131 | 2B | 100 | 2.39E-04 | 7.18 |
| Cd | E2 | BobWhite_c16735_176 | 2B | 100 | 2.34E-04 | 7.20 |
| Cd | E2 | Excalibur_c3839_2483 | 2B | 100 | 2.28E-04 | 7.22 |
| Cd | E2 | wsnp_Ex_rep_c103248_88252209 | 2B | 100 | 5.82E-04 | 6.26 |
| Cd | E2 | wsnp_Ex_rep_c103248_88252281 | 2B | 100 | 2.39E-04 | 7.18 |
| Cd | E2 | wsnp_Ku_rep_c71678_71421327 | 2B | 100 | 5.82E-04 | 6.26 |
| Cd | E2 | Excalibur_rep_c111672_114 | 2B | 100 | 5.82E-04 | 6.26 |
| Cd | E2 | wsnp_Ex_c10279_16851747 | 2B | 100 | 5.82E-04 | 6.26 |
| Cd | E2 | wsnp_Ex_c28243_37383894 | 2B | 100 | 2.39E-04 | 7.18 |
| Cd | E2 | wsnp_Ex_c29434_38471452 | 2B | 100 | 2.43E-04 | 7.20 |
| Cd | E2 | wsnp_Ex_c482_957514 | 2B | 100 | 2.28E-04 | 7.22 |
| Cd | E2 | BobWhite_c3757_55 | 2B | 100 | 2.28E-04 | 7.22 |
| Cd | E2 | Kukri_c5609_565 | 2B | 100 | 2.39E-04 | 7.18 |
| Cd | E2 | RAC875_c20366_376 | 2B | 100 | 6.09E-04 | 6.23 |
| Cd | E2 | RAC875_c20950_461 | 2B | 100 | 2.20E-04 | 7.29 |
| Cd | E2 | RAC875_c21237_1064 | 2B | 100 | 2.28E-04 | 7.22 |
| Cd | E2 | RAC875_c60230_122 | 2B | 100 | 2.28E-04 | 7.22 |
| Cd | E2 | RFL_Contig3353_125 | 2B | 100 | 2.28E-04 | 7.22 |
| Cd | E2 | Excalibur_c3869_427 | 2B | 100 | 2.28E-04 | 7.22 |
| Cd | E2 | BobWhite_rep_c49468_101 | 2B | 100 | 5.73E-04 | 6.28 |
| Cd | E2 | RAC875_c21237_166 | 2B | 100 | 2.28E-04 | 7.22 |
| Cd | E2 | wsnp_Ex_c17538_26261053 | 2B | 100 | 8.63E-05 | 8.25 |
| Cd | E2 | RAC875_c59545_122 | 2B | 104 | 7.26E-05 | 8.41 |
| Cd | E2 | Excalibur_rep_c66577_159 | 2B | 107 | 5.00E-05 | 8.82 |
| Cd | E2 | IAAV5674 | 2B | 108 | 7.12E-04 | 6.06 |
| Cd | E2 | Kukri_c1460_1032 | 2B | 108 | 7.12E-04 | 6.06 |
| Cd | E2 | D_GBB4FNX02G3VH5_90 | 4D | 36 | 2.72E-04 | 7.06 |
| Cd | E2 | Kukri_c76365_555 | 6D | 153 | 3.49E-04 | 6.82 |
| Cd | E2 | Kukri_rep_c107605_164 | 6D | 153 | 4.72E-04 | 6.47 |
| Cd | E2 | Excalibur_c20018_214 | 7A | 203 | 5.81E-04 | 6.66 |
| Cd | E2 | BS00063821_51 | 7B | 167 | 7.36E-04 | 6.02 |
| Cd | E2 | RFL_Contig3005_1031 | 7B | 171 | 7.56E-04 | 6.01 |
| Cd | E3 | Tdurum_contig9811_172 | 1B | 53 | 6.22E-04 | 6.34 |
| Cd | E3 | Ex_c4206_502 | 1B | 108 | 9.45E-04 | 5.77 |
| Cd | E3 | Tdurum_contig44851_927 | 1B | 162 | 3.23E-06 | 11.77 |
| Cd | E3 | Kukri_rep_c72412_856 | 2A | 79 | 4.56E-04 | 6.51 |
| Cd | E3 | Ex_c5280_1429 | 2A | 113 | 1.16E-04 | 7.92 |
| Cd | E3 | BS00039422_51 | 2A | 115 | 9.89E-04 | 5.74 |
| Cd | E3 | Ra_c19225_591 | 2B | 130 | 5.51E-04 | 6.32 |
| Cd | E3 | IAAV283 | 2B | 134 | 8.73E-04 | 5.85 |
| Cd | E3 | RAC875_c57829_83 | 3B | 62 | 4.22E-04 | 6.59 |
| Cd | E3 | wsnp_Ex_c6065_10623213 | 3B | 62 | 6.80E-04 | 6.10 |
| Cd | E3 | wsnp_BE498786B_Ta_2_1 | 3B | 63 | 6.80E-04 | 6.10 |
| Cd | E3 | Excalibur_c11001_134 | 3B | 63 | 6.80E-04 | 6.10 |
| Cd | E3 | Excalibur_c48368_217 | 3B | 63 | 6.80E-04 | 6.10 |
| Cd | E3 | BobWhite_c48009_52 | 3B | 63 | 6.80E-04 | 6.10 |
| Cd | E3 | Ra_c965_2579 | 3B | 63 | 4.16E-04 | 6.88 |
| Cd | E3 | Tdurum_contig13489_292 | 4A | 75 | 3.65E-06 | 11.65 |
| Cd | E3 | Kukri_c59197_207 | 4A | 6 | 7.70E-06 | 11.05 |
| Cd | E3 | RAC875_c9572_588 | 4A | 63 | 8.79E-05 | 8.22 |
| Cd | E3 | BS00094770_51 | 4D | 80 | 2.87E-04 | 6.99 |
| Cd | E3 | RAC875_c64253_435 | 5A | 83 | 8.35E-04 | 5.92 |
| Cd | E3 | RAC875_rep_c69613_547 | 5D | 56 | 6.85E-05 | 9.10 |
| Cd | E3 | BS00067630_51 | 6A | 13 | 4.20E-04 | 6.60 |
| Cd | E3 | RFL_Contig5170_1904 | 6A | 16 | 4.20E-04 | 6.60 |
| Cd | E3 | BS00031057_51 | 6A | 17 | 4.20E-04 | 6.60 |
| Cd | E3 | BobWhite_c32981_113 | 6A | 41 | 6.16E-04 | 6.25 |
| Cd | E3 | wsnp_Ex_rep_c66315_64480670 | 6B | 72 | 6.38E-04 | 6.17 |
| Cd | E3 | wsnp_Ex_c7713_13153321 | 6B | 92 | 4.09E-05 | 9.03 |
| Cd | E3 | Excalibur_c7713_272 | 6B | 92 | 8.57E-05 | 8.24 |
| Cd | E3 | wsnp_Ex_c9428_15641609 | 7A | 159 | 6.85E-04 | 6.12 |
| Cd | E4 | Kukri_c105601_74 | 1B | 51 | 2.15E-04 | 7.29 |
| Cd | E4 | RAC875_c25101_644 | 1B | 51 | 2.15E-04 | 7.29 |
| Cd | E4 | wsnp_Ku_c11987_19472688 | 1B | 57 | 2.15E-04 | 7.29 |
| Cd | E4 | Kukri_c18006_1568 | 1B | 57 | 2.15E-04 | 7.29 |
| Cd | E4 | RAC875_c8271_1352 | 1B | 57 | 2.15E-04 | 7.29 |
| Cd | E4 | RAC875_c8271_1469 | 1B | 57 | 2.15E-04 | 7.29 |
| Cd | E4 | RAC875_c8271_887 | 1B | 57 | 2.15E-04 | 7.29 |
| Cd | E4 | RAC875_rep_c96733_369 | 1B | 57 | 2.15E-04 | 7.29 |
| Cd | E4 | BS00022255_51 | 1B | 57 | 1.50E-04 | 7.67 |
| Cd | E4 | wsnp_Ku_c11987_19473636 | 1B | 58 | 2.06E-04 | 7.35 |
| Cd | E4 | D_contig25392_201 | 1B | 61 | 1.50E-04 | 7.67 |
| Cd | E4 | Kukri_c23300_267 | 1B | 61 | 2.15E-04 | 7.29 |
| Cd | E4 | RAC875_c9594_1289 | 1D | 65 | 9.63E-05 | 8.20 |
| Cd | E4 | Kukri_c209_999 | 2A | 143 | 8.13E-04 | 6.12 |
| Cd | E4 | BS00091763_51 | 2A | 167 | 2.43E-04 | 7.17 |
| Cd | E4 | wsnp_Ex_rep_c108004_91402649 | 2A | 168 | 4.77E-05 | 8.87 |
| Cd | E4 | GENE-0762_808 | 2A | 168 | 4.64E-05 | 8.90 |
| Cd | E4 | RAC875_c17479_359 | 3A | 93 | 3.22E-04 | 6.88 |
| Cd | E4 | BS00022424_51 | 3A | 141 | 6.00E-04 | 6.24 |
| Cd | E4 | Tdurum_contig61465_781 | 4B | 61 | 1.40E-04 | 7.74 |
| Cd | E4 | wsnp_Ex_c28908_37989067 | 5A | 27 | 4.85E-06 | 11.34 |
| Cd | E4 | wsnp_Ku_c1254_2498515 | 5A | 27 | 1.43E-05 | 10.16 |
| Cd | E4 | BS00066569_51 | 5A | 48 | 5.79E-04 | 6.28 |
| Cd | E4 | BS00075819_51 | 5B | 117 | 1.60E-04 | 7.63 |
| Cd | E4 | Excalibur_c6458_593 | 5B | 117 | 1.85E-04 | 7.45 |
| Cd | E4 | IAAV5479 | 5B | 117 | 1.85E-04 | 7.45 |
| Cd | E4 | BS00033185_51 | 5B | 174 | 9.69E-05 | 8.14 |
| Cd | E4 | Excalibur_c23452_401 | 5B | 178 | 7.96E-04 | 5.95 |
| Cd | E4 | Kukri_c1214_2316 | 5B | 178 | 7.96E-04 | 5.95 |
| Cd | E4 | Kukri_c1214_825 | 5B | 178 | 7.96E-04 | 5.95 |
| Cd | E4 | RAC875_c60161_243 | 7A | 136 | 3.72E-04 | 6.73 |
| Cd | E4 | CAP11_c3781_95 | 7A | 136 | 4.95E-04 | 6.43 |
| Cd | E4 | BobWhite_c17095_237 | 7A | 136 | 3.98E-04 | 6.66 |
| Cd | E4 | Excalibur_c19455_3496 | 7B | 163 | 2.18E-04 | 7.33 |
| Cd | E4 | Excalibur_c11062_582 | 7B | 171 | 5.52E-05 | 8.72 |
| Cd | E4 | Excalibur_c25090_830 | 7B | 171 | 2.68E-05 | 9.49 |
| Cd | E4 | RAC875_rep_c110526_229 | 7B | 171 | 5.52E-05 | 8.72 |
| Pb | E1 | CAP8_rep_c5126_147 | 1B | 63 | 4.05E-04 | 6.45 |
| Pb | E1 | wsnp_Ex_c30695_39579408 | 4b | 45 | 7.48E-04 | 5.81 |
| Pb | E1 | RAC875_c26487_145 | 6A | 79 | 1.98E-04 | 7.14 |
| Pb | E1 | Kukri_c29591_1356 | 6B | 58 | 2.99E-04 | 6.72 |
| Pb | E1 | Excalibur_c16961_85 | 6B | 64 | 2.15E-05 | 9.44 |
| Pb | E1 | Kukri_c36620_1336 | 6B | 65 | 1.30E-04 | 7.57 |
| Pb | E1 | BS00022772_51 | 6B | 65 | 3.75E-04 | 6.50 |
| Pb | E1 | BS00094322_51 | 6B | 67 | 1.62E-04 | 7.33 |
| Pb | E1 | BS00106588_51 | 6B | 67 | 1.98E-04 | 7.14 |
| Pb | E1 | BobWhite_c27318_380 | 6B | 67 | 6.06E-05 | 8.35 |
| Pb | E1 | Excalibur_c6416_1712 | 6B | 67 | 6.07E-05 | 8.35 |
| Pb | E1 | BobWhite_c36415_378 | 6B | 67 | 9.21E-05 | 7.90 |
| Pb | E1 | GENE-0363_168 | 6B | 67 | 1.98E-04 | 7.14 |
| Pb | E1 | IACX203 | 6B | 67 | 3.00E-05 | 9.12 |
| Pb | E1 | Kukri_c63145_262 | 6B | 67 | 1.17E-04 | 7.66 |
| Pb | E1 | BobWhite_rep_c63804_353 | 6B | 67 | 1.16E-04 | 7.69 |
| Pb | E1 | BS00064820_51 | 6B | 67 | 1.98E-04 | 7.14 |
| Pb | E2 | RAC875_c3001_1236 | 1B | 148 | 9.02E-04 | 5.71 |
| Pb | E2 | RAC875_c3001_808 | 1B | 148 | 9.02E-04 | 5.71 |
| Pb | E2 | Tdurum_contig10354_170 | 1B | 148 | 9.02E-04 | 5.71 |
| Pb | E2 | BS00021877_51 | 1B | 155 | 9.02E-04 | 5.71 |
| Pb | E2 | BS00083626_51 | 2B | 173 | 9.90E-05 | 7.94 |
| Pb | E2 | IAAV5729 | 3A | 61 | 8.92E-04 | 5.72 |
| Pb | E2 | RAC875_c47976_291 | 3A | 61 | 8.92E-04 | 5.72 |
| Pb | E2 | RFL_Contig4881_137 | 4B | 40 | 9.98E-04 | 5.62 |
| Pb | E2 | RAC875_c44407_364 | 5A | 63 | 5.59E-04 | 6.19 |
| Pb | E2 | D_contig07330_330 | 7D | 108 | 7.54E-04 | 6.05 |
| Pb | E2 | Ku_c884_791 | 7D | 109 | 4.85E-04 | 6.38 |
| Pb | E3 | Excalibur_c25353_1171 | 1A | 106 | 7.94E-04 | 5.95 |
| Pb | E3 | Ex_c4206_502 | 1B | 108 | 6.88E-05 | 8.48 |
| Pb | E3 | tplb0042o10_1533 | 3B | 55 | 7.65E-04 | 6.01 |
| Pb | E3 | wsnp_Ex_c123_244117 | 3B | 69 | 6.55E-04 | 6.16 |
| Pb | E3 | wsnp_Ex_c14462_22457559 | 3B | 69 | 2.49E-04 | 7.15 |
| Pb | E3 | wsnp_Ex_c2330_4366134 | 3B | 69 | 6.64E-04 | 6.15 |
| Pb | E3 | wsnp_Ex_c4769_8510104 | 3B | 69 | 6.55E-04 | 6.16 |
| Pb | E3 | wsnp_Ku_c8722_14766699 | 3B | 69 | 6.55E-04 | 6.16 |
| Pb | E3 | IACX5407 | 3B | 69 | 2.44E-04 | 7.18 |
| Pb | E3 | RAC875_c100413_60 | 3B | 69 | 3.11E-04 | 6.91 |
| Pb | E3 | RAC875_c17884_616 | 3B | 69 | 6.55E-04 | 6.16 |
| Pb | E3 | RAC875_c40919_1075 | 3B | 69 | 6.64E-04 | 6.15 |
| Pb | E3 | TA002822-1591 | 3B | 69 | 6.89E-04 | 6.10 |
| Pb | E3 | Tdurum_contig20950_359 | 3B | 69 | 6.82E-04 | 6.11 |
| Pb | E3 | BS00105995_51 | 3B | 69 | 2.44E-04 | 7.18 |
| Pb | E3 | RFL_Contig5418_347 | 3B | 70 | 1.44E-04 | 7.70 |
| Pb | E3 | Tdurum_contig48760_112 | 5A | 69 | 7.77E-05 | 8.37 |
| Pb | E3 | Excalibur_c1215_334 | 7A | 127 | 5.29E-05 | 8.88 |
| Pb | E3 | BS00064367_51 | 7B | 117 | 3.51E-04 | 6.79 |
| Pb | E3 | BS00109317_51 | 7B | 131 | 6.71E-04 | 6.17 |
| Pb | E3 | Ku_c6550_1698 | 7B | 131 | 6.71E-04 | 6.17 |
| Pb | E4 | Kukri_c105601_74 | 1B | 51 | 2.52E-04 | 7.18 |
| Pb | E4 | RAC875_c25101_644 | 1B | 51 | 2.52E-04 | 7.18 |
| Pb | E4 | wsnp_Ku_c11987_19472688 | 1B | 57 | 2.52E-04 | 7.18 |
| Pb | E4 | Kukri_c18006_1568 | 1B | 57 | 2.52E-04 | 7.18 |
| Pb | E4 | RAC875_c8271_1352 | 1B | 57 | 2.52E-04 | 7.18 |
| Pb | E4 | RAC875_c8271_1469 | 1B | 57 | 2.52E-04 | 7.18 |
| Pb | E4 | RAC875_c8271_887 | 1B | 57 | 2.52E-04 | 7.18 |
| Pb | E4 | RAC875_rep_c96733_369 | 1B | 57 | 2.52E-04 | 7.18 |
| Pb | E4 | BS00022255_51 | 1B | 57 | 1.28E-04 | 7.90 |
| Pb | E4 | wsnp_Ku_c11987_19473636 | 1B | 58 | 2.47E-04 | 7.21 |
| Pb | E4 | D_contig25392_201 | 1B | 61 | 1.28E-04 | 7.90 |
| Pb | E4 | Kukri_c23300_267 | 1B | 61 | 2.52E-04 | 7.18 |
| Pb | E4 | BS00022176_51 | 1B | 108 | 4.06E-04 | 6.69 |
| Pb | E4 | RAC875_c9594_1289 | 1D | 65 | 5.83E-04 | 6.37 |
| Pb | E4 | Kukri_rep_c72412_856 | 2A | 79 | 7.61E-04 | 6.04 |
| Pb | E4 | Kukri_c57078_153 | 2A | 104 | 1.97E-04 | 7.46 |
| Pb | E4 | Kukri_c209_999 | 2A | 143 | 1.03E-04 | 8.30 |
| Pb | E4 | BS00091763_51 | 2A | 167 | 5.30E-04 | 6.41 |
| Pb | E4 | wsnp_Ex_rep_c108004_91402649 | 2A | 168 | 2.24E-04 | 7.31 |
| Pb | E4 | GENE-0762_808 | 2A | 168 | 1.51E-04 | 7.72 |
| Pb | E4 | wsnp_Ex_c14162_22093694 | 2B | 85 | 3.44E-04 | 7.49 |
| Pb | E4 | RAC875_c17479_359 | 3A | 93 | 8.02E-04 | 5.99 |
| Pb | E4 | CAP12_c1787_110 | 3A | 95 | 3.64E-04 | 6.80 |
| Pb | E4 | BS00022424_51 | 3A | 141 | 5.65E-05 | 8.76 |
| Pb | E4 | Kukri_c77040_87 | 4A | 91 | 1.60E-04 | 7.66 |
| Pb | E4 | IAAV1943 | 4A | 144 | 6.57E-04 | 6.20 |
| Pb | E4 | Tdurum_contig61465_781 | 4B | 61 | 2.53E-04 | 7.18 |
| Pb | E4 | BobWhite_c44324_227 | 5A | 16 | 8.77E-04 | 5.90 |
| Pb | E4 | wsnp_Ex_c28908_37989067 | 5A | 27 | 1.10E-05 | 10.53 |
| Pb | E4 | wsnp_Ku_c1254_2498515 | 5A | 27 | 3.89E-05 | 9.16 |
| Pb | E4 | BS00033185_51 | 5B | 174 | 1.47E-04 | 7.75 |
| Pb | E4 | CAP7_c8713_356 | 5B | 183 | 1.29E-04 | 7.88 |
| Pb | E4 | BS00067074_51 | 5B | 183 | 1.29E-04 | 7.88 |
| Pb | E4 | BobWhite_c46416_247 | 5B | 183 | 3.24E-04 | 6.92 |
| Pb | E4 | Kukri_c4594_825 | 5B | 183 | 3.24E-04 | 6.92 |
| Pb | E4 | RAC875_c1035_65 | 5B | 183 | 1.29E-04 | 7.88 |
| Pb | E4 | Tdurum_contig60189_310 | 5B | 183 | 1.30E-04 | 7.89 |
| Pb | E4 | BS00068775_51 | 5B | 184 | 1.65E-04 | 7.72 |
| Pb | E4 | RAC875_rep_c74271_414 | 5B | 184 | 1.89E-04 | 7.48 |
| Pb | E4 | BobWhite_rep_c65775_338 | 6B | 0 | 7.94E-04 | 6.12 |
| Pb | E4 | CAP7_c3697_87 | 6B | 86 | 1.31E-04 | 7.87 |
| Pb | E4 | Tdurum_contig68217_361 | 6B | 86 | 1.31E-04 | 7.87 |
| Pb | E4 | BobWhite_c17095_237 | 7A | 136 | 3.65E-04 | 6.80 |
| Pb | E4 | RAC875_c57326_85 | 7B | 134 | 8.71E-05 | 8.30 |
| Pb | E4 | wsnp_Ex_c8400_14157060 | 7B | 134 | 8.71E-05 | 8.30 |
| Pb | E4 | wsnp_JD_c17128_16056425 | 7B | 143 | 4.25E-04 | 6.64 |
| Pb | E4 | Excalibur_c19455_3496 | 7B | 163 | 9.11E-06 | 10.81 |
| Pb | E4 | Excalibur_c11062_582 | 7B | 171 | 1.90E-06 | 12.47 |
| Pb | E4 | Excalibur_c25090_830 | 7B | 171 | 8.11E-07 | 13.43 |
| Pb | E4 | RAC875_c34939_963 | 7B | 171 | 3.32E-04 | 6.90 |
| Pb | E4 | RAC875_rep_c110526_229 | 7B | 171 | 1.90E-06 | 12.47 |

E1: 2014DZ; E2: 2015TA; E3: 2016TA; E4: 2017TA.

Table S5 Candidate genes predication and mainly functions of important MATs loci associated with mineral elements

| Trait | MAT locus | Chr. | Candidate genes and physical position | Mainly Function | Biological process/Expression | Species |
| --- | --- | --- | --- | --- | --- | --- |
| Ca, Zn | BS00057451_51 | 3B | [TraesCS3B02G307600](http://plants.ensembl.org/Triticum_aestivum/Gene/Summary?db=core;g=TraesCS3B02G307600;tl=dA4oed2JnA1VDG5b-20387363-1611780563).1  493655348..493657938 | - [dihydroorotase activity](https://www.ebi.ac.uk/QuickGO/term/GO:0004151) - **"Phylogenetic-based propagation of functional annotations within the Gene Ontology consortium."** [**Gaudet P.**](https://www.uniprot.org/uniprot/?query=author:)**,** [**Livstone M.S.**](https://www.uniprot.org/uniprot/?query=author:)**,** [**Lewis S.E.**](https://www.uniprot.org/uniprot/?query=author:)**,** [**Thomas P.D.**](https://www.uniprot.org/uniprot/?query=author:) [**Brief Bioinform 12:449-462(2011)**](http://dx.doi.org/10.1093/bib/bbr042) **[**[**PubMed**](https://pubmed.ncbi.nlm.nih.gov/21873635)**] [**[**Europe PMC**](https://europepmc.org/abstract/MED/21873635)**] [**[**Abstract**](https://www.uniprot.org/citations/21873635)**]** - [**metal ion binding**](https://www.ebi.ac.uk/QuickGO/term/GO:0046872) | - ['de novo' pyrimidine nucleobase biosynthetic process](https://www.ebi.ac.uk/QuickGO/term/GO:0006207)  - Inferred from biological aspect of ancestor^i^] - ['de novo' UMP biosynthetic process](https://www.ebi.ac.uk/QuickGO/term/GO:0044205) - [pyrimidine nucleotide biosynthetic process](https://www.ebi.ac.uk/QuickGO/term/GO:0006221) | Triticum aestivum |
|  |  |  |  | - [**metal ion binding**](https://www.ebi.ac.uk/QuickGO/term/GO:0046872) - [ubiquitin protein ligase activity](https://www.ebi.ac.uk/QuickGO/term/GO:0061630) - [ubiquitin-protein transferase activity](https://www.ebi.ac.uk/QuickGO/term/GO:0004842) - [ubiquitin-ubiquitin ligase activity](https://www.ebi.ac.uk/QuickGO/term/GO:0034450) - [RNA binding](https://www.ebi.ac.uk/QuickGO/term/GO:0003723) | - [positive regulation of ubiquitin-dependent protein catabolic process](https://www.ebi.ac.uk/QuickGO/term/GO:2000060) - [protein catabolic process](https://www.ebi.ac.uk/QuickGO/term/GO:0030163) - [protein ubiquitination](https://www.ebi.ac.uk/QuickGO/term/GO:0016567) - [proteolysis involved in cellular protein catabolic process](https://www.ebi.ac.uk/QuickGO/term/GO:0051603) - [ubiquitin-dependent protein catabolic process](https://www.ebi.ac.uk/QuickGO/term/GO:0006511) - RNA processing | Arabidopsis thaliana |
|  |  |  |  | - [ATP binding](https://www.ebi.ac.uk/QuickGO/term/GO:0005524) - [protein kinase activity](https://www.ebi.ac.uk/QuickGO/term/GO:0004672) - [protein serine/threonine kinase activity](https://www.ebi.ac.uk/QuickGO/term/GO:0004674) |  | Arabidopsis thaliana |
|  |  |  |  | - [**calcium ion binding**](https://www.ebi.ac.uk/QuickGO/term/GO:0005509) |  | Triticum urartu |
|  |  |  |  | - [DNA binding](https://www.ebi.ac.uk/QuickGO/term/GO:0003677) - [DNA-directed 5'-3' RNA polymerase activity](https://www.ebi.ac.uk/QuickGO/term/GO:0003899) - [**metal ion binding**](https://www.ebi.ac.uk/QuickGO/term/GO:0046872) - [ribonucleoside binding](https://www.ebi.ac.uk/QuickGO/term/GO:0032549) | - [megagametogenesis](https://www.ebi.ac.uk/QuickGO/term/GO:0009561) - [transcription, DNA-templated](https://www.ebi.ac.uk/QuickGO/term/GO:0006351) | Oryza sativa Indica Group |
|  |  |  |  | - [heme binding](https://www.ebi.ac.uk/QuickGO/term/GO:0020037) - [**metal ion binding**](https://www.ebi.ac.uk/QuickGO/term/GO:0046872) - [peroxidase activity](https://www.ebi.ac.uk/QuickGO/term/GO:0004601) | - [hydrogen peroxide catabolic process](https://www.ebi.ac.uk/QuickGO/term/GO:0042744) - [response to oxidative stress](https://www.ebi.ac.uk/QuickGO/term/GO:0006979) | Hordeum vulgare；  Oryza rufipogon |
|  |  |  |  | - [DNA binding](https://www.ebi.ac.uk/QuickGO/term/GO:0003677) - [DNA-directed 5'-3' RNA polymerase activity](https://www.ebi.ac.uk/QuickGO/term/GO:0003899) - [**metal ion binding**](https://www.ebi.ac.uk/QuickGO/term/GO:0046872) - [ribonucleoside binding](https://www.ebi.ac.uk/QuickGO/term/GO:0032549) | - [megagametogenesis](https://www.ebi.ac.uk/QuickGO/term/GO:0009561) - [transcription, DNA-templated](https://www.ebi.ac.uk/QuickGO/term/GO:0006351) | Oryza rufipogon |
| Ca, Zn | Excalibur_c41752_392 | 3B | TraesCS3B02G307400 493648449..493653177 | - [RNA binding](https://www.ebi.ac.uk/QuickGO/term/GO:0003723) |  | Triticum aestivum;  Oryza sativa Japonica Group; Oryza sativa Indica Group |
|  |  |  |  | - [**metal ion binding**](https://www.ebi.ac.uk/QuickGO/term/GO:0046872) |  | Oryza sativa Japonica Group; Oryza sativa Indica Group |
|  |  |  |  | - [ATP binding](https://www.ebi.ac.uk/QuickGO/term/GO:0005524) - [protein kinase activity](https://www.ebi.ac.uk/QuickGO/term/GO:0004672) |  | Oryza sativa Japonica Group  Oryza sativa Indica Group |
|  |  |  |  | - [ATPase activity](https://www.ebi.ac.uk/QuickGO/term/GO:0016887) - [ATP binding](https://www.ebi.ac.uk/QuickGO/term/GO:0005524) - [protein self-association](https://www.ebi.ac.uk/QuickGO/term/GO:0043621) | - [posttranslational protein targeting to endoplasmic reticulum membrane](https://www.ebi.ac.uk/QuickGO/term/GO:0006620) - [protein insertion into ER membrane](https://www.ebi.ac.uk/QuickGO/term/GO:0045048) - [root hair elongation](https://www.ebi.ac.uk/QuickGO/term/GO:0048767) | Arabidopsis thaliana |
|  |  |  |  | - [**metal ion binding**](https://www.ebi.ac.uk/QuickGO/term/GO:0046872) - [oxidoreductase activity](https://www.ebi.ac.uk/QuickGO/term/GO:0016491) | - [response to karrikin](https://www.ebi.ac.uk/QuickGO/term/GO:0080167) | Arabidopsis thaliana |
|  |  |  |  | - [ATP binding](https://www.ebi.ac.uk/QuickGO/term/GO:0005524) - [protein kinase activity](https://www.ebi.ac.uk/QuickGO/term/GO:0004672) |  | Oryza rufipogon |
|  |  |  |  | - [**metal ion binding**](https://www.ebi.ac.uk/QuickGO/term/GO:0046872) |  | Oryza rufipogon |
| Se | Excalibur_rep_c93332_58 | 3D | TraesCS3D02G201900 242599912..242602612 | - [polyubiquitin modification-dependent protein binding](https://www.ebi.ac.uk/QuickGO/term/GO:0031593) - Ubiquitin fusion degradation protein 1-like protein | - [ER-associated misfolded protein catabolic process](https://www.ebi.ac.uk/QuickGO/term/GO:0071712) - [ubiquitin-dependent ERAD pathway](https://www.ebi.ac.uk/QuickGO/term/GO:0030433) | Triticum aestivum |
|  |  |  |  | - [cellulose synthase (UDP-forming) activity](https://www.ebi.ac.uk/QuickGO/term/GO:0016760) - [cellulose synthase activity](https://www.ebi.ac.uk/QuickGO/term/GO:0016759) - [**metal ion binding**](https://www.ebi.ac.uk/QuickGO/term/GO:0046872) | - [cellulose biosynthetic process](https://www.ebi.ac.uk/QuickGO/term/GO:0030244) - [cell wall organization](https://www.ebi.ac.uk/QuickGO/term/GO:0071555) - [cortical microtubule organization](https://www.ebi.ac.uk/QuickGO/term/GO:0043622) - [multidimensional cell growth](https://www.ebi.ac.uk/QuickGO/term/GO:0009825) - [plant-type primary cell wall biogenesis](https://www.ebi.ac.uk/QuickGO/term/GO:0009833) - ExpressionAtlasi Q94JQ6﻿, baseline and differential - Genevisiblei Q94JQ6﻿, AT | Arabidopsis thaliana |
|  |  |  |  | - [DNA-binding transcription factor activity](https://www.ebi.ac.uk/QuickGO/term/GO:0003700) - [**metal ion binding**](https://www.ebi.ac.uk/QuickGO/term/GO:0046872) - [sequence-specific DNA binding](https://www.ebi.ac.uk/QuickGO/term/GO:0043565) - [transcription regulatory region sequence-specific DNA binding](https://www.ebi.ac.uk/QuickGO/term/GO:0000976) | - [asymmetric cell division](https://www.ebi.ac.uk/QuickGO/term/GO:0008356) - [gibberellic acid mediated signaling pathway](https://www.ebi.ac.uk/QuickGO/term/GO:0009740) - [positive regulation of gibberellic acid mediated signaling pathway](https://www.ebi.ac.uk/QuickGO/term/GO:0009939) - [positive regulation of transcription, DNA-templated](https://www.ebi.ac.uk/QuickGO/term/GO:0045893) - [protein localization to nucleus](https://www.ebi.ac.uk/QuickGO/term/GO:0034504) | Arabidopsis thaliana |
|  |  |  |  | - [ATP binding](https://www.ebi.ac.uk/QuickGO/term/GO:0005524) - [protein serine/threonine kinase activity](https://www.ebi.ac.uk/QuickGO/term/GO:0004674) | - [protein phosphorylation](https://www.ebi.ac.uk/QuickGO/term/GO:0006468) | Arabidopsis thaliana |
|  |  |  |  | - [hydrolase activity](https://www.ebi.ac.uk/QuickGO/term/GO:0016787) - [**metal ion binding**](https://www.ebi.ac.uk/QuickGO/term/GO:0046872) | - ExpressionAtlasi A0A178WDC8﻿, baseline and differential | Arabidopsis thaliana |
|  |  |  |  | - [dipeptidyl-peptidase activity](https://www.ebi.ac.uk/QuickGO/term/GO:0008239) - [**metal ion binding**](https://www.ebi.ac.uk/QuickGO/term/GO:0046872) | - ExpressionAtlasi Q8L831﻿, baseline and differential - Genevisiblei Q8L831﻿, AT | Arabidopsis thaliana |
|  |  |  |  | - [ATP binding](https://www.ebi.ac.uk/QuickGO/term/GO:0005524) - [ATP-dependent peptidase activity](https://www.ebi.ac.uk/QuickGO/term/GO:0004176) - [metalloendopeptidase activity](https://www.ebi.ac.uk/QuickGO/term/GO:0004222) - [**zinc ion binding**](https://www.ebi.ac.uk/QuickGO/term/GO:0008270) | - [cristae formation](https://www.ebi.ac.uk/QuickGO/term/GO:0042407) - [mitochondrial fusion](https://www.ebi.ac.uk/QuickGO/term/GO:0008053) - [mitochondrial protein processing](https://www.ebi.ac.uk/QuickGO/term/GO:0034982) - [protein-containing complex assembly](https://www.ebi.ac.uk/QuickGO/term/GO:0065003) - ExpressionAtlasi Q8VZI8﻿, baseline and differential - Genevisiblei Q8VZI8﻿, AT | Arabidopsis thaliana |
|  |  |  |  | - [calcium ion binding](https://www.ebi.ac.uk/QuickGO/term/GO:0005509) | - [regulation of transcription, DNA-templated](https://www.ebi.ac.uk/QuickGO/term/GO:0006355) - [response to salt stress](https://www.ebi.ac.uk/QuickGO/term/GO:0009651) - [sodium ion homeostasis](https://www.ebi.ac.uk/QuickGO/term/GO:0055078) | Oryza rufipogon  Oryza sativa Indica |
| Ca | Kukri_c41797_393 | 5A | [TraesCS5A02G256700](http://plants.ensembl.org/Triticum_aestivum/Gene/Summary?db=core;g=TraesCS5A02G256700;tl=OCGBdYCXR6AIFnMd-20365822-1550653137).1  472274579..472278029  TraesCS5A01G256800.1  472283478..472286522 | - [**ribosomal small subunit biogenesis**](https://www.ebi.ac.uk/QuickGO/term/GO:0042274) - [**ribosomal small subunit export from nucleus**](https://www.ebi.ac.uk/QuickGO/term/GO:0000056) - Protein LTV1 - Mechanosensitive ion channel |  | Triticum aestivum |
|  |  |  |  | - [**microtubule binding**](https://www.ebi.ac.uk/QuickGO/term/GO:0008017) - [**microtubule plus end polymerase**](https://www.ebi.ac.uk/QuickGO/term/GO:0061863) | - [establishment or maintenance of microtubule cytoskeleton polarity](https://www.ebi.ac.uk/QuickGO/term/GO:0030951) - [**microtubule polymerization**](https://www.ebi.ac.uk/QuickGO/term/GO:0046785) - [mitotic spindle organization](https://www.ebi.ac.uk/QuickGO/term/GO:0007052) | Oryza sativa Japonica Group |
|  |  |  |  | - [oxidoreductase activity](https://www.ebi.ac.uk/QuickGO/term/GO:0016491) | - [indolebutyric acid metabolic process](https://www.ebi.ac.uk/QuickGO/term/GO:0080024) - [indolebutyric acid metabolic process](https://www.ebi.ac.uk/QuickGO/term/GO:0080024) - [**root hair elongation**](https://www.ebi.ac.uk/QuickGO/term/GO:0048767) | Oryza sativa Japonica Group |
|  |  |  |  | - [ATP binding](https://www.ebi.ac.uk/QuickGO/term/GO:0005524) - [**protein kinase activity**](https://www.ebi.ac.uk/QuickGO/term/GO:0004672) |  | Oryza rufipogon |
|  |  |  |  | - [metal ion binding](https://www.ebi.ac.uk/QuickGO/term/GO:0046872) | - [cellular response to hypoxia](https://www.ebi.ac.uk/QuickGO/term/GO:0071456) | Arabidopsis thaliana |
|  |  |  |  | - [calcium ion binding](https://www.ebi.ac.uk/QuickGO/term/GO:0005509) - [N-acylphosphatidylethanolamine-specific phospholipase D activity](https://www.ebi.ac.uk/QuickGO/term/GO:0070290) - [phospholipase D activity](https://www.ebi.ac.uk/QuickGO/term/GO:0004630) | - [lipid catabolic process](https://www.ebi.ac.uk/QuickGO/term/GO:0016042) - [phosphatidylcholine metabolic process](https://www.ebi.ac.uk/QuickGO/term/GO:0046470) - [phosphatidic acid metabolic process](https://www.ebi.ac.uk/QuickGO/term/GO:0046473) | Arabidopsis thaliana |
|  |  |  |  | - [ATPase-coupled cation transmembrane transporter activity](https://www.ebi.ac.uk/QuickGO/term/GO:0019829) - [ATP binding](https://www.ebi.ac.uk/QuickGO/term/GO:0005524) - [calcium transmembrane transporter activity, phosphorylative mechanism](https://www.ebi.ac.uk/QuickGO/term/GO:0005388) - [calmodulin binding](https://www.ebi.ac.uk/QuickGO/term/GO:0005516) - [metal ion binding](https://www.ebi.ac.uk/QuickGO/term/GO:0046872) | - [anion homeostasis](https://www.ebi.ac.uk/QuickGO/term/GO:0055081) - [defense response to bacterium](https://www.ebi.ac.uk/QuickGO/term/GO:0042742) - [negative regulation of programmed cell death](https://www.ebi.ac.uk/QuickGO/term/GO:0043069) | Arabidopsis thaliana |
|  |  |  |  | - [damaged DNA binding](https://www.ebi.ac.uk/QuickGO/term/GO:0003684) - [DNA-directed DNA polymerase activity](https://www.ebi.ac.uk/QuickGO/term/GO:0003887) - [iron-sulfur cluster binding](https://www.ebi.ac.uk/QuickGO/term/GO:0051536) - [metal ion binding](https://www.ebi.ac.uk/QuickGO/term/GO:0046872) | - [cyclic nucleotide biosynthetic process](https://www.ebi.ac.uk/QuickGO/term/GO:0009190) - [DNA repair](https://www.ebi.ac.uk/QuickGO/term/GO:0006281) - [intracellular signal transduction](https://www.ebi.ac.uk/QuickGO/term/GO:0035556) | Arabidopsis thaliana |
|  |  |  |  | - [hydrolase activity](https://www.ebi.ac.uk/QuickGO/term/GO:0016787) - [metal ion binding](https://www.ebi.ac.uk/QuickGO/term/GO:0046872) | - [nonphotochemical quenching](https://www.ebi.ac.uk/QuickGO/term/GO:0010196) | Arabidopsis thaliana |
|  |  |  |  | - [metal ion binding](https://www.ebi.ac.uk/QuickGO/term/GO:0046872) | - [cellular response to cold](https://www.ebi.ac.uk/QuickGO/term/GO:0070417) - [response to cold](https://www.ebi.ac.uk/QuickGO/term/GO:0009409) | Arabidopsis thaliana |
|  |  |  |  | - [alpha-mannosidase activity](https://www.ebi.ac.uk/QuickGO/term/GO:0004559) - [carbohydrate binding](https://www.ebi.ac.uk/QuickGO/term/GO:0030246) - [metal ion binding](https://www.ebi.ac.uk/QuickGO/term/GO:0046872) | - [mannose metabolic process](https://www.ebi.ac.uk/QuickGO/term/GO:0006013) | Arabidopsis thaliana |
| Ca | RFL_Contig2187_1025 | 5A | [TraesCS5A02G257000](http://plants.ensembl.org/Triticum_aestivum/Gene/Summary?db=core;g=TraesCS5A02G257000;tl=04BYVMRSIhxu3hqb-20366184-1552437559).1  472343789..472347557 | - WPP domain-interacting protein 1 |  | Triticum aestivum |
|  |  |  |  | - [serine-type carboxypeptidase activity](https://www.ebi.ac.uk/QuickGO/term/GO:0004185) - [transferase activity, transferring acyl groups other than amino-acyl groups](https://www.ebi.ac.uk/QuickGO/term/GO:0016747) | - [secondary metabolic process](https://www.ebi.ac.uk/QuickGO/term/GO:0019748) | Oryza sativa Japonica Group |
|  |  |  |  | - [ATP binding](https://www.ebi.ac.uk/QuickGO/term/GO:0005524) - [kinase activity](https://www.ebi.ac.uk/QuickGO/term/GO:0016301) - [protein serine/threonine kinase activity](https://www.ebi.ac.uk/QuickGO/term/GO:0004674) | - [auxin-activated signaling pathway](https://www.ebi.ac.uk/QuickGO/term/GO:0009734) - [cotyledon development](https://www.ebi.ac.uk/QuickGO/term/GO:0048825) - [protein phosphorylation](https://www.ebi.ac.uk/QuickGO/term/GO:0006468) | Arabidopsis thaliana |
|  |  |  |  | - [hydrolase activity](https://www.ebi.ac.uk/QuickGO/term/GO:0016787) - [ubiquitin-protein transferase activity](https://www.ebi.ac.uk/QuickGO/term/GO:0004842) - [**zinc ion binding**](https://www.ebi.ac.uk/QuickGO/term/GO:0008270) - [hydrolase activity](https://www.ebi.ac.uk/QuickGO/term/GO:0016787) | - [regulation of seed germination](https://www.ebi.ac.uk/QuickGO/term/GO:0010029) | Arabidopsis thaliana |
|  |  |  |  | - [zinc ion transmembrane transporter activity](https://www.ebi.ac.uk/QuickGO/term/GO:0005385) | - [zinc ion transmembrane transport](https://www.ebi.ac.uk/QuickGO/term/GO:0071577) | Arabidopsis thaliana |
|  |  |  |  | - [metal ion binding](https://www.ebi.ac.uk/QuickGO/term/GO:0046872) - [transferase activity, transferring glycosyl groups](https://www.ebi.ac.uk/QuickGO/term/GO:0016757) | - [shoot apical meristem development](https://www.ebi.ac.uk/QuickGO/term/GO:1902182) | Arabidopsis thaliana |
|  |  |  |  | - [ADP binding](https://www.ebi.ac.uk/QuickGO/term/GO:0043531) - [ATP binding](https://www.ebi.ac.uk/QuickGO/term/GO:0005524) | - [defense response](https://www.ebi.ac.uk/QuickGO/term/GO:0006952) | Oryza rufipogon |
|  |  |  |  | - [acid phosphatase activity](https://www.ebi.ac.uk/QuickGO/term/GO:0003993) - [metal ion binding](https://www.ebi.ac.uk/QuickGO/term/GO:0046872) |  | Oryza rufipogon |
| Zn | BobWhite_c7907_657 | 7B | TraesCS7B02G142200181034410..181038770 | - [DNA binding](https://www.ebi.ac.uk/QuickGO/term/GO:0003677) - [metal ion binding](https://www.ebi.ac.uk/QuickGO/term/GO:0046872) | Squamosa promoter-binding protein | Triticum aestivum |
| Fe | Excalibur_c6326_77 | 6B | TraesCS6B02G029300 17703175..17704083 | - **[calcium-dependent](https://www.ebi.ac.uk/QuickGO/term/GO:0005544)** [**[calcium ion binding](https://www.ebi.ac.uk/QuickGO/term/GO:0005544)**](https://www.ebi.ac.uk/QuickGO/term/GO:0005509) - [phospholipid binding](https://www.ebi.ac.uk/QuickGO/term/GO:0005544) |  | Arabidopsis thaliana |
|  |  |  |  | - [ATP binding](https://www.ebi.ac.uk/QuickGO/term/GO:0005524) - [ATP citrate synthase activity](https://www.ebi.ac.uk/QuickGO/term/GO:0003878) | - [acetyl-CoA biosynthetic](https://www.ebi.ac.uk/QuickGO/term/GO:0006085) [[lipid metabolic process](https://www.ebi.ac.uk/QuickGO/term/GO:0006085)](https://www.ebi.ac.uk/QuickGO/term/GO:0006629) - [process](https://www.ebi.ac.uk/QuickGO/term/GO:0006085) | Arabidopsis thaliana |
|  |  |  |  | - [DNA-binding transcription factor activity](https://www.ebi.ac.uk/QuickGO/term/GO:0003700) - [transcription coactivator activity](https://www.ebi.ac.uk/QuickGO/term/GO:0003713) - [DNA binding](https://www.ebi.ac.uk/QuickGO/term/GO:0003677) | - [cellular response to hypoxia](https://www.ebi.ac.uk/QuickGO/term/GO:0071456) - [defense response to virus](https://www.ebi.ac.uk/QuickGO/term/GO:0051607) - [positive regulation of defense](https://www.ebi.ac.uk/QuickGO/term/GO:0002230) [[response to molecule of bacterial origin](https://www.ebi.ac.uk/QuickGO/term/GO:0002230)](https://www.ebi.ac.uk/QuickGO/term/GO:0002237) - [response to virus by host](https://www.ebi.ac.uk/QuickGO/term/GO:0002230) - [response to virus](https://www.ebi.ac.uk/QuickGO/term/GO:0009615) - [viral process](https://www.ebi.ac.uk/QuickGO/term/GO:0016032) | Arabidopsis thaliana |
|  |  |  |  | - [**iron ion binding**](https://www.ebi.ac.uk/QuickGO/term/GO:0005506) - [heme binding](https://www.ebi.ac.uk/QuickGO/term/GO:0020037) - [monooxygenase activity](https://www.ebi.ac.uk/QuickGO/term/GO:0004497) - [oxidoreductase activity, acting on paired donors, with incorporation or reduction of molecular oxygen](https://www.ebi.ac.uk/QuickGO/term/GO:0016705) |  | Oryza rufipogon |
| Fe, Pb | Excalibur_c19455_3496 | 7B | [TraesCS7B02G480300](https://plants.ensembl.org/Triticum_aestivum/Gene/Summary?db=core;g=TraesCS7B02G480300;tl=Rf30gjXpHk0GKXvx-20376673-1605566720)  734259844..734274042 | - [1,3-beta-D-glucan synthase activity](https://www.ebi.ac.uk/QuickGO/term/GO:0003843) - [glucosyltransferase activity](https://www.ebi.ac.uk/QuickGO/term/GO:0046527) - Callose synthase | - [(1->3)-beta-D-glucan biosynthetic process](https://www.ebi.ac.uk/QuickGO/term/GO:0006075) - [cell wall organization](https://www.ebi.ac.uk/QuickGO/term/GO:0071555) - [regulation of cell shape](https://www.ebi.ac.uk/QuickGO/term/GO:0008360) | Triticum aestivum；Oryza sativa Indica Group；Triticum urartu；Oryza rufipogon；Oryza sativa Japonica Group |
|  |  |  |  | - [heme binding](https://www.ebi.ac.uk/QuickGO/term/GO:0020037) - [**iron ion binding**](https://www.ebi.ac.uk/QuickGO/term/GO:0005506) - [monooxygenase activity](https://www.ebi.ac.uk/QuickGO/term/GO:0004497) - [oxidoreductase activity, acting on paired donors, with incorporation or reduction of molecular oxygen](https://www.ebi.ac.uk/QuickGO/term/GO:0016705) | - A0A287RGQ6, A0A287RGI4, A0A287RGH9 | Hordeum vulgare |
|  |  |  |  | - [calcium ion binding](https://www.ebi.ac.uk/QuickGO/term/GO:0005509) | - [response to symbiotic fungus](https://www.ebi.ac.uk/QuickGO/term/GO:0009610) | Oryza sativa Indica Group；Oryza sativa Japonica Group |
|  |  |  |  | - [iron ion transmembrane transporter activity](https://www.ebi.ac.uk/QuickGO/term/GO:0005381) - [zinc ion transmembrane transporter activity](https://www.ebi.ac.uk/QuickGO/term/GO:0005385) | - [iron ion homeostasis](https://www.ebi.ac.uk/QuickGO/term/GO:0055072) - [iron ion transport](https://www.ebi.ac.uk/QuickGO/term/GO:0006826) - [response to nematode](https://www.ebi.ac.uk/QuickGO/term/GO:0009624) - [zinc ion transmembrane transport](https://www.ebi.ac.uk/QuickGO/term/GO:0071577) - [zinc ion transport](https://www.ebi.ac.uk/QuickGO/term/GO:0006829) | Arabidopsis thaliana |
|  |  |  |  | - [metal ion binding](https://www.ebi.ac.uk/QuickGO/term/GO:0046872) | - [metal ion transport](https://www.ebi.ac.uk/QuickGO/term/GO:0030001) | Arabidopsis thaliana |
|  |  |  |  | - [ATP binding](https://www.ebi.ac.uk/QuickGO/term/GO:0005524) - [kinase activity](https://www.ebi.ac.uk/QuickGO/term/GO:0016301) - [metal ion binding](https://www.ebi.ac.uk/QuickGO/term/GO:0046872) - [protein serine/threonine kinase activity](https://www.ebi.ac.uk/QuickGO/term/GO:0004674) | - [intracellular signal transduction](https://www.ebi.ac.uk/QuickGO/term/GO:0035556) - [peptidyl-serine phosphorylation](https://www.ebi.ac.uk/QuickGO/term/GO:0018105) - [protein autophosphorylation](https://www.ebi.ac.uk/QuickGO/term/GO:0046777) - [regulation of growth](https://www.ebi.ac.uk/QuickGO/term/GO:0040008) | Arabidopsis thaliana |
|  |  |  |  | - [metal ion transmembrane transporter activity](https://www.ebi.ac.uk/QuickGO/term/GO:0046873) |  | Arabidopsis thaliana |
|  |  |  |  | - [zinc ion transmembrane transporter activity](https://www.ebi.ac.uk/QuickGO/term/GO:0005385) | - [zinc ion transmembrane transport](https://www.ebi.ac.uk/QuickGO/term/GO:0071577) | Arabidopsis thaliana |
|  |  |  |  | - [ATP binding](https://www.ebi.ac.uk/QuickGO/term/GO:0005524) - [ATP-dependent peptidase activity](https://www.ebi.ac.uk/QuickGO/term/GO:0004176) - [metalloendopeptidase activity](https://www.ebi.ac.uk/QuickGO/term/GO:0004222) - [metalloendopeptidase activity](https://www.ebi.ac.uk/QuickGO/term/GO:0004222) | - [meristem maintenance](https://www.ebi.ac.uk/QuickGO/term/GO:0010073) - [proteolysis](https://www.ebi.ac.uk/QuickGO/term/GO:0006508) | Arabidopsis thaliana |
|  |  |  |  | - [ATPase activity](https://www.ebi.ac.uk/QuickGO/term/GO:0016887) - [ATP binding](https://www.ebi.ac.uk/QuickGO/term/GO:0005524) | - [mitochondrion organization](https://www.ebi.ac.uk/QuickGO/term/GO:0007005) | Oryza rufipogon |
| Fe, Cd, Pb | Excalibur_c25090_830  RAC875_rep_c110526_229  Excalibur_c11062_582 | 7B | TraesCS7B02G478200 733527458..733530221 | [Transcription](https://www.uniprot.org/keywords/KW-0804), [Transcription regulation](https://www.uniprot.org/keywords/KW-0805)；MYB-related protein  DNA binding；Myb-like DNA-binding domain |  | Triticum aestivum |
|  |  |  |  | - [ATP binding](https://www.ebi.ac.uk/QuickGO/term/GO:0005524) - [gluconokinase activity](https://www.ebi.ac.uk/QuickGO/term/GO:0046316) | - [D-gluconate catabolic process](https://www.ebi.ac.uk/QuickGO/term/GO:0046177) | Oryza rufipogon |
|  |  |  |  | - [**metal ion binding**](https://www.ebi.ac.uk/QuickGO/term/GO:0046872) - [ubiquitin-protein transferase activity](https://www.ebi.ac.uk/QuickGO/term/GO:0004842) - [protein dimerization activity](https://www.ebi.ac.uk/QuickGO/term/GO:0046983) |  | Oryza rufipogon |
|  |  |  |  | - [**transmembrane transporter activity**](https://www.ebi.ac.uk/QuickGO/term/GO:0022857) |  | Arabidopsis thaliana |
|  |  |  |  | - [glucosinolate transport](https://www.ebi.ac.uk/QuickGO/term/GO:1901349) | - [phloem glucosinolate loading](https://www.ebi.ac.uk/QuickGO/term/GO:0090449) | Arabidopsis thaliana |
|  |  |  |  | - [aspartic-type endopeptidase activity](https://www.ebi.ac.uk/QuickGO/term/GO:0004190) | - [lipid metabolic process](https://www.ebi.ac.uk/QuickGO/term/GO:0006629) - [**response to cadmium ion**](https://www.ebi.ac.uk/QuickGO/term/GO:0046686) | Arabidopsis thaliana |
|  |  |  |  | - [carbohydrate binding](https://www.ebi.ac.uk/QuickGO/term/GO:0030246) - [beta-galactosidase activity](https://www.ebi.ac.uk/QuickGO/term/GO:0004565) | - [carbohydrate metabolic process](https://www.ebi.ac.uk/QuickGO/term/GO:0005975) | Arabidopsis thaliana |
|  |  |  |  | - [peptide-O-fucosyltransferase activity](https://www.ebi.ac.uk/QuickGO/term/GO:0046922) | - [fucose metabolic process](https://www.ebi.ac.uk/QuickGO/term/GO:0006004) | Arabidopsis thaliana |
|  |  |  |  | - [protein serine/threonine kinase activity](https://www.ebi.ac.uk/QuickGO/term/GO:0004674) - [ATP binding](https://www.ebi.ac.uk/QuickGO/term/GO:0005524) | - [protein phosphorylation](https://www.ebi.ac.uk/QuickGO/term/GO:0006468) | Arabidopsis thaliana |
|  |  |  |  | - [**metal ion binding**](https://www.ebi.ac.uk/QuickGO/term/GO:0046872) - [**[zinc ion binding](https://www.ebi.ac.uk/QuickGO/term/GO:0016491)**](https://www.ebi.ac.uk/QuickGO/term/GO:0008270) - [oxidoreductase activity](https://www.ebi.ac.uk/QuickGO/term/GO:0016491) |  | Arabidopsis thaliana |
|  |  |  |  | - [**transmembrane transporter activity**](https://www.ebi.ac.uk/QuickGO/term/GO:0022857) |  | Arabidopsis thaliana |
|  |  |  |  | - [**calcium ion binding**](https://www.ebi.ac.uk/QuickGO/term/GO:0005509) | - [calcium-mediated signaling](https://www.ebi.ac.uk/QuickGO/term/GO:0019722) - [cellular response to hypoxia](https://www.ebi.ac.uk/QuickGO/term/GO:0071456) | Arabidopsis thaliana |
|  |  |  |  | - [**calcium ion binding**](https://www.ebi.ac.uk/QuickGO/term/GO:0005509) - [**mRNA binding**](https://www.ebi.ac.uk/QuickGO/term/GO:0003729) | - [cellular response to hypoxia](https://www.ebi.ac.uk/QuickGO/term/GO:0071456) - [response to absence of light](https://www.ebi.ac.uk/QuickGO/term/GO:0009646) - [response to mechanical stimulus](https://www.ebi.ac.uk/QuickGO/term/GO:0009612) - [thigmotropism](https://www.ebi.ac.uk/QuickGO/term/GO:0009652) | Arabidopsis thaliana |
|  |  |  |  | - [**metal ion binding**](https://www.ebi.ac.uk/QuickGO/term/GO:0046872) - [protein dimerization activity](https://www.ebi.ac.uk/QuickGO/term/GO:0046983) |  | Oryza sativa Indica Group |
|  |  |  |  | - [heme binding](https://www.ebi.ac.uk/QuickGO/term/GO:0020037) - [**iron ion binding**](https://www.ebi.ac.uk/QuickGO/term/GO:0005506) - [oxidoreductase activity, acting on paired donors, with incorporation or reduction of molecular oxygen](https://www.ebi.ac.uk/QuickGO/term/GO:0016705) |  | Triticum urartu |
| Cd | Tdurum_contig44851_927 | 1B | TraesCS1B02G474800 683459637..683471227 | DNA-binding protein | - [chromatin organization](https://www.ebi.ac.uk/QuickGO/term/GO:0006325) | Triticum aestivum |
|  |  |  |  | - [ATP binding](https://www.ebi.ac.uk/QuickGO/term/GO:0005524) - [protein kinase activity](https://www.ebi.ac.uk/QuickGO/term/GO:0004672) |  | Oryza rufipogon  Oryza sativa Indica |
|  |  |  |  | - [copper ion binding](https://www.ebi.ac.uk/QuickGO/term/GO:0005507) - [hydroquinone:oxygen oxidoreductase activity](https://www.ebi.ac.uk/QuickGO/term/GO:0052716) | - [lignin catabolic process](https://www.ebi.ac.uk/QuickGO/term/GO:0046274) | Oryza rufipogon  Oryza sativa Indica  Oryza sativa Japonica |
|  |  |  |  | - [metal ion binding](https://www.ebi.ac.uk/QuickGO/term/GO:0046872) - [RNA methyltransferase activity](https://www.ebi.ac.uk/QuickGO/term/GO:0008173) - [transcription regulatory region sequence-specific DNA binding](https://www.ebi.ac.uk/QuickGO/term/GO:0000976) | - [RNA processing](https://www.ebi.ac.uk/QuickGO/term/GO:0006396) | Oryza sativa Indica |
|  |  |  |  | - [ligase activity](https://www.ebi.ac.uk/QuickGO/term/GO:0016874) - [metal ion binding](https://www.ebi.ac.uk/QuickGO/term/GO:0046872) - [ubiquitin-protein transferase activity](https://www.ebi.ac.uk/QuickGO/term/GO:0004842) | - [organelle organization](https://www.ebi.ac.uk/QuickGO/term/GO:0006996) - Expression Atlasi A0A1P8ANZ5﻿, baseline and differential | Arabidopsis thaliana |
|  |  |  |  | - [**copper ion binding**](https://www.ebi.ac.uk/QuickGO/term/GO:0005507) | - [copper ion binding](https://www.ebi.ac.uk/QuickGO/term/GO:0005507) - ExpressionAtlasi Q5N7B3﻿, baseline and differential | Oryza sativa Japonica |
| Cd | Tdurum_contig44851_593 | 1D | TraesCS1D02G448800 491040912..491041587 TraesCS1D02G448700 | WAS/WASL-interacting family protein | - [chromatin organization](https://www.ebi.ac.uk/QuickGO/term/GO:0006325) | Triticum aestivum |
|  |  |  |  | - [heme binding](https://www.ebi.ac.uk/QuickGO/term/GO:0020037) - [**iron ion binding**](https://www.ebi.ac.uk/QuickGO/term/GO:0005506) - [monooxygenase activity](https://www.ebi.ac.uk/QuickGO/term/GO:0004497) - [oxidoreductase activity, acting on paired donors, with incorporation or reduction of molecular oxygen](https://www.ebi.ac.uk/QuickGO/term/GO:0016705) | - ExpressionAtlasi A0A178WAP8﻿, Q7G9K0，Q9SX95 baseline and differential | Arabidopsis thaliana |
|  |  |  |  | - [ATP binding](https://www.ebi.ac.uk/QuickGO/term/GO:0005524) - [**calcium ion binding**](https://www.ebi.ac.uk/QuickGO/term/GO:0005509) - [protein kinase activity](https://www.ebi.ac.uk/QuickGO/term/GO:0004672) |  | Triticum urartu |
|  |  |  |  | - [ADP binding](https://www.ebi.ac.uk/QuickGO/term/GO:0043531) - [NAD(P)+ nucleosidase activity](https://www.ebi.ac.uk/QuickGO/term/GO:0050135) - [NAD+ nucleotidase, cyclic ADP-ribose generating](https://www.ebi.ac.uk/QuickGO/term/GO:0061809) | - [defense response](https://www.ebi.ac.uk/QuickGO/term/GO:0006952) - [signal transduction](https://www.ebi.ac.uk/QuickGO/term/GO:0007165) - ExpressionAtlasi Q9LSX6﻿, baseline and differential | Arabidopsis thaliana |
|  |  |  |  | - [mRNA binding](https://www.ebi.ac.uk/QuickGO/term/GO:0003729) - [rRNA binding](https://www.ebi.ac.uk/QuickGO/term/GO:0019843) - [structural constituent of ribosome](https://www.ebi.ac.uk/QuickGO/term/GO:0003735) | - [adaxial/abaxial pattern specification](https://www.ebi.ac.uk/QuickGO/term/GO:0009955) - [chloroplast rRNA processing](https://www.ebi.ac.uk/QuickGO/term/GO:1901259) - [plastid translation](https://www.ebi.ac.uk/QuickGO/term/GO:0032544) - [**response to cadmium ion**](https://www.ebi.ac.uk/QuickGO/term/GO:0046686) - [response to cold](https://www.ebi.ac.uk/QuickGO/term/GO:0009409) - [translation](https://www.ebi.ac.uk/QuickGO/term/GO:0006412) - ExpressionAtlasi P93014﻿, baseline and differential - Genevisiblei P93014﻿, AT | Arabidopsis thaliana |
|  |  |  |  | - [**metal ion binding**](https://www.ebi.ac.uk/QuickGO/term/GO:0046872) - [protein serine/threonine phosphatase activity](https://www.ebi.ac.uk/QuickGO/term/GO:0004722) | - [dephosphorylation](https://www.ebi.ac.uk/QuickGO/term/GO:0016311) - [gibberellic acid mediated signaling pathway](https://www.ebi.ac.uk/QuickGO/term/GO:0009740) - [protein dephosphorylation](https://www.ebi.ac.uk/QuickGO/term/GO:0006470) - [protein phosphorylation](https://www.ebi.ac.uk/QuickGO/term/GO:0006468) - [red light signaling pathway](https://www.ebi.ac.uk/QuickGO/term/GO:0010161) - [regulation of growth](https://www.ebi.ac.uk/QuickGO/term/GO:0040008) - ExpressionAtlasi P48484﻿, baseline and differential - Genevisiblei P48484﻿, AT | Arabidopsis thaliana |
|  |  |  |  | - [adenosylmethionine-8-amino-7-oxononanoate transaminase activity](https://www.ebi.ac.uk/QuickGO/term/GO:0004015) - [ATP binding](https://www.ebi.ac.uk/QuickGO/term/GO:0005524) - [dethiobiotin synthase activity](https://www.ebi.ac.uk/QuickGO/term/GO:0004141) - [magnesium ion binding](https://www.ebi.ac.uk/QuickGO/term/GO:0000287) - [protein homodimerization activity](https://www.ebi.ac.uk/QuickGO/term/GO:0042803) - [pyridoxal phosphate binding](https://www.ebi.ac.uk/QuickGO/term/GO:0030170) | - [biotin biosynthetic process](https://www.ebi.ac.uk/QuickGO/term/GO:0009102) - Genevisiblei Q6ZKV8﻿, OS | Oryza sativa japonica |
| Cd | Tdurum_contig13489_292 | 4A | TraesCS4A02G328900 614241223..614243111 | - [DNA binding](https://www.ebi.ac.uk/QuickGO/term/GO:0003677) - cobalt ion-binding protein | - [double-strand break repair via homologous recombination](https://www.ebi.ac.uk/QuickGO/term/GO:0000724) | Triticum aestivum |
| Cd | Kukri_c59197_207 | 4B | TraesCS4B02G004800 3337173..3342340 | - [ADP binding](https://www.ebi.ac.uk/QuickGO/term/GO:0043531) - [ATP binding](https://www.ebi.ac.uk/QuickGO/term/GO:0005524) - NBS-LRR-like resistance protein | - [defense response](https://www.ebi.ac.uk/QuickGO/term/GO:0006952) | Triticum aestivum |
|  |  |  |  | - [ATPase activity](https://www.ebi.ac.uk/QuickGO/term/GO:0016887) - [ATPase-coupled transmembrane transporter activity](https://www.ebi.ac.uk/QuickGO/term/GO:0042626) - [ATP binding](https://www.ebi.ac.uk/QuickGO/term/GO:0005524) | - [embryo development ending in seed dormancy](https://www.ebi.ac.uk/QuickGO/term/GO:0009793) - [thylakoid membrane organization](https://www.ebi.ac.uk/QuickGO/term/GO:0010027) | Oryza sativa japonica |
|  |  |  |  | - [**copper ion binding**](https://www.ebi.ac.uk/QuickGO/term/GO:0005507) |  | Oryza sativa japonica |
|  |  |  |  | - [protein O-GlcNAc transferase activity](https://www.ebi.ac.uk/QuickGO/term/GO:0097363) | - [protein glycosylation](https://www.ebi.ac.uk/QuickGO/term/GO:0006486) | Oryza rufipogon |
|  |  |  |  | - [peptidyl-prolyl cis-trans isomerase activity](https://www.ebi.ac.uk/QuickGO/term/GO:0003755) |  | Oryza rufipogon |
|  |  |  |  | - [ATP binding](https://www.ebi.ac.uk/QuickGO/term/GO:0005524) - [DNA binding](https://www.ebi.ac.uk/QuickGO/term/GO:0003677) - [DNA topoisomerase type II (double strand cut, ATP-hydrolyzing) activity](https://www.ebi.ac.uk/QuickGO/term/GO:0003918) - [metal ion bindin](https://www.ebi.ac.uk/QuickGO/term/GO:0046872)g | - [DNA topological change](https://www.ebi.ac.uk/QuickGO/term/GO:0006265) - ExpressionAtlasi A0A1I9LTQ6﻿, baseline and differential | Arabidopsis thaliana |
|  |  |  |  | - [**4 iron, 4 sulfur cluster binding**](https://www.ebi.ac.uk/QuickGO/term/GO:0051539) - [ATP binding](https://www.ebi.ac.uk/QuickGO/term/GO:0005524) | - [iron-sulfur cluster assembly](https://www.ebi.ac.uk/QuickGO/term/GO:0016226) - [mitochondrial respiratory chain complex I assembly](https://www.ebi.ac.uk/QuickGO/term/GO:0032981) | Arabidopsis thaliana |
|  |  |  |  | - [**manganese ion** transmembrane transporter activity](https://www.ebi.ac.uk/QuickGO/term/GO:0005384) | - [cellular manganese ion homeostasis](https://www.ebi.ac.uk/QuickGO/term/GO:0030026) - ExpressionAtlasi A0A178V2Z5﻿, baseline and differential | Arabidopsis thaliana |
| Cd, Pb | wsnp_Ex_c28908_37989067  wsnp_Ku_c1254_2498515 | 5A | TraesCS5A02G014600  9840158..9857129 | - WD-repeat protein | - [cytoskeleton organization](https://www.ebi.ac.uk/QuickGO/term/GO:0007010) - [regulation of cell shape](https://www.ebi.ac.uk/QuickGO/term/GO:0008360)[regulation of transcription by RNA polymerase II](https://www.ebi.ac.uk/QuickGO/term/GO:0006357) | Triticum aestivum |
|  |  |  |  | - [alcohol dehydrogenase (NAD+) activity](https://www.ebi.ac.uk/QuickGO/term/GO:0004022) - [S-(hydroxymethyl)glutathione dehydrogenase activity](https://www.ebi.ac.uk/QuickGO/term/GO:0051903) - [S-nitrosoglutathione reductase activity](https://www.ebi.ac.uk/QuickGO/term/GO:0080007) - [**zinc ion binding**](https://www.ebi.ac.uk/QuickGO/term/GO:0008270) | - [cell death](https://www.ebi.ac.uk/QuickGO/term/GO:0008219) - [ethanol oxidation](https://www.ebi.ac.uk/QuickGO/term/GO:0006069) - [formaldehyde metabolic process](https://www.ebi.ac.uk/QuickGO/term/GO:0046292) - [heat acclimation](https://www.ebi.ac.uk/QuickGO/term/GO:0010286) - [seed development](https://www.ebi.ac.uk/QuickGO/term/GO:0048316) | Oryza sativa indica |
|  |  |  |  | - [cation transmembrane transporter activity](https://www.ebi.ac.uk/QuickGO/term/GO:0008324) - [manganese:proton antiporter activity](https://www.ebi.ac.uk/QuickGO/term/GO:0010486) - [manganese ion transmembrane transporter activity](https://www.ebi.ac.uk/QuickGO/term/GO:0005384) | - [cellular manganese ion homeostasis](https://www.ebi.ac.uk/QuickGO/term/GO:0030026) - [response to copper ion](https://www.ebi.ac.uk/QuickGO/term/GO:0046688) - [response to manganese ion](https://www.ebi.ac.uk/QuickGO/term/GO:0010042) | Arabidopsis thaliana |
|  |  |  |  | - [cation transmembrane transporter activity](https://www.ebi.ac.uk/QuickGO/term/GO:0008324) | - [zinc ion import across plasma membrane](https://www.ebi.ac.uk/QuickGO/term/GO:0071578) | Arabidopsis thaliana |
|  |  |  |  | - [magnesium ion binding](https://www.ebi.ac.uk/QuickGO/term/GO:0000287) - [ribose phosphate diphosphokinase activity](https://www.ebi.ac.uk/QuickGO/term/GO:0004749) | - [nucleoside metabolic process](https://www.ebi.ac.uk/QuickGO/term/GO:0009116) - [nucleotide biosynthetic process](https://www.ebi.ac.uk/QuickGO/term/GO:0009165) | Arabidopsis thaliana |
|  |  |  |  | - [ATP binding](https://www.ebi.ac.uk/QuickGO/term/GO:0005524) - [kinase activity](https://www.ebi.ac.uk/QuickGO/term/GO:0016301) - [magnesium ion binding](https://www.ebi.ac.uk/QuickGO/term/GO:0000287) - [ribose phosphate diphosphokinase activity](https://www.ebi.ac.uk/QuickGO/term/GO:0004749) | - [5-phosphoribose 1-diphosphate biosynthetic process](https://www.ebi.ac.uk/QuickGO/term/GO:0006015) - [nucleoside metabolic process](https://www.ebi.ac.uk/QuickGO/term/GO:0009116) - [nucleotide biosynthetic process](https://www.ebi.ac.uk/QuickGO/term/GO:0009165) - [purine nucleotide biosynthetic process](https://www.ebi.ac.uk/QuickGO/term/GO:0006164) | Arabidopsis thaliana |
|  |  |  |  | - [acetyl-CoA C-acetyltransferase activity](https://www.ebi.ac.uk/QuickGO/term/GO:0003985) - [acetyl-CoA C-acyltransferase activity](https://www.ebi.ac.uk/QuickGO/term/GO:0003988) - [metal ion binding](https://www.ebi.ac.uk/QuickGO/term/GO:0046872) | - [fatty acid beta-oxidation](https://www.ebi.ac.uk/QuickGO/term/GO:0006635) - [isoprenoid biosynthetic process](https://www.ebi.ac.uk/QuickGO/term/GO:0008299) - [pollen germination](https://www.ebi.ac.uk/QuickGO/term/GO:0009846) - [pollen tube growth](https://www.ebi.ac.uk/QuickGO/term/GO:0009860) - [sterol metabolic process](https://www.ebi.ac.uk/QuickGO/term/GO:0016125) | Arabidopsis thaliana |
|  |  |  |  | - [manganese ion binding](https://www.ebi.ac.uk/QuickGO/term/GO:0030145) - [nutrient reservoir activity](https://www.ebi.ac.uk/QuickGO/term/GO:0045735) |  | Arabidopsis thaliana |
|  |  |  |  | - [ATP binding](https://www.ebi.ac.uk/QuickGO/term/GO:0005524) - [structural constituent of cytoskeleton](https://www.ebi.ac.uk/QuickGO/term/GO:0005200) | - [response to cytokinin](https://www.ebi.ac.uk/QuickGO/term/GO:0009735) - [response to far red light](https://www.ebi.ac.uk/QuickGO/term/GO:0010218) - [response to high light intensity](https://www.ebi.ac.uk/QuickGO/term/GO:0009644) - [response to red light](https://www.ebi.ac.uk/QuickGO/term/GO:0010114) - [root epidermal cell differentiation](https://www.ebi.ac.uk/QuickGO/term/GO:0010053) - [root hair cell tip growth](https://www.ebi.ac.uk/QuickGO/term/GO:0048768) - [root hair elongation](https://www.ebi.ac.uk/QuickGO/term/GO:0048767) | Arabidopsis thaliana |
